# Disruption of FDPS/Rac1 axis radiosensitizes pancreatic ductal adenocarcinoma by attenuating DNA damage response and immunosuppressive signalling

**DOI:** 10.1016/j.ebiom.2021.103772

**Published:** 2021-12-28

**Authors:** Parthasarathy Seshacharyulu, Sushanta Halder, Ramakrishna Nimmakayala, Satyanarayana Rachagani, Sanjib Chaudhary, Pranita Atri, Ramakanth Chirravuri-Venkata, Michel M. Ouellette, Joseph Carmicheal, Shailendra K. Gautam, Raghupathy Vengoji, Shuo Wang, Sicong Li, Lynette Smith, Geoffrey A. Talmon, Kelsey Klute, Quan Ly, Bradley N Reames, Jean L Grem, Lyudmyla Berim, James C Padussis, Sukhwinder Kaur, Sushil Kumar, Moorthy P. Ponnusamy, Maneesh Jain, Chi Lin, Surinder K Batra

**Affiliations:** a Department of Biochemistry and Molecular Biology, University of Nebraska Medical Center, Omaha, NE 68198-5870, USA; b Department of Radiation Oncology, University of Nebraska Medical Center, Omaha, NE 68198-6861, USA; c Department of Statistics, University of Nebraska Medical Center, Omaha, NE, USA; d Department of Pathology and Microbiology, University of Nebraska Medical Center, Omaha, NE, USA; e Division of Oncology-Hematology, Department of Internal Medicine, University of Nebraska Medical Center, Omaha, NE, USA; f Division of Surgical Oncology, Fred and Pamela Buffett Cancer Center, University of Nebraska Medical Center, Omaha, NE, USA; g Division of Gastroenterology and Hepatology, Department of Internal Medicine, University of Nebraska Medical Center, Omaha, NE, USA; h Division of Medical Oncology, Rutgers Cancer Institute of New Jersey, Rutgers Robert Wood Johnson Medical School, Rutgers University, New Brunswick, NJ, USA; i Fred and Pamela Buffet Cancer Center, Eppley Institute for Research in Cancer and Allied Diseases, University of Nebraska Medical Center, Omaha, NE, USA

**Keywords:** FDPS, PDAC, Radioresistance, Zoledronic acid, Radiotherapy

## Abstract

**Background:**

Radiation therapy (RT) has a suboptimal effect in patients with pancreatic ductal adenocarcinoma (PDAC) due to intrinsic and acquired radioresistance (RR). Comprehensive bioinformatics and microarray analysis revealed that cholesterol biosynthesis (CBS) is involved in the RR of PDAC. We now tested the inhibition of the CBS pathway enzyme, farnesyl diphosphate synthase (FDPS), by zoledronic acid (Zol) to enhance radiation and activate immune cells.

**Methods:**

We investigated the role of FDPS in PDAC RR using the following methods: *in vitro* cell-based assay, immunohistochemistry, immunofluorescence, immunoblot, cell-based cholesterol assay, RNA sequencing, tumouroids (KPC-murine and PDAC patient-derived), orthotopic models, and PDAC patient's clinical study.

**Findings:**

FDPS overexpression in PDAC tissues and cells (*P* < 0.01 and *P* < 0.05) is associated with poor RT response and survival (*P* = 0.024). CRISPR/Cas9 and pharmacological inhibition (Zol) of FDPS in human and mouse syngeneic PDAC cells in conjunction with RT conferred higher PDAC radiosensitivity *in vitro* (*P* < 0.05, *P* < 0.01, and *P* < 0.001) and *in vivo* (*P* < 0.05). Interestingly, murine (*P* = 0.01) and human (*P* = 0.0159) tumouroids treated with Zol+RT showed a significant growth reduction. Mechanistically, RNA-Seq analysis of the PDAC xenografts and patients-PBMCs revealed that Zol exerts radiosensitization by affecting Rac1 and Rho prenylation, thereby modulating DNA damage and radiation response signalling along with improved systemic immune cells activation. An ongoing phase I/II trial (NCT03073785) showed improved failure-free survival (FFS), enhanced immune cell activation, and decreased microenvironment-related genes upon Zol+RT treatment.

**Interpretation:**

Our findings suggest that FDPS is a novel radiosensitization target for PDAC therapy. This study also provides a rationale to utilize Zol as a potential radiosensitizer and as an immunomodulator in PDAC and other cancers.

**Funding:**

National Institutes of Health (P50, P01, and R01).

**Research in context**

**Evidence before this study**

Pancreatic ductal adenocarcinoma (PDAC) patients typically respond to radiotherapy (RT) initially. However, cancer cells eventually acquire radioresistance (RR) through metabolic reprogramming of cholesterol biosynthesis pathway (CBS) genes, specifically, farnesyl diphosphate synthase (FDPS). Thus, RT enhances FDPS expression, which modulates Rac1 and DNA damage signals and decreases the radio-responsiveness of PDAC cells.

**Added value of this study**

Our study is the first study to (1) investigate the role of FDPS in PDAC radioresistance and poor survival, (2) assess the utility of Zoledronic acid (Zol), a specific inhibitor of FDPS, conferred significant radiosensitization effects in PDAC cells, 3D murine KPC, and human PDAC tumouroids and *in vivo* models, and (3) our pre-clinical and ongoing clinical study evaluating the safety and efficacy of Zol as a radiosensitizer will provide proof of principle for FDPS as a novel target for radiosensitization with a defined underlying mechanism. Furthermore, we show that Zol+RT treatment showed excelled tumour pathological response with an improved systemic immune cell activation and expanded variants of γδ T cells.

**Implications of all the available evidence**

Collectively, our study suggests that FDPS is a novel radiosensitization target for PDAC. The presented work also provides a solid rationale to investigate the use of Zol as a potential radiosensitizer and immunomodulator that can be exploited in conjunction with checkpoint blockade agents to prevent radioresistance and improve radiation therapy in other cancers.

Alt-text: Unlabelled box

## Introduction

PDAC is the third most deadly disease and requires new therapeutic strategies.^1^ Resection is the only curative therapy for PDAC, but less than 20% of patients are eligible for resection at the time of diagnosis, with a five-year overall survival of 10%.^2^,^3^ Radiation therapy (RT) is commonly used in the neoadjuvant setting and as consolidative therapy for patients with locally advanced diseases. Since recurrence rates after resection are high, additional therapy is often considered, particularly in the neoadjuvant setting. Thus, to improve survival in patients with PDAC, RT is frequently used alone and in conjunction with chemotherapy before pancreatic surgery. However, 80% of PDAC patients fail to achieve an objective response due to intrinsic and acquired radioresistance (RR).^4^ Furthermore, the role of radiosensitizing chemotherapy remains controversial, and the optimal regimen remains to be defined.^5^,^6^ Hence, there is an unmet need to discover novel radiosensitizers to overcome RR and improve resectability and survival of PDAC patients.

Previously, we identified the expression of several cholesterol biosynthesis (CBS) and few fatty acid biosynthesis pathway genes, most notably FDPS, to be associated with RR in PDAC.^7^ FDPS is a crucial enzyme of the mevalonate pathway that catalyzes the production of geranyl pyrophosphate and farnesyl pyrophosphate from isopentenyl pyrophosphate and dimethylallyl pyrophosphate. Importantly, increased expression of FDPS was found to be associated with several malignancies, such as prostate cancer, glioblastoma, colon cancer, and PDAC.^8^, ^9^, ^10^, ^11^ When FDPS is overexpressed in tumour tissues, its activity increases and correlates with activation of growth-promoting signalling pathways such as AKT, ERK, and STAT3.^9^ Similarly, forced overexpression of FDPS in cancer cells resulted in activation of ERK and AKT pathways in prostate cancer.^8^ A recent study investigated the ability of FDA-approved drugs to target CBS pathway-associated proteins. In the screening, nitrogen-containing bisphosphonate, such as zoledronic acid (Zol), was identified as a candidate inhibitor of FDPS.^10^ In preclinical 3D tumouroid models, treatment with Zol attenuated tumouroid growth.^8^ Due to its inhibitory effects on osteoclasts, Zol has been primarily investigated as a palliative treatment for cancers that are related to bone (e.g., osteosarcomas), associated with bone metastasis (e.g., breast, prostate, and lung), and causing bone turnover (multiple myeloma).^12^, ^13^, ^14^ In multiple myeloma, Zol was shown to reduce the risk associated with mortality by 25%, along with a reduction of skeletal-related events by 25%, compared with pamidronate, another bisphosphonate approved for multiple myeloma treatment.^15^

The objective of the current study was to test the radiosensitization potential of Zol in PDAC cells, mouse and patient-derived PDAC tumouroids, and *in vivo* mouse models. Mechanistically, our RNA-Seq and *in silico* analysis demonstrate that Zol treatment impairs expression of DNA damage and repair proteins, thereby enhancing the radiosensitivity of PDAC cells. We also validated that genetic and pharmacological disruption of FDPS sensitizes human and mouse PDAC to RT. Further, we extended our *in vitro* and *in vivo* observations to clinical settings. In a phase I/II clinical trial, we evaluated the safety and efficacy, of Zol concurrently with hypofractionated RT/5FU or capecitabine in patients with borderline resectable and locally advanced PDAC and studies its impact on overall survival and surgical resection rate. We further characterized the Zol-associated tumour radiation response and imunomodulatory effects.

## Methods

### Ethics

Mouse studies were performed under our institutional Animal Review Committee-approved protocol and ARRIVE guidelines. The clinical trial is approved by the Institution review board (IRB) of UNMC (IRB 552-16) and is registered at clinicalTrials.gov (NCT03073785). Written informed consent was obtained from all patients after receiving induction chemotherapy FOLFIRINOX or gemcitabine/Abraxane.

### Immunohistochemistry and immunofluorescence

Human PDAC tissues were obtained from the UNMC tissue bank with a previous history of radiation and chemotherapy treatment. Based on the pathological scoring, PDAC tissues were categorized as poor responders with pathological scores 0-3, intermediate responders (4-6), and good responders (7-9). Serial sections were stained for FDPS (1:100, Proteintech, USA, 16129-1-AP) and Ki67 antibodies and evaluated for percentage and intensity of staining. The intensity of DAB (3, 3’-diaminobenzidine) staining was graded on a 0-3 (No or negative staining (0), weak (1), moderate (2), and high or intense (3)) scoring system. The percentage of cells staining was scored semi-quantitatively, ranging from 1-4, where 1-25% (1), 26–50% (2), 51-75%, and 76-100% (4) as the maximum region of staining. Similarly, xenograft tissues were stained with Rad50, pP95, Ki67 rabbit (1:100, Cell signaling technology # 9449), and cleaved caspases-3 (1:200, Cell signaling technology # 9661) specific antibodies and evaluated for staining as described in our previous publications.^7^,^8^,^16^ For ferroptosis assessment, PDAC cells were seeded on sterile coverslips and treated with the indicated concentration of Zol and/or RT. Lipid peroxidation was measured by staining using C11 BODIPY, and immunofluorescence images were taken using a laser-scanning confocal microscope (LSCM).

### Cell culture, reagents, and transfection

All the human and mouse PDAC cell lines were regularly inspected for mycoplasma contamination and maintained in DMEM media supplemented with 10% FBS and antibiotics penicillin (100 U/mL) and streptomycin (100 mg/mL) as described previously.^7^,^16^ Luciferase-tagged T3M4, CD18/HPAF, and AsPC-1 PDAC cells human PDAC cell lines, HPNE normal immortalized pancreatic cells, and CD18/HPAF ectopically overexpressed with constitutive active Rac1 mutant (Rac1^G12V^) were provided by Dr. Ouellet (UNMC). Capan-1, SW1990, BxPC-3, Suit-2, Colo-357, MIA PaCa, PDAC, and normal immortalized ductal epithelial pancreatic (HPDE) cells were purchased from American Type Culture Collection and routinely maintained in the laboratory. Mouse-derived syngeneic cell lines were derived from the PDAC tumour-bearing mouse pancreas with conditional activation of LSL-Kras^G12D/+^; LSL-Trp53^R172H/+^; Pdx-1-cre, were maintained in a similar complete media. The HPNE cell lines were maintained in Keratinocyte medium supplemented with 0.05 mg/ml of bovine pituitary extract and 5 ng/ml of EGF. All the cells were maintained as monolayers in the media, as mentioned earlier, at 37°C with 5% CO_2_.

### FDPS-targeting construct (gRNA and siRNA), transfection, and zoledronic acid

Guide RNAs targeting FDPS were designed to the coding sequence of FDPS (ENST00000356657) corresponding to 1,555 bp encoding a protein of 419 amino acids. Subsequently, the gRNAs were purchased from GenScript. The construct bearing pSpCas9 BB-2A-GFP (PX458) was transfected into T3M4 and CD18/HPAF PC cell lines using Lipofectamine 2000 (Invitrogen, CA, USA). Following multiple passages, GFP-positive cells were flow-sorted, and clones were developed in 96-well plates. Selected clones were validated for knockdown efficiency and pooled to obtain a heterogeneous population for further analysis. For transient transfection studies, FDPS targeting siRNA (27 mer) and scramble siRNA was purchased from Origene^8^ and transfected using lipofectamine 2000 reagent and incubated for 48 h. Zol was purchased from Sigma (St. Louis, MO, USA) and was dissolved in sterile RNase-free water (Qiagen), aliquoted with a working solution of 5-10 mM concentration, and stored at -20 °C.

### Radiation treatment to PDAC cells

PDAC cells were irradiated using a linear accelerator in the Department of Radiation Oncology at UNMC. Radioselected radioresistant (RS-RR) subclones of human PDAC cell lines (T3M4, CD18/HPAF, AsPC-1) were generated from their parental cell lines by irradiating the cells using a fractionated irradiation dose of approximately 2 Gy once a day for five days a week, over a period of two weeks using the linear accelerator. Further, both parental control and RS-PDAC cells were exposed to radiation sources with single doses of 0, 2.5, 5, and 7 Gy for the clonogenic survival assay. After 14 days of incubation, cells in the six-well plate were processed and characterized as described by Boothman et al.^7^,^17^

### Clonogenic survival assay

The sensitivity of human PDAC cells to irradiation was measured using the clonogenic survival assay described by Boothman et al.^7^,^17^ Cells were allowed to grow as a monolayer to 40-60% confluency 24 h before radiation. PDAC cells were seeded into a six-well plate in triplicate at a plating density of approximately 2000 cells/well, 24 h before irradiation. The following day, cells were treated with a single fraction of 0, 2.5, 5, and 7 Gy. The cells were further allowed to grow for 14 days, and the complete medium was changed every alternate day. The endpoint for the clonogenic survival assay was determined by the number of colonies in cultures that were not radiated. Large colonies consisting of 50 or more cancer cells were considered a clone. After 14 days of incubation, the cells were fixed with 100% methanol for 5 min and stained with 0.5% crystal violet in 25% methanol for 1 h, and the excess crystal violet was washed with distilled H_2_O. The stained colonies were converted into digital images using a scanning device, and the images were counted using Image J software analysis. For testing the radiosensitization effect of Zol, PDAC cells were seeded as described earlier, and Zol was added in a dose-dependent manner (0, 1, 5, 10, and 20 µM concentrations) and further incubated for 4 h at 37°C in a humidified atmosphere of 5% CO_2_ before radiation treatment. The control cells were incubated with only 10% DMEM. The cells were harvested, fixed, counted, and the survival fractions at the doses of 0-7 Gy were calculated using the formula described previously.^7^

### Side population analysis

Radioselected and parental PC cells were characterized for the enrichment of cancer stem cells by side population analysis.^16^

### Immunoblot analysis and Rac-1 activity measurement in PDAC cells

For western blot analysis, PDAC cells were plated at a density of 1 × 10^6^ in a 100-mm culture dish and treated with Zol at respective concentrations for 4 hrs before radiation exposure. Cells were harvested 24 h post-radiation/Zol treatment in RIPA buffer (50 mM Tris-HCL, 0.1% SDS, 150 mM NaCl, 0.5% sodium deoxycholate and 1% NP40) containing protease inhibitors (1 mM phenyl-methyl sulphonyl fluoride, 1 µg/ml aprotinin and 1 µg/ml leupeptin) and stored at -80°C at least for 4 h. The lysate was thawed and was mechanically disrupted using 25G 7/8 inch syringe needle and centrifuged at 13 000 r.p.m. at 4°C for 30 min. Proteins in the lysate were quantified using the Bio-Rad DC Protein Assay kit (Bio-Rad Laboratories, Hercules, CA, USA). A total protein from the cell lysates of 40 μg was loaded into each well of a 10-12% SDS-PAGE, resolved, and transferred onto a polyvinylidene fluoride (PVDF) membrane (Millipore). These membranes were probed with the following primary antibodies with respective dilutions overnight at 4°C: FDPS (mouse, 1:1000, proteintech # 16129-1-AP), pERK (rabbit, 1:1000, Cell signaling technology # 9101, Danvers, MA, USA), ERK (mouse, 1:2000, Cell signaling technology # 9102), anti-β-actin (mouse, 1:5000, Sigma # A1978, St Louis, MO, USA), and GAPDH. DNA damage/repair antibodies were purchased from Cell signaling technologies. Following incubation with the appropriate HRP-conjugated secondary antibodies, the membranes were washed in PBST thrice, and signals were detected with a GE ECL Plus kit (GE Healthcare Bio-Sciences, Pittsburgh, PA, USA) and simultaneously exposed to sensitive X-ray films to detect the signals. For Rac-1 activity measurement, PDAC cells were incubated with increasing doses of Zol (0-10 μM) for 24 h, and cell lysates were prepared using lysis buffer containing HEPES buffer (25 mM, pH 7.4), NaCl (150 mM), MgCl_2_ (10 mM), NP-40 (1%), glycerol (2%). EDTA (1 mM), DTT (1 mM), aprotinin (1 μg/ml), leupeptin (1 μg/ml), pepstatin (1 μg/ml), PMSF (1 mM), NAF (1 mM), and Na_3_O_4_V (1 mM). Zol-treated and untreated PC cell lysates were incubated with agarose beads coated with GST-bound PAK1 fusion protein for 1 h. The beads were washed twice with lysis buffer, and GTP-bound Rac-1 was separated in 10% SDS-PAGE and immunoblots were probed using anti-Rac1antibody (23A8, Millipore Sigma). As described previously, we incubated cell lysates with I mM GDP to serve as a negative control. Rac-1 activity assay was adapted from previous publications.^7^,^8^,^16^,^18^

### Calcein AM assay

Similarly, HPNE (3500 cells/well) were incubated with and without Zol and RT, and cell viability was measured using Calcein AM compound that fluoresces in live cells. PDAC cells retention of Calcein AM leads to the excitement of viable cells. The viable cells with green fluoresces were measured using the Incucyte®S5 (Sartorius) live-cell analysis instrument.

### Xenograft studies

We developed and optimized orthotopic implantation of tumour cells with a platinum-based fiducial marker coupled with imaging-guided (luciferase and computed tomography (CT) imaging) RT for treating small animals using SBRT. We utilized a commercially available platinum-based fiducial marker along with the orthotopic implantation of luciferase-labeled PDAC cells. Briefly, 6-8-week-old athymic nude male/female (1:1) mice were injected with 0.25 × 10^6^ cells and implanted with the fiducial marker into the head of the pancreas. After tumour cell implantation, the abdominal wall's fascial layers were closed with catgut sutures, and the skin incision was closed using wound clips. Seven days post-implantation, mice were injected (i.p.) with luciferin and imaged using an IVIS machine. Mice were randomized into four groups, each consisting of 5-6 mice. Group 1 mice were kept as control (no IR treatment), whereas groups 2 and 3 received fractionated irradiation doses of approximately 7 Gy once a day for five days or Zol alone. Group 4 mice received both radiation and pre-treatment with Zol. The implanted xenografts were irradiated using a linear accelerator (to deliver SBRT). To capture acute and chronic effects of Zol, we performed *in vivo* radiosensitization in two different batches. The course of treatment and imaging schedules were the same for both batches. The mice group in batch 1 were euthanized 21 days after treatment initiation, and batch 2 mice were euthanized 4 h after the last radiation exposure.

The tumour size was monitored and measured once per week using an IVIS machine.^7^,^16^ Finally, 21 days post-IR, the mice were euthanized, and primary tumours were resected and divided into two portions. Part of the tissues was flash-frozen for RNA isolation, and the remaining part was fixed in 10%-buffered formalin for histopathological analysis. To avoid gender-based biases, we employed an equal number of age-matched male and female athymic nude mice (biological variables).

### RNA isolation, transcriptome, ***in silico***, and RT-qPCR analysis in xenograft tissues and clinical specimens

Global transcriptome analysis was performed on total RNA isolated from xenograft tissues (CD18/HPAF) treated with and without ZOL and RT. Total RNA was isolated using Qiagen RNAeasy RNA isolation kit (Qiagen, Valencia, CA, USA) and quantitated using a NanoDrop ND 1000 Spectrophotometer (NanoDrop Technologies Inc., Wilmington, DE, USA). Based on the pathological scoring, RNA was isolated from the PDAC patient's tissue (specimen obtained through previous clinical trial (NCT01959672)) and blood (through ongoing clinical trial (NCT03073785)) (RiboPure-Blood Kit). Samples of 2000 ng total RNA were provided to the UNMC genome core facility. cDNA libraries were prepared using the RNA-Seq library kit (Illumina), in which fragmented RNA is hybridized and ligated to adaptors to create adaptor-ligated sequences. Following ligation, the RNA was reverse-transcribed into cDNAs, purified, and size-selected using Agencourt AMPure XP beads to generate a size-specific cDNA. Finally, linear cDNA amplification was performed using limiting cycle PCR, the cDNA fragments obtained were sequenced using HiSeq2500, and sequence data were analysed as reads using the “R” package. Similarly, differentially downregulated genes identified through RNA-seq in xenograft tissues were further validated in total RNA extracted from xenograft tissues and CD18/HPAF cells using RT-qPCR analysis (Table S12).

### RNA-Seq data analysis

All the raw sequence reads were processed using the HISAT2-StringTie-Ballgown protocol to retrieve gene and transcript levels (FPKM). The GRCh38 version of the human genome and GENCODE v38 version of feature annotations guided the alignment and read assignment steps. Differential expression analysis was performed to yield statistically significant features (p-value < 0.05) between treatment groups using the Ballgown R package.^19^ The functional annotation analysis was carried out using Ingenuity Pathway Analysis^TM^ and CIBERSORT analysis.

### Patient study design and treatment regimen

This is an open-label, randomized, single-center phase I/II study. Eligible patients were adults with histologically confirmed PDAC, with either borderline resectable or unresectable primary tumour, no larger than 10 cm. Patients were randomized in a 1:1 Ratio to Arm A: stereotactic body radiotherapy (SBRT) with concurrent 5-fluorouracil (FU) or capecitabine or Arm B: SBRT with concurrent 5FU or Cap and Zol. Patients underwent SBRT (40 Gy to primary, 25-30 to regional lymphatics) in 5 fractions. Zol (4 mg) was administered through an intravenous (I.V) route over 15 minutes on the first day of SBRT. Concurrent chemotherapy was administered as 5FU 225 mg/m^2^/day *via* continuous IV infusion or as CAP administered orally at 825 mg/m^2^ twice daily for 4 weeks, starting on day 1 of SBRT. One month after the combination chemoradiation therapy was completed, all the patients were evaluated with imaging studies for resectability. Upon completion of the protocol, all the patients will be followed every 3 months in the first year, every 4 months in the second year, and every 6 months thereafter.

### Clinical endpoints and assessments

The primary objective was to evaluate the efficacy of SBRT with concurrent Zol treatment, and the primary endpoint is planned to evaluate local control. The secondary objectives include toxicity of Zol with SBRT, local failure-free survival, overall survival, resection rate, and tumour response. Exploratory objectives include the identification of biological outcomes and their correlation with treatment regimens of the patients enrolled in Arms A and B.

### Clinical trial sample size calculation

We estimate that there will be about 20 patients enrolled into each Arm over a 5-year period; the first six patients in the Arm that patients are receiving Zol (Zometa) will also be evaluated for the safety of Zol given concurrently with RT. With conventional SBRT, about 60% of these patients would be expected to develop local recurrence by four months of follow-up. With the radiation therapy and Zol outlined in this protocol, we hope to reduce this rate to 30%. The study design will follow a two-stage design using a response endpoint to determine if the regimen is acceptable. The following monitoring rule will be applied: Optimal two-stage design to test the null hypothesis that *P*<=0.400 versus the alternative that P>=0.700, alpha=0.10, 90% power. After testing the regimen on 11 patients in the first stage, the trial will be terminated if 5 or fewer patients have local control. If the trial goes on to the second stage, a total of 20 patients will be studied. If the total number of patients with local control is less than or equal to 10, the regimen is rejected.

### Blood and tissue sample collection

We have sequentially collected blood samples from 15 PDAC patients randomized to Arm A (only RT/CAP) and 11 patients assigned to Arm B (Zol with RT/CAP). The blood samples collected on day 1 before Zol infusion/RT treatment will be considered a baseline and compared with Day 5 samples. Based on the pathological score on the tissue samples (0 means no response and 9 is the complete response), we randomly selected blood samples from a single patient from both Arm A and Arm B, and peripheral blood mononuclear cells (PBMC) were isolated from whole blood and processed with LSM^TM^ lymphocyte separation medium.^20^ Total RNA was extracted from the isolated PBMCs using the Ambion Ribopure^TM^ RNA purification kit (AM1928). Further, the RNA was processed for transcriptomic analysis as described in RNA Seq. analysis section.

### Tumouroid studies

3D pancreatic tumouroids were established from KPC (25 weeks old) autochthonous mouse models, and human PDAC tissues and tumouroids' growth and responses were measured using methods described previously.^16^

### Cell cycle, apoptosis, ferroptosis, and cell-based cholesterol assay

Human (CD18/HPAF and T3M4) and mouse syngeneic (UN-KPC-961) PDAC cells were exposed to Zol (5μM) for 4 hrs followed by RT (7 GY) treatment for 24 hrs, and harvested cells were stained with Annexin V/propidium iodide (PI) and analysed using FACS. A similar Zol+ RT treatment approach was adapted for cell cycle analysis, as described previously.^21^ PDAC cells were pre-treated with Zol (5 µM), exposed to RT (7 Gy of 1 fraction), fixed in 3% paraformaldehyde follwed by staining with filipin III dye and processed as per manufactures protocol (ab133116). The intracellular variation in cholesterol levels was measured by counting the filipin-positive cells that bind with sterol/cholesterol using the EVOS® FL auto live imaging system (Life technologies).

### Statistical analysis for *in vitro* and *in vivo* studies

An in-house biostatistician performed a detailed statistical analysis. ANOVA was used to compare tumour weights between groups. The ANOVA assumptions were assessed using residual plots, and after natural log transformation, the assumptions appeared to have been met. A linear mixed model was used to compare photons between groups over time. The model included fixed effects for the treatment group, day, and group x day interaction. A random mouse effect was included to account for correlation within the mouse over time. Analysis was done on the natural log scale or square root scale to meet model assumptions (ANOVA). Both in acute and chronic treatment groups, Tukey's method was used to adjust p-values for multiple comparisons at each level of day and group. For the short-term treatment mice, the photons from the xenograft tumours were compared using a linear mixed model, as we have done before. However, we performed statistical analysis on the square root scale to meet model assumptions. Student's *t*-test was used to calculate the statistical difference between groups in all other *in vitro* assays. SAS software version 9.4 was used for data analysis (SAS Institute Inc., Cary, NC). Graphs were generated using GraphPad Prism 8 software. Data are presented as mean±SEM.

### Reagents validation

Luciferase labeled pancreatic cancer cell lines CD18/HPAF, T3M4, AsPC-1, BxPC-3 were obtained from Dr. Ouellette's Lab. The DNA was isolated, PCR amplified, and short tandem repeat (STR) profiling was done with Applied Biosystems. All these pancreatic cancer cells were routinely inspected for their recognizable morphology in a light microscope, growth curve analysis using Trypan blue dye, and proliferation assay using tetrazolium dye 3-(4,5-dimethylthiazol-2-yl)-2,5-diphenyltetrazolium bromide) (MTT). Mycoplasma contamination was detected by incubating the pancreatic cancer cells with 4% paraformaldehyde followed by staining with DAPI solution, and the presence of mycoplasma was viewed through a fluorescent microscope. Si-RNAs and CRISPR guide RNAs targeting FDPS were validated using two independent sequences and clones. All the antibodies were purchased from well-established vendors and matched for the molecular weight of antibody of interest with the respective data sheet provided by the vendors.

### Role of funders

The authors confirm that the funding sources had no role in designing this study, data collection, data analysis, interpretation of results, manuscript preparation, drafting, and decision to submit the manuscript for publication. All authors affirm that this manuscript and the data it contains are original. All the authors had full access to all the results pertaining to this preclinical and clinical study and accepted their responsibility for manuscript submission and publication.

## Results

### FDPS and Ki67 co-expression are associated with poor response to radiotherapy

**Figure 1:**
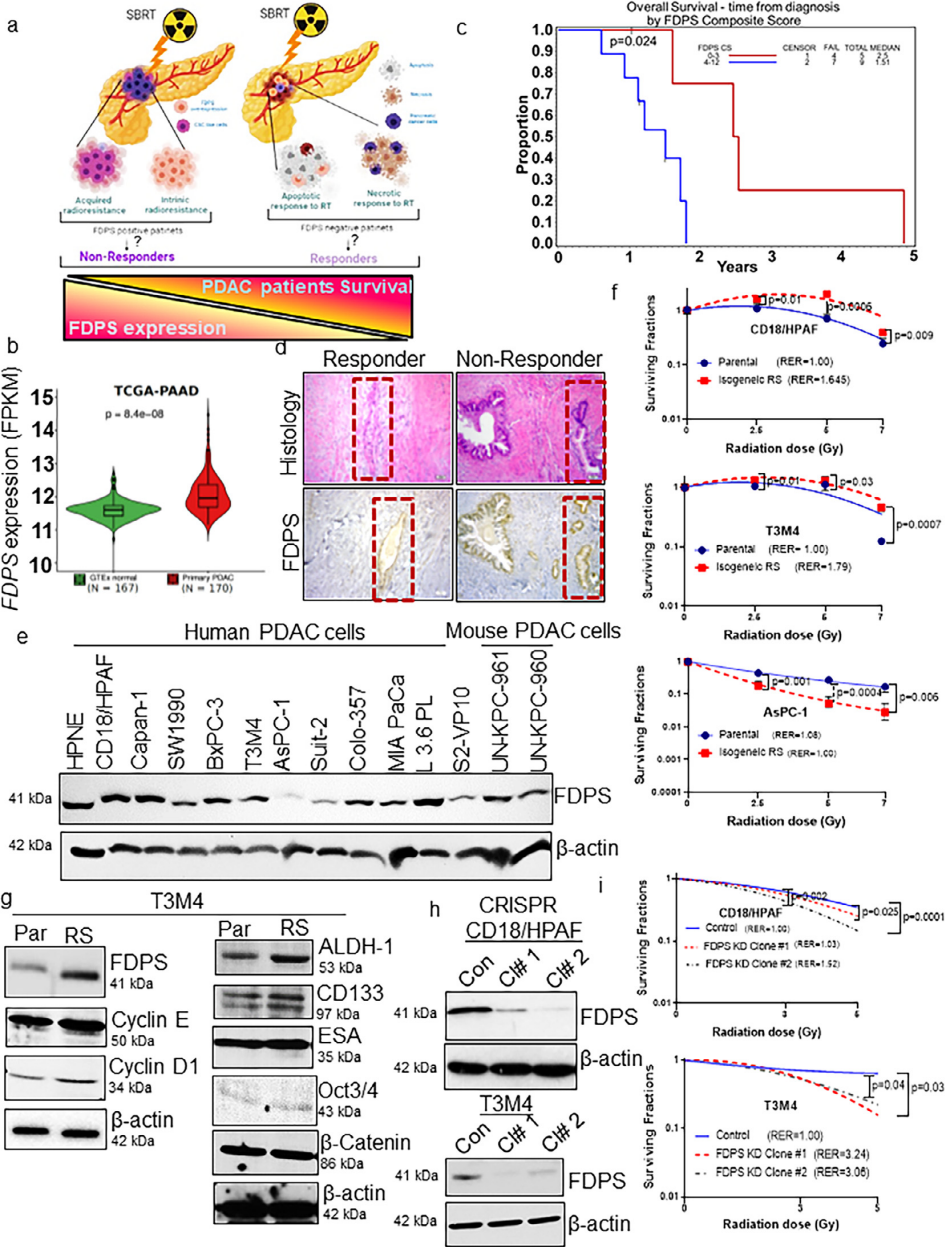
FDPS is overexpressed in pancreatic cancer and predicts the response of PDAC patients and cells to RT. (a) Cartoon representing the major impact of radiation on primary PDAC cells and its association with FDPS expression in radioresponder and non-responder PDAC patients. (b) Box plot illustrates *FDPS* expression (*P* = 8.4e^−08^, student's *t-test*) in TCGA-PDAC (*n* = 170) and GTEx normal pancreas (n= 167) (Courtesy: GEPIA webserver). (c) Kaplan-Meier survival curves illustrate the overall survival difference between PDAC patients on the basis of the FDPS composite score. FDPS (*n* = 14, *P* = 0.024, log-rank test)-positive or high-expressing patients had statistically significant lower survival than negative or low-expressing patients. Tick marks on the graph indicate censored subjects. (d) Representative images show histology and FDPS-positive expression in serial sections of radioresponder and non-responder PDAC tissues. (e) Western blot analysis of FDPS protein expression in normal immortalized pancreatic (HPNE), human PDAC, and mouse syngeneic PDAC cells. (f) Clonogenic survival assay illustrating relative RR of CD18/HPAF *vs.* CD18/HPAF RS (*P* = 0.01 (2.5 Gy), *P* = 0.0006 (5 Gy), *P* = 0.009 (7 Gy), n=3) and T3M4 *vs.* T3M4 RS (*P* = 0.01 (2.5 Gy), *P* = 0.03 (5 Gy), *P* = 0.0007 (7 Gy)) and AsPC-1 *vs.* AsPC-1 RS (*P* = 0.001 (2.5 Gy), *P* = 0.0004 (5 Gy), *P* = 0.006 (7 Gy), n=3) PDAC cells. The survival curves were generated by calculating the plating efficiency and survival fractions of colonies obtained using different plating densities of parental and isogenic PDAC cells. (g) Persistent exposure of PDAC cells to radiation results in increased expression of proteins involved in cell cycle (cyclin D1 and Cyclin E) and enrichment of pancreatic cancer stem cells markers (ALDH1, CD133, OCT3/4, ESA, and β-catenin) and FDPS in RS cells as compared to parental cells. (h) Western blot analyses show a reduction in FDPS protein in the CRISPR/Cas9-selected stable clones compared to passage-matched control cells. Beta actin was used as an internal loading control. (i) Clonogenic survival assay showed that CRISPR/Cas9-mediated FDPS KD reduced the survival fraction of PDAC cells. CRISPR KD Clone 2 (CD18/HPAF) and Clone 1 (T3M4) cells exhibited enhanced sensitivity to RT relative to the parental control [CD18/HPAF (con *vs* Cl#1 (*P* = 0.025 (5 Gy), con *vs* Cl#2 *P* = 0.002 (3 Gy), *P* = 0.0001 (5 Gy)), (n=6), T3M4 (con *vs* Cl#1, *P* = 0.03 (5 Gy), con *vs* Cl#2, *P* = 0.04 (5 Gy)), n=3]. Statistical analysis was done by student's *t-test*.

FDPS expression was demonstrated to be associated with the progression of various cancers, including PDAC.^7^, ^8^, ^9^, ^10^ However, its relationship with PDAC RR and its value as a novel target for PDAC therapy are unknown (Figure 1a). Through global transcriptomic data analysis (PAAD), we found that FDPS transcript levels are significantly higher in pancreatic tumours than in normal pancreas (Figure 1b). Further, to ascertain if FDPS expression influences response to RT, we examined the expression of FDPS in surgically resected PDAC tissues following chemo-radiation treatment (specimens obtained through our institution's previous clinical trial (NCT01959672)). The patient's characteristics and radiation response scores are listed in **Tables S1** and **S2**. The anatomic pathology searched for archived pancreatic resection specimens from patients that underwent neoadjuvant chemotherapy. There was no further enrichment or vetting. Consecutive cases from that pool that demonstrated tumor regression scores of 1, 2, and 3 and contained sufficient residual paraffin-embedded tissue were selected for further study. They represent a cross-section of patients that met criteria during that time span.

The patients were categorized as good responders (7-9), intermediate responders (4-6), and non/poor responders (0-3) based on pathological score. We found that FDPS was overexpressedand predicted the clinical outcome between radiation responders versus non-responders. Also, we detected Ki67-positive cells (proliferative marker) to examine the proliferative index in the serial sections of the same tissues. We found a significant association between high FDPS expression (composite score ≥4) and poor survival (*P* = 0.024) (Figure 1c and d). We also observed that the specimens with high FDPS expression also showed high Ki67 expression (>4% cells positive) and were significantly associated with overall poor survival (*P* = 0.025) of PDAC patients (Figure S1a and b). We also observed that PDAC patients who did not respond to RT and showed the worst survival outcomes had high FDPS expression. As shown in **Figure S1c**, we observed a Spearman correlation of 0.60 (*P* = 0.025) between FDPS and Ki67 co-expression (**Table S3**). Further, transcriptomic analysis of PDAC samples revealed that *FDPS* expression is high in low/non-responders, whereas responders showed low *FDPS* levels (**Figure S1d**). These results need to be further validated in a larger PDAC patient cohort.

### FDPS expression confers enhanced RR of PDAC cells

We compared cellular radiosensitivity between the FDPS-high and FDPS-low expressing PDAC cells. To do this, endogenous expression of FDPS protein in human and mouse PDAC cell lines and normal immortalized pancreatic cell lines were analysed. FDPS protein was distinctly higher in several PDAC cell lines tested than normal HPNE cell lines (Figure 1e) mainly due to its upstream regulation by EGFR amplification and the Kras-mediated AKT/mTOR/SREBP axis in PDAC.^16^,^22^,^23^ Furthermore, radioselected (RS) isogenic PDAC cells (CD18/HPAF, T3M4) expressing high FDPS were less sensitive to RT as compared with AsPC-1 PDAC cells expressing low FDPS (Figures. 1f and S2a). Cellular RR in isogenic cell lines was confirmed by changes in cell morphology, clonogenic survival, enrichment of side-population and cancer stem cell markers (ALDH1, CD133, OCT3/4, ESA, and, β-catenin), pronounced G2/M arrest coupled with the accumulation of cell cycle regulator (cyclin D1 and Cyclin E) along with enrichment of FDPS, and measurable *in vivo* tumour growth with respect to matched parental cells (Figures. 1f and g; S2a–e). These results confirm and validate the effect of RS in the isogenic sublines generated through fractionated radiation relative to non-radiated parental PDAC cells.

### Genetic and pharmacological suppression of FDPS increases the radiosensitivity of PDAC cells

**Figure 2:**
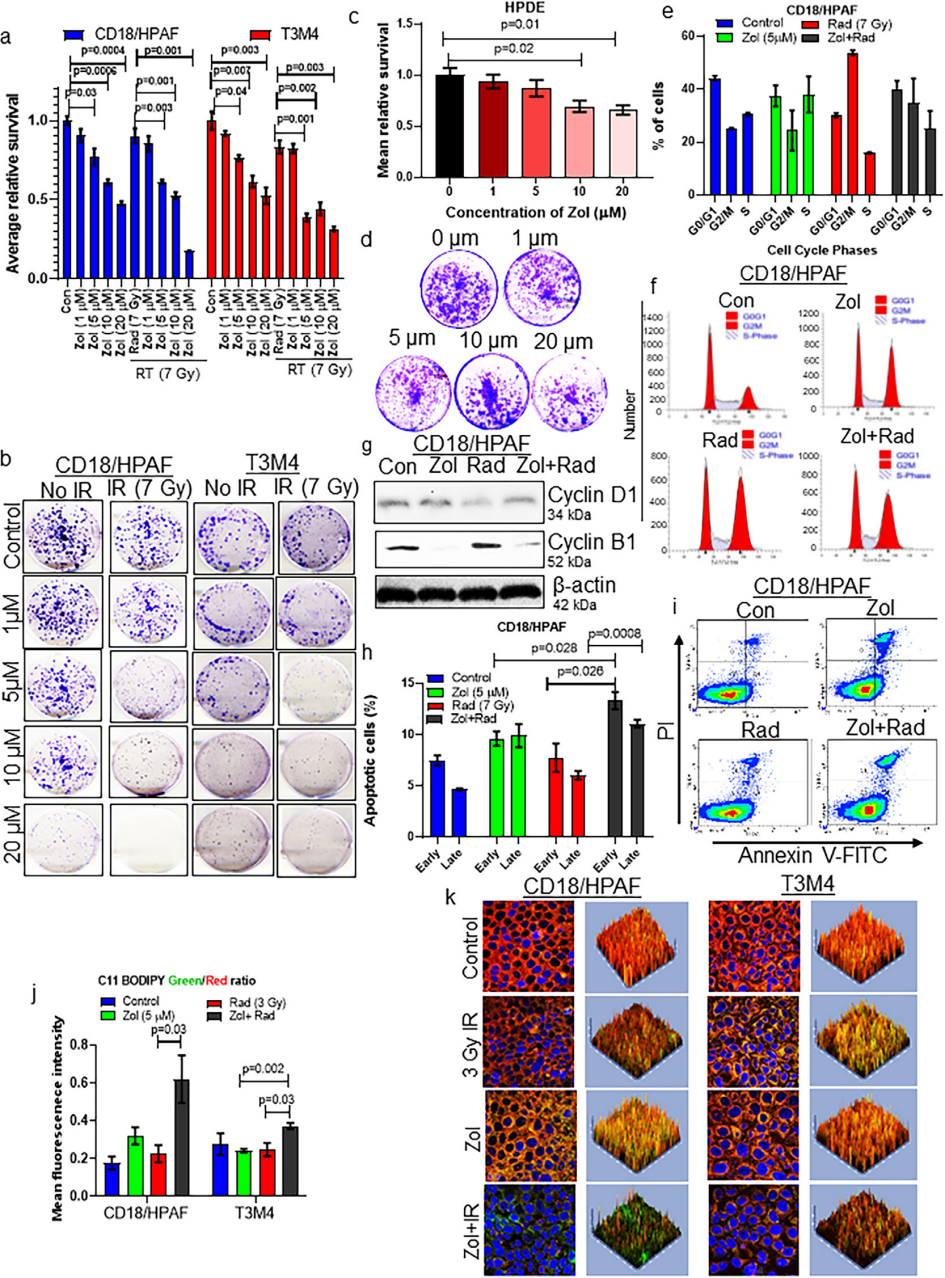
Zol treatment enhances PDAC cell radiosensitivity by affecting the cell cycle and inducing apoptosis. (a and b) Clonogenic survival assays were performed on PDAC cells treated with increasing concentrations (0-40 µM) of Zol followed by RT (7 Gy). (a) The bar graph shows decreased colony numbers as a result of dose-dependent treatment of Zol [CD18/HPAF (con *vs* Zol (5 µM) (*P* = 0.03), con *vs* Zol (10 µM) (*P* = 0.0006), con *vs* Zol (20 µM) (*P* = 0.0004), Rad *vs* Zol (5 µM)+ Rad (*P* = 0.003), Rad *vs* Zol (10 µM)+ Rad (*P* = 0.001), Rad *vs* Zol (20 µM)+ Rad (*P* = 0.001)), T3M4 (con *vs* Zol (5 µM) (*P* = 0.04), con *vs* Zol (10 µM) (*P* = 0.007), con *vs* Zol (20 µM) (*P* = 0.003), Rad *vs* Zol (5 µM)+ Rad (*P* = 0.001), Rad *vs* Zol (10 µM)+ Rad (*P* = 0.002), Rad *vs* Zol (20 µM)+ Rad (*P* = 0.003)), n=3]. (b) Representative images of surviving colonies after Zol and RT treatments. (c and d) Effect of Zol on radiosensitization of normal immortalized pancreatic cells. Normal immortalized HPDE were subjected to various doses of Zol. (c) The bar graph depicts the average number of colonies that survived after Zol treatment, and colony numbers were quantified using Image J software (Con *vs.* Zol (10 µM) (*P* = 0.02, Con *vs.* Zol (20 µM) (*P* = 0.01), n=3). (d) Representative HPDE colonies stained with 0.5% crystal violet in 25% methanol. (e) Bar graphs show the percentage of cells (CD18/HPAF) in each cell cycle phase after Zol and/or RT treatment. (f) Histogram showing percentage of cells distributed in each cell cycle phase in response to Zol and/or RT treatment in PDAC cells. (g) Western blot analysis confirms cyclin D1 and cyclin B1 protein levels following Zol and RT treatment in PDAC cells. (h and i) Exposure of PDAC cells to Zol and RT results in apoptosis induction. (h) The bar graph depicts the percentage of early and late apoptosis as detected by Annexin V and propidium iodide (PI) staining (Zol *vs.* Zol (5 µM) + Rad (*P* = 0.028, early apoptosis), Rad *vs.* Zol (5 µM)+Rad (*P* = 0.026, early apoptosis), Rad *vs.* Zol (5 µM)+Rad (*P* = 0.0008, late apoptosis), n=4). (i) Representative annexin V/PI staining shows post-treatment effects of Zol+RT. Scatter plots depict the density of live (lower-left), early (lower-right), late or necrosis (upper-right), and only necrotic cells (upper-left) in each quadrant. (j and k) Pre-treatment of PDAC cells with Zol followed by RT increased lipid peroxidation. Notably, a form of cell death known as ferroptosis is induced by the lethal build-up of peroxidated lipids. PDAC cells were pre-treated with 5 µM of Zol 4 h before a single fraction of 3 Gy radiation. Cells were fixed 12 h after RT, and lipid peroxidation was assessed *via* C11 BODIPY staining. C11 BODIPY causes the peroxidation of lipids to stain green while non-peroxidated lipids stain red. A ratio of these two colours produces a relative oxidative state of the lipid species between treatment groups. (j) The bar graph demonstrates the increase in mean fluorescence intensity of peroxidated lipids due to Zol+RT treatment compared to Zol or RT alone and untreated control cells [CD18/HPAF (Rad *vs* Zol+Rad (*P* = 0.03)), T3M4 (Zol *vs* Zol+Rad (*P* = 0.002), Rad *vs* Zol+Rad (*P* = 0.03)), n=3]. (k) Representative confocal images demonstrate the variation in normal lipids (Red) and peroxidized lipids (Green). Statistical analysis was done by student's *t-test*.

To analyse the impact of *FDPS* expression on PDAC cell radiosensitivity, we suppressed FDPS expression in CD18/HPAF and T3M4 PDAC cell lines using the CRISPR/Cas9 system. Cells transduced with FDPS-specific sgRNA (via with CRISPR/Cas9) were isolated through FACS analysis (GFP positive), and FDPS knockdown (KD) cells showed a difference in morphology as compared with parental control PDAC cells matched for passage (Figure S3a–c). In both the PDAC cell lines, we observed a decrease in FDPS protein upon KD (Figure 1h), which was linked to increased PDAC cell radiosensitivity (Figures. 1i; S3d). Reduced FDPS expression was also accompanied by decreased phosphorylation of AKT and ERK, but no change in their respective total proteins (**Figure S3e**). Based on previous reports demonstrating Zol can target FDPS,^7^,^8^,^10^,^24^ we wondered that whether Zol can radiosensitize PDAC cells. We found that pre-exposure of human PDAC cells to Zol at 1, and 5 µM could decrease PDAC cells survival without causing a cytocidal effect. Higher concentrations of 10 and 20 µM caused cytotoxicity, as evidenced by reduced or no colonies (Figure 2**a and b**). We also observed a similar significant radiosensitization effect of Zol in murine syngeneic PDAC cells (UN-KPC-961) (**Figure S4a and b)**. In contrast, dose-dependent treatment of normal immortalized human pancreatic ductal epithelial cells (HPDE) with Zol did not affect the survival of colonies at lower concentrations (1 and 5 µM). However, Zol exhibited significantly cytotoxic effects at higher (10 and 20 µM) concentrations (Figure 2**c and d**). Similarly, hTERT immortalized human pancreatic nestin-positive cells (HPNE) also showed radiosensitivity only with higher concentrations of Zol (5 µM) but not with Zol alone exposure or a lower concentration (1 µM) (**Figure S5a and b)**. We used a cell-based cholesterol assay to predict the FDPS inhibitory activity of Zol. Treatment with either Zol alone or with RT reduced intracellular cholesterol levels compared to control and radiation-treated PDAC cells (**Figure S6a and b**). Overall, these results indicate that targeting FDPS by Zol could radiosensitize PDAC cells but will exhibit radiosensitizing effects only at higher concentrations and would not affect FDPS activity as a single agent in normal pancreatic cells. Hence, targeting FDPS using Zol will sensitize pancreatic tumours effectively without causing side effects to normal uninvolved pancreatic cells.

### Zol enhances radiosensitivity by abrogating radiation-induced G2/M arrest accompanied by induction of apoptosis

We speculated that Zol treatment enhances radioresponse by manipulating the cell cycle and apoptosis in PDAC cells.^25^ We found that radiation alone induced G2/M arrest and Zol alone enhanced S phase arrest. However, when both human and murine PDAC cells were pre-treated with Zol followed by radiotherapy, they decreased G2/M and S phase arrest. These data suggest that Zol abrogates RT-induced G2/M arrest (Figures. 2**e, f; S7a–c**). Furthermore, the cell cycle changes upon Zol and RT treatment were reflected in decreased cyclin D1 and cyclin B1 protein expression in both human and mouse PDAC cells (Figures. 2**G; S7d)**. Complementing this data, pre-treatment of human and mouse PDAC cells with Zol for 4 hrs followed by RT resulted in a significant increase in apoptosis compared to Zol or RT alone (Figures. 2**h, i; S4c and d**). Furthermore, compared with single treatments, the combination treatment also increased lipid peroxidation, the build-up of which facilitates a form of programmed cell death known as ferroptosis^26^ (Figure 2**j and k**). These data demonstrate that Zol radiosensitizes PDAC cells, in part, by affecting G2/M arrest and inducing apoptosis via FDPS inhibition.

### Zol enhances radioresponse by suppressing PDAC xenograft growth and metastasis

**Figure 3:**
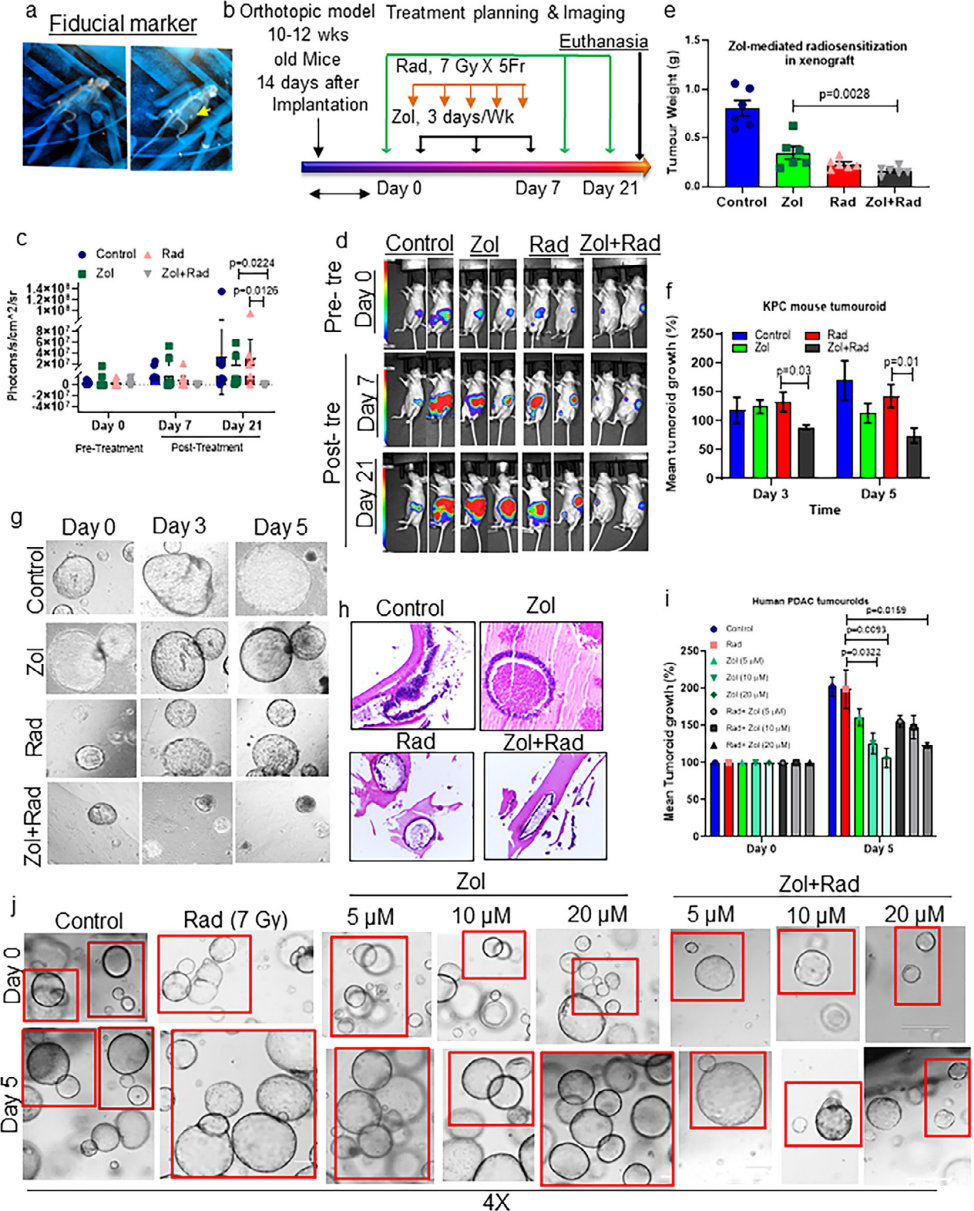
Radiosensitization efficacy of Zol in PDAC xenograft, *in vitro* 3D murine KPC tumouroids, and human PDAC tumouroid models. (a) Representative MRI imaging showing implantation of fiducial marker along with xenograft PDAC cells in athymic nude mice. (b) Schema showing treatment schedule of Zol and RT. (c and d) Mice were (i.p.) injected with luciferin and imaged using an IVIS at 0, 7, and 21 days of Zol and RT treatments. (c) Dot plot showing tumour volume/response measurement as total photon flux at 21 days post-treatment in each group (Zol *vs.* Zol+Rad (*P* = 0.0224), Rad *vs.* Zol+Rad (*P* = 0.0126), n=6, ANOVA, Tukey's multiple comparison test). (d) Representative images show bioluminescent images of mice in all groups. (e) The bar graph shows the quantification of xenograft tumour weights in grams (Zol *vs.* Zol+Rad (*P* = 0.0028), n=6, ANOVA, Tukey's multiple comparison test). (f) The bar graph shows the mean growth of KPC tumouroids exposed to no drug, Zol, and/or RT alone. Tumouroid growth (µM) was measured using software associated with Zeiss Live imaging system. Tumouroid size at day 3 (Rad *vs* Zol+Rad (*P* = 0.03)), Student's *t-test*) and day 5 (Rad *vs* Zol+Rad (*P* = 0.01)), *n* = 6, student's *t-test*) was normalized to day 0. (g) Representative light microscope images of KPC tumouroids were taken at days 0, 3, and 5 following drug treatment. (h) Histological examination revealed more viable tumour cells in control (no drug treatment) tumouroids compared with tumouroids treated with RT and Zol alone and in combination. (i) Bar graph showing average growth/response of human PDAC tumouroids after drug treatment. Tumouroid size at Day 5 (Rad *vs* Zol (10 µM) (*P* = 0.0322), Rad *vs* Zol (20 µM) (*P* = 0.0093), Rad *vs* Zol (20 µM) + Rad (*P* = 0.0159), n=6, student's *t-test*) were normalized to Day 0 of respective tumouroids. (j) Representative images depict the response of human PDAC patient-derived tumouroids to various doses of Zol alone and in combination with RT.

We utilized the mouse pancreatic orthotopic tumour model to identify the molecular mechanism(s) of Zol-mediated radiosensitization *in vivo*. First, we performed a biodistribution study using radiolabeled Zol (3H-Zol) in non-tumour-bearing mice to evaluate whether Zol reaches the pancreas. Comparative biodistribution of a radiolabeled probe into different organs with time kinetics shows that Zol accumulated in the pancreas 4 hrs after tail vein injection and remained in pancreas for up to 120 h (**Figure S8a**). Next, we implanted CD18/HPAF cells in the mouse pancreas and a platinum-based fiducial marker to precisely locate the xenograft tumour using CT to better improvise the treatment planning (Figure 3**a and b**). We found that CD18/HPAF xenografts treated with RT and i.p injected Zol (16.4 µg/mouse, mouse equivalent dose calculated based on previous clinical trials) exhibited significantly greater RT response and reduced xenograft tumour weight and metastasis as compared to Zol or RT alone and control groups (Figures. 3**c–e and S8b**). A linear mixed model analysed the photons measured using IVIS imaging at days 0, 7, and 21. There was a significant interaction between the treatment group and days, indicating the treatment effect differed by day (*P* = 0.0064) (**Tables S4, S5, and S6**). The overall pairwise comparison between treatments groups is significant at Day 21 (Rad vs. Zol+Rad (*P* = 0.0126), Zol vs. Zol+Rad (*P* = 0.0224)) (**Table S6**). Xenograft tumour weights in the four groups were found to be significantly different based on ANOVA. Pairwise comparison between Zol and Zol+Rad treated xenograft tumour weight was significant (*P* = 0.0028) (**Tables S7 and S8**). Pancreatic tumour xenografts and major vital organs (liver and spleen) were subjected to histological analysis to examine tumour necrosis and acute or chronic toxicity (**Figure S8c and d**). Zol and RT alone modestly reduced the survival of PDAC cells with increased necrosis in the RT alone group. There was no sign of drug- or RT-related toxicities in the liver and spleen in any of the treatment groups. These observations were further validated by evaluating the expression of Ki67 (proliferative marker) and cleaved caspase 3 (apoptosis marker) in xenograft tissues. Our IHC staining for Ki67 and cleaved caspase 3 antibody revealed that Zol alone and in combination with radiation-exposed xenograft tissues showed significantly decreased Ki67 nuclear staining and increased positive cytoplasmic expression of cleaved caspase-3 as compared with control and radiation alone treated xenografts (**Figure S9a–d**). These results are the consequence and the signs of necrosis and the histopathological difference between treatment groups, as observed in **Figure S8c and d**).

### Zol treatment radiosensitizes 3D murine and human PDAC tumouroids

To determine the radiosensitization efficacy of Zol in 3D *in vitro* models of PDAC, we utilized recently developed murine KPC and human PDAC tumouroid models. We found that pre-treatment of tumouroids with Zol followed by RT significantly reduced growth of mouse (*P* = 0.03, *P* = 0.01) and human (*P* <0.01, *P* <0.05) tumouroids at days 3 and 5 post-IR+ Zol treatments (Figure 3**f–j**). Thus, our results in tumouroid and xenograft models suggested that Zol radiosensitizes both human and mouse PDAC.

### Transcriptomic analysis reveals Zol combined with RT induces DNA damage and repair gene signature through Rac1

**Figure 4:**
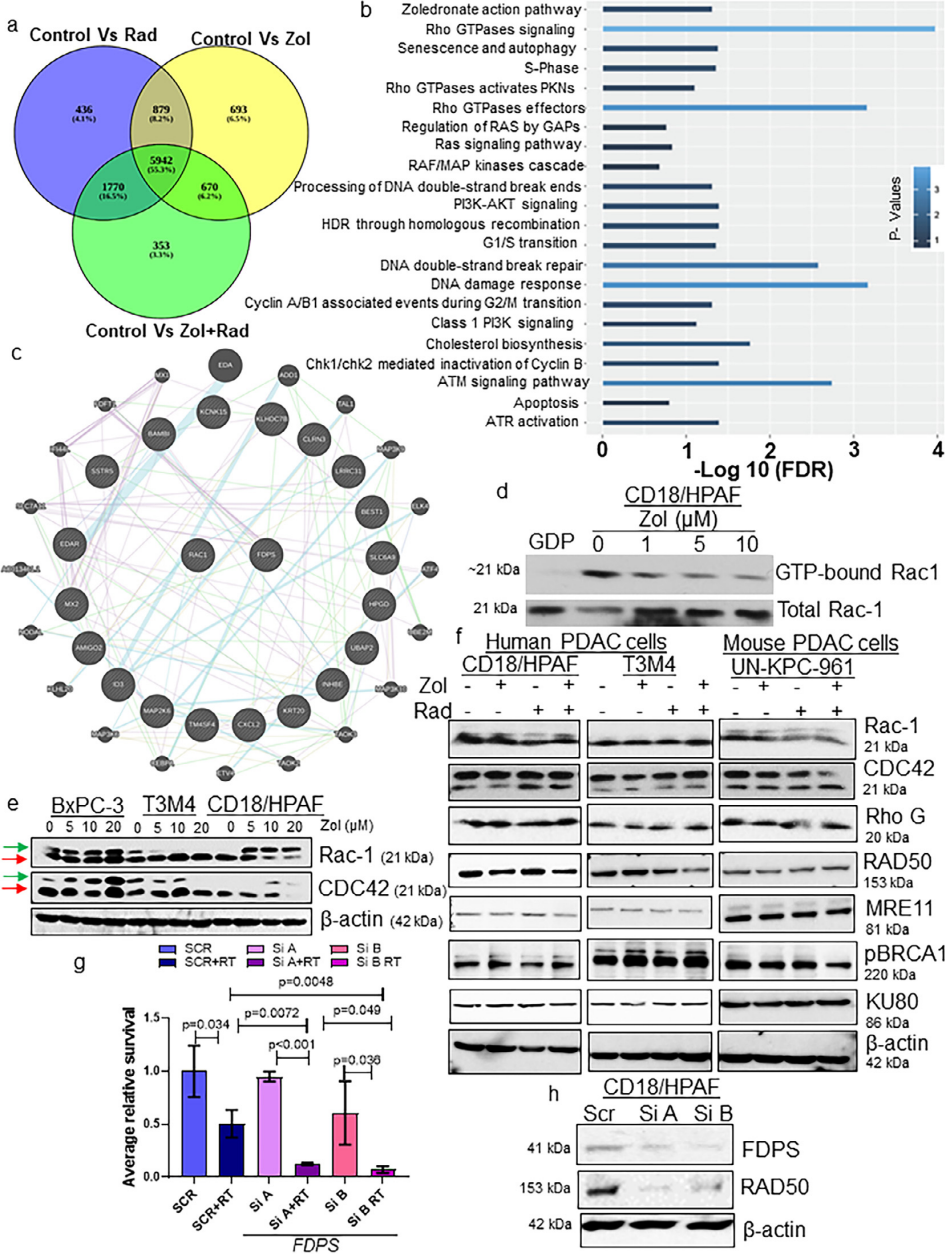
Zol and RT treatment modulate DNA damage response by deactivating Rho small GTPases, thereby reducing DNA double-stranded break repair proteins. (a) Venn diagram showing specific differentially expressed genes in different treatment groups. (b) Pathway enrichment analysis of common and unique genes in the RT versus Zol+RT group. (c) Integrated network analysis of differentially expressed genes in the Zol+RT versus RT group using STRING version 10.5. (d) Dose-dependent treatment of PDAC cells with Zol decreased Rac-1 activity. (e) Western blot analysis confirmed the expression of prenylated (Arrows in red) and unprenylated (Arrows in green) forms of Ras family members Rac-1 and CDC42 upon dose-dependent treatment of Zol in BxPC3, T3M4 and CD18/HPAF PDAC cells. (f) Human and mouse PDAC cells were exposed to Zol (5 µM) 4 h prior to RT, lysates were prepared, and proteins were separated using SDS-PAGE and analysed for antibodies specific for small GTPases (Rac-1, CDC42, and Rho G) and DNA repair and damage signalling (RAD50, MRE11, pBRCA1, and KU80) (g and h) Transient knockdown (KD) of FDPS in PDAC cells suppressed FDPS and subsequently reduced RAD50. (g) Clonogenic survival assay confirmed that FDPS suppression reduced the number of colonies as quantified using Image J software. The bar graph represents the results of the mean ±SE of colonies that survived after FDPS KD and RT (SCR *vs.* SCR+RT (*P* = 0.034), Si-A *vs.* Si-A+RT (*P* < 0.001), Si-B *vs.* Si-B+RT (*P* = 0.036), SCR+RT *vs.* Si-A+RT (*P* = 0.0072), Si-A+RT *vs.* Si-B+RT (*P* = 0.049), SCR+RT *vs.* Si-B+RT (*P* = 0.0048), n=3, student's *t-test*). (h) FDPS transient transfection potency was verified using lysates prepared from transfection of non-targeting (Scramble) and siRNA targeting FDPS in PDAC cells and analysed for FDPS, RAD50, and β-actin protein by western blot analysis.

DNA damage and radioresponse signals are short-lived. Therefore, to assess the effects of Zol post-radiation, we used a short-term *in vivo* treatment model. As expected, Zol treatment of irradiated xenograft tumours significantly reduced tumour size and decreased xenograft growth (Rad vs. Zol (*P* = 0.0161), Zol vs. Zol+Rad (*P =* 0.0029), **Tables S9, S10, and S11**), as evidenced by the decrease in photons in the IVIS images on day 7 relative to Zol and RT alone at Day 0 (randomization) (**Figure S10a, b, Tables S9, S10, and S11**). We excised xenograft tissues immediately after the last radiation and performed global transcriptome analysis on total RNA isolated from short-term-treated xenograft tissues (CD18/HPAF) exposed to Zol and/or RT. All sequenced and mapped genes were screened between untreated control and treated (Zol/RT alone and in combination) cells. A total of 5942 common and 353 differentially expressed genes (DEGs) (above and below the log_2_fold change of 2, *P* <0.05, FDR <0.01) were observed between groups (Figure 4**a**). Among the DEGs, TM4SF4, UNC5B, PDK4, ID1 (related to mevalonate pathway), UBAP2, PLK2, LPCAT1, LAMA3, FSCN1, and BHLHE40 were differentially (top 5 DEGs in bold) regulated in response to Zol-mediated radiosensitization in PDAC cells (**Figure S10c**). Our gene enrichment and pathway analysis through KEGG revealed that several critical pathways such as (a) Zoledronate action pathway, (b) Rho GTPases, (c) Ras, (d) RAF/MAP kinases, (e) DNA double-strand break, (f) PI3K-AKT, (g) G2/M transition, (h) DNA damage response, (i) ATM and (j) ATR signalling pathways were significantly enriched in cells treated with Zol and RT (Figure 4**b**). Interactome analysis revealed a significant association of Rac1 with Zol activity, which was previously shown to be associated with RR in PDAC (Figure 4**c**).^27^, ^28^, ^29^

We exposed PDAC cells to various Zol concentrations (0, 1, 5, and 10 µM) and performed a Rac1 pulldown assay to assess its dose-dependent inhibition of Rac1 activity. Exposure of PDAC cells to Zol reduced GTP-bound Rac1 (Figure 4**d**). Lysate pulldown with GDP (guanosine5′-diphosphate) served as a negative control. Similarly, Zol treatment alone increased the un-prenylated form of both Rac1 and CDC42 compared to untreated control PDAC cells (Figure 4**e**). Interestingly, we also tested whether the ectopic expression of constitutively active Rac1 (Rac1^G12V^ mutant) in PDAC cells could reverse the *in vitro* Zol radiosensitizing effects. Despite constitutive active Rac1, pre-treatment with Zol alone and in combination with RT showed consistent significant radiosensitizing impact similar to observations in other human and mouse PDAC cells (**Figure S11a and b**), indicating that Zol exhibits global disruption of Rac1 activity irrespective of its mutant status. In addition, Zol treatment in combination with RT resulted in synergistic control and an increase in the un-prenylated forms of Rac1, CDC42, and Rho G, which correlated directly with relative FDPS inhibition in PDAC cells human and mouse PDAC (CD18/HPAF, T3M3, and UN-KPC-961) cells (Figure 4**f**). This suggests that Zol-mediated FDPS inhibition regulates prenylation of small GTPases, which could be a mechanism controlling the DNA damage signals that emerge immediately after RT. We also observed that DNA damage-response MRN (MRE11-RAD50-NBS1) complex and DNA repair-associated tumour suppressor protein pBRCA1 were reduced upon Zol and RT in PDAC cells (Figs. 4**f and S10d**) with no changes in Ku80 double-stranded DNA break repair protein, indicating that inhibition of these DNA repair pathways is the reflection of sustained radiosensitization effects of Zol (Figure 4**f**). Notably, IHC analysis for DNA damage response proteins (RAD50 and pP95 (NBS1)) in xenograft tissues indicated significantly decreased expression for analysed proteins (RAD50 (*P* < 0.001) and pP95 (*P* < 0.001) in the Zol+RT treatment group relative to radiation alone confirming the retention of combination effect *in vivo* (**Figure S12a-d**). In addition, several genes related to Rho and Rac1 signalling were downregulated in response to Zol+RT relative to RT alone (**Figure S13a and b**). Further, transcriptomic analysis on xenograft tissues treated with Zol and RT demonstrated downregulation of genes significantly associated with the Rho family of GTPases. Among Rho subfamily genes, Rac1 and Rac2 are significantly downregulated compared to other genes of the same subfamily (**Figure S14**). In addition, depletion of FDPS in PDAC cells reduced the mean number of survival colonies by downregulating Rad50 protein levels, indicating Rad50 is a direct target of FDPS **(**Figure 4**g and h)**. Overall, inhibition of FDPS by Zol reduces protein prenylation and small GTPase activity; thereby, it further impairs DNA damage, response, and repair signalling resulting in enhanced radiosensitivity of PDAC cells in both *in vitro* and *in vivo* (Figure 4**i)**.

### Evaluating the safety and efficacy of Zol combined with SBRT/5FU or capecitabine in the clinical trial

In our ongoing Phase I/II clinical trial evaluating the safety and efficacy of ZOL in radiosensitizing PDAC tumours in borderline resectable patients (NCT03073785), PDAC patients were randomized to either ARM A (RT/5FU or capecitabine) or ARM B (RT/5FU or capecitabine + Zol). Patient randomization, intervention, and follow-up are demonstrated in **Figure S15**. The trial has passed first (safety) and second (first stage of the two-stage design) interim analyses and has entered the second stage. To date, 45% (5/11) of patients who received Zol+RT and 33% (5/15) who did not receive Zol (RT alone) became surgical candidates and underwent surgical resection. One patient in the Zol arm had a complete pathologic response.

### Zol enhances the radioresponse of human PDAC tumours by exerting systemic immunomodulatory effects

**Figure 5:**
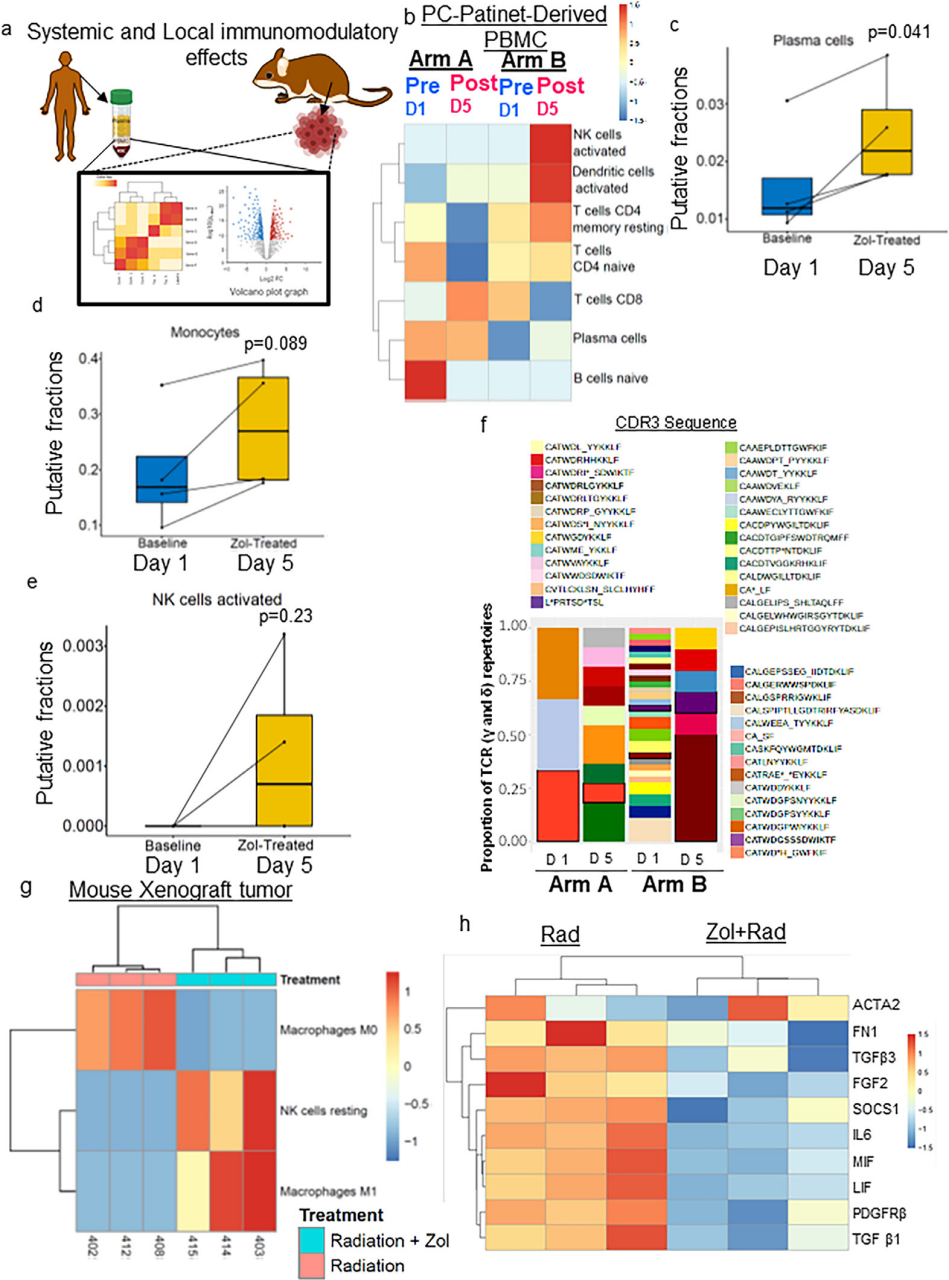
Pre-treatment with Zol induces immunomodulatory effects in conjunction with neoadjuvant RT and capecitabine in borderline resectable PDAC patients. (a) Cartoon demonstrating the systemic and local immunomodulatory effects of Zol identified from the PDAC patientPBMCs and xenograft tissues. (b) Heatmap representing immune fractions between Day 1 and Day 5 of patients in Arm A and Arm B. Scale- fold change fractions of immune cell types in peripheral blood. (c-e) Box plot showing an enhanced response of immune components (plasma cells (*P* = 0.041), monocytes (*P* = 0.089), and activated NK cells (*P* = 0.23), Pairwise *t-test*) in Arm B patients treated with RT+ZOL at Day 5 (n=4) relative to Day 1 (n=4). (f) Stacked bar plot showing the expansion and proportion of TCRγ and TCRδ repertoire in PBMCs isolated from RT alone and RT+Zol treated PDAC patients enrolled in ongoing Phase I/II clinical trial. (g) Heatmap of tumour microenvironment-related gene sets significantly impacted upon Zol and RT treatment versus RT alone in xenograft model tissues with *P* <0.05 and FDR <25%. (h) Heatmap of significantly downregulated genes related to cancer-associated fibroblast when comparing Zol+RT treated xenograft tumours versus RT alone.

Next, we focused on the correlation of Zol treatment with immunomodulatory effects. Hence, we performed CIBERSORT analysis on the transcriptomic data obtained through Human PBMCs and xenograft tissues (Figure 5**a**). First, the surgically resected PDAC tissues were pathologically evaluated for their clinical response to Zol+RT treatment. Based on the pathological score (0 means no response and 9 is the complete response), we selected blood samples and extracted blood samples from a single patient from Arm A and B. Then, we determined the gene expression levels of immune response and the tumour microenvironment (TME) factors. As expected, PDAC patients in Arm B treated with Zol+RT had significantly higher putative fractions (CIBERSORT) of activated NK cells, activated dendritic cells, CD4^+^ T memory resting, CD4^+^ T cell naïve, and plasma cells relative to Arm A patient treated with RT alone, but there was no difference in the proportion of naïve B cells (Figure 5**b**). Specifically, we found significantly higher proportions of plasma cells (N=4) and differences in monocytes (N=4) and activated NK cells (N=4) on day 5 as compared with day 1 of Arm B patients (Figure 5**c-e**). We also found that the proportion of variants of γδ T cells are expanded upon Zol treatment in PDAC patients’ peripheral blood. Among the subpopulation of γδ T cells (δ1, δ2, δ3) Vγ9Vδ2 is predominantly (90%) present in peripheral blood.^30^ Hence, we analysed the proportion of T cell receptor (TCR) γ and δ repertoire in the RNA seq data obtained from Day1 and Day 5 of RT+Zol treatment. We found that several variants and expansions of TCR γ and δ repertoire were high on Day 5 compared to Day 1 of the same patient (Figure 5**f**). This is mainly due to the accumulation of isopentenyl pyrophosphate (IPP) as a result of FDPS inhibition by Zol in the tumour cells resulting in stimulation and activation of γδT cells (Vγ9Vδ2).

**Figure 6:**
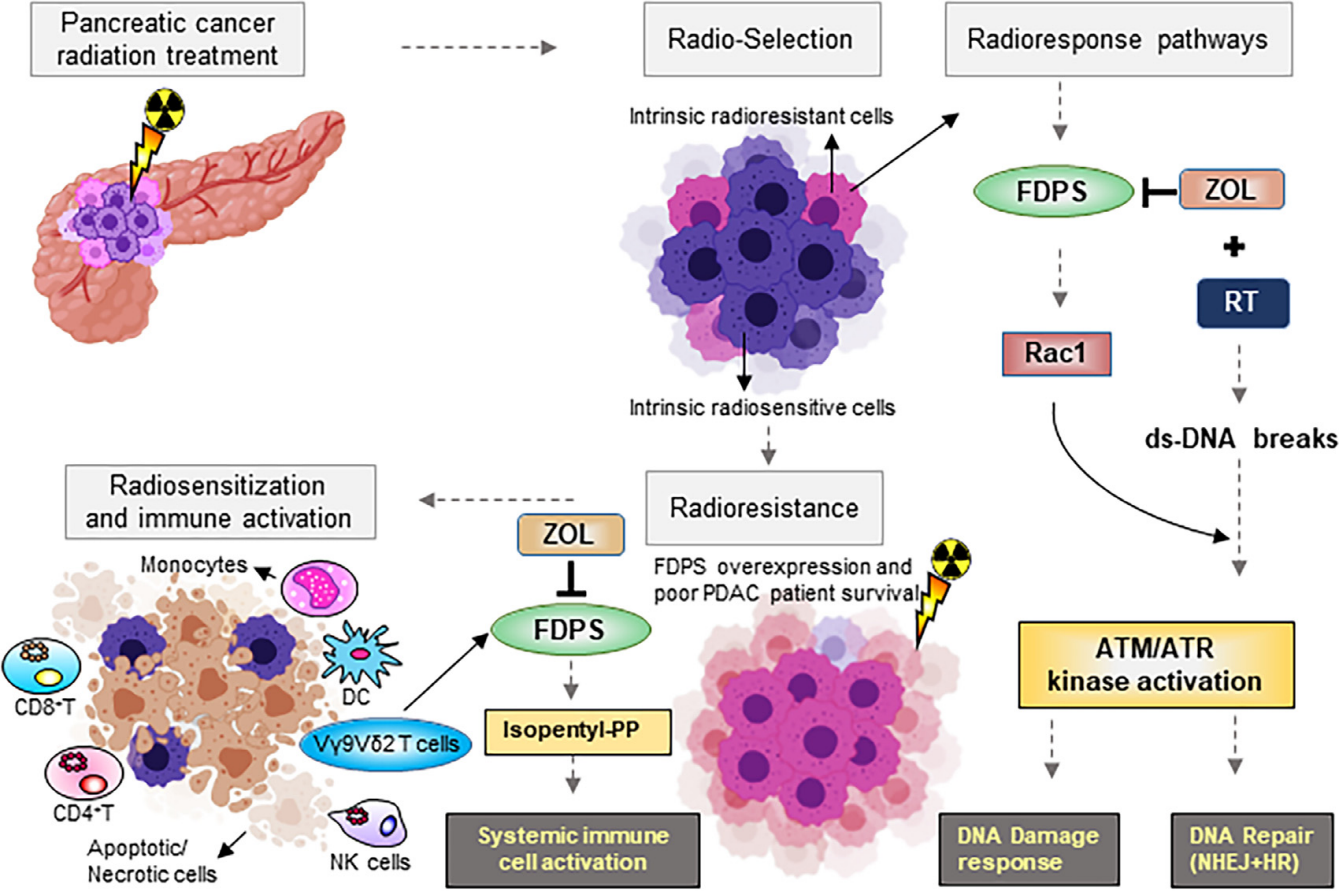
Cartoon demonstrating Zol-mediated inhibition of FDPS enhancing radiosensitization and potentially exerting immunomodulatory effects by modulating various immune cell types and altering tumour immunity. In PDAC xenograft cells, Zol inhibition of FDPS exerts local tumour control by reducing prenylation of small GTPases, thereby affecting multiple PDAC-promoting pathways such as survival and proliferative signalling (AKT and ERK), DNA damage, and DNA repair mechanisms resulting in promoting G2M arrest and apoptosis. Furthermore, as we observed from PDAC patient's PDMCs, Zol disruption of FDPS leads to accumulation of isopentenyl pyrophosphate (IPP) which activates gamma-delta T cells. Zol reverses the M2 to M1 macrophage phenotype, and activates NK cells. Similarly, in dendritic cells and NK cells co-culture studies, ZOL reduced the endogenous phenyl pyrophosphate present in antigen-presenting dendritic cells, leading to IL-1β and IL-18 secretion, which activates NK cells to secrete IFN-γ. Apart from these effects on immune components, Zol could exhibit systemic immunomodulatory effects on CD4^+^ and CD8^+^ T cells.

Further, we validated these immune cell signatures in the RNA sequence analysis from xenograft tissues by genes mapping aligned for mouse sequence and processed with CIBERSORT and Kallisto analysis. As shown in the heatmap, the proportions of genes associated with Macrophage M0 are decreased, whereas putative fractions representing M1 Macrophages and NK cells resting are increased in Zol+RT treated mice compared with radiation alone treated xenografts (Figure 5**g**). Additionally, the fibroblast activation-associated gene signatures were downregulated in Zol and RT-treated xenograft tumours (Figure 5**h**). These downregulated TME-associated gene signatures were further validated by RT-qPCR analysis in the PDAC cells and xenograft tissues treated with and without Zol and RT (**Figure S10e**). Our RT-qPCR results confirmed a significant downregulation of *TGFβ3, FGF2, MIF, PDGFRβ*, and *IL6* in Zol+RT treated xenograft tissues relative to RT alone treatment. Finally, PDAC cells exposed to Zol+RT also showed significant downregulation of *IL6* compared to isogenic cells treated with radiation alone. These data suggest that Zol treatment have a significant impact on immune cell activation and negative regulation of immunosuppressive cells. Thus, Zol may be beneficial in enhancing the efficacy of immunotherapy in the treatment of PDAC. Altogether, these preclinical and clinical data suggest that the therapeutic effect of Zol on PDAC cells involves inhibition of PDAC growth (Figure 6).

## Discussion

RT effect is minimal in PDAC patients due to intrinsic and acquired resistance ^5^. This study sought to identify and characterize pathways contributing to RR in PDAC that can be explored as novel targets for radiosensitization. Recent studies have highlighted FDPS as a critical determinant of cancer progression and survival in acute myeloid leukemia, melanoma, sarcoma, and PDAC patients.^7^,^31^ In addition, FDPS is a critical gene involved in CBS and is required for the isoprenylation of Rho GTPase Rac1, which drives K-Ras-driven cancers.^29^,^32^, ^33^, ^34^ Unbiased pathway analysis indicated that FDPS to be differentially expressed and associated with the RR phenotype ^7^, but the mechanism by which FDPS regulates response to radiation and the therapeutic potential of targeting FDPS to overcome RR was not been explored.

In this study, we investigated the role of FDPS in PDAC RR. This was clinically supported by the increased expression of FDPS along with Ki67 in the tissue of PDAC patients treated with neoadjuvant hypofractionated stereotactic RT. Furthermore, we demonstrated that significantly higher expressions of FDPS and Ki67 were associated with RT refractoriness and with poor survival. This was similar to the observation of Kim *et al*. ^10^, who demonstrated that FDPS was highly expressed in aggressive glioma and associated with poor survival. Our transcriptomic analysis of PDAC tissues is the first report of FDPS as a biomarker for RT response. Furthermore, through *in silico* analysis, we show that PDAC patients have significant overexpression of *FDPS* compared to the normal pancreas. FDPS deregulation has also been reported in glioblastoma, colon, and prostate cancer progression.^8^, ^9^, ^10^, ^11^ When a cancer cell is irradiated, the stress response signal activates the sterol regulatory element‑binding protein (SREBP). When SREBP enters the nucleus, it binds to the sterol response element present in the FDPS promoter ^35^. This research points towards the likelihood that sustained SREBP mediates FDPS overexpression in response to the radiation treatment.^36^ Our data show that chronic RT of PDAC cells results in upregulation of FDPS, cyclin D1, cyclin B1, and cancer stem cell markers in PDAC cells accompanied by altered cellular morphology, increased survival facilitated by G2/M arrest, and an increased side population.

Furthermore, depletion of FDPS led to greater sensitivity to RT than control cells with a marked reduction in phosphorylated AKT, ERK, and DNA repair protein, RAD50. This is in agreement with our previous work and others, showing that FDPS modulates phosphorylation of AKT and ERK.^8^, ^9^, ^10^, ^11^ These data also provided a rationale to target FDPS and prompted us to test the utility of Zol, a specific inhibitor of FDPS,^37^,^38^ as a radiosensitizer for PDAC. Indeed, Zol administration before RT has been shown to prevent mesenchymal stem cell loss.^39^ Furthermore, treatment with Zol in conjunction with RT resulted in reduced colony growth, enhanced apoptosis, and retracted radiation-associated G2/M cell cycle arrest. Zol demonstrated similar anticancer effects in osteosarcoma, esophageal, and breast cancers related to skeletal or bone metastasis with a low radiation dose.^40^, ^41^, ^42^

Our orthotopic model is the first *in vivo* approach to mimic clinical radiosensitization, which differs from most conventional superficial xenograft models.^43^,^44^ This clinically relevant model allowed us to deliver higher doses of RT to tumours while minimizing the chance of RT exposure and radiation-induced toxicity to uninvolved vital organs located closer to the pancreas, such as the liver, intestine, and kidney.^45^ The *in vivo* radiosensitization studies showed that xenograft tumours treated with RT and i.p. injected with Zol (16.4 µg/mouse, mouse equivalent to clinical dose) ^46^ had a significantly greater response, higher tumour shrinkage, and lower metastasis incidence as compared to Zol or RT alone and control groups. Furthermore, upon histological analysis of vital organs such as the spleen and liver located close to the pancreas, no signs of drug or radiation-related acute and chronic toxicities were observed.

Similar to *in vivo* radiosensitization with Zol, we also observed significant retardation of KPC and human PDAC patient-derived tumouroids growth with Zol+RT exposure. These findings in the 3D models will useful in accurately predicting the pharmacological impact of Zol on the heterogeneous human pancreatic tumour microenvironment, which is a major challenge in PDAC.^47^

Through our global transcriptomic analysis followed by *in silico* analysis on the pancreatic xenograft tissues, we were able to identify modulation of PI3K, Ras, Rho, and DNA damage response signalling as mediators of Zol-induced radiosensitization. Further, an interactome analysis of the top differentially expressed genes in radiation- and Zol-treated groups revealed Rac1 as one of the mediators of the FDPS effect. Previously, Rac1 was shown to be overexpressed and constitutively activated, and its activity was unaffected by anticancer agents.^28^,^29^,^33^,^34^. However, we found that Rac1, associated with PDAC RR, was downregulated and among the network genes affected with Zol+Rad treatment. Moreover, inhibition of prenylation of Rac1 and CDC42 small GTPases, along with the decreased accumulation of intracellular cholesterol, served as useful readouts of the efficiency of Zol in inhibiting FDPS activity. In fact, inhibition of prenylation is effective against Ras-driven and Rac1-hyperactivated tumours.^34^ In addition, Zol treatment suppressed Rac-1 activity in a dose-dependent manner. Thus, both genetic and pharmacological inhibition of FDPS enhanced radiosensitivity by impairing DNA damage response genes such as ATM, pP95, and Rad50 through upstream Rac1 activation. These studies are substantially supported by the work of Varela *et al*., which demonstrated that direct inhibition of ATM signalling by Zol and prevented DNA damage signalling.^48^ Through our study, we elucidated the direct link between FDPS-Rac1-ATM signalling as a possible mechanism of RR.

Supportively, Zol is also under investigation as a combination agent with tyrosine kinase inhibitors in non-small cell lung cancer (NSCLC) (NCT03958565), as a surgical adjuvant in giant cell cancer of bone (NCT03295981), and in a study that is evaluating safety and tolerability of Zol before and after surgery in patients with grade I-III chondrosarcoma (NCT03173976). Our interim results show that concurrent Zol and SBRT is a safe and feasible approach in this cohort. The resection rate after neoadjuvant therapy was 40% and 33% with and without Zol, respectively. In addition to its direct anti-tumour effects, Zol has been shown to influence macrophage polarization, γδ T cell expansion, enhancement of natural killer (NK) cell activity, Tregs activation and infiltration, modulation of PD-L1 expression, and T helper and cytotoxic cell functions.^12^,^49^ In addition, Zol treatment also expansion of human Vγ9/Vδ2 T cells and TCR-dependent phosphorylated metabolites to cytolyze cancer cells by secreting chemokines and cytokines. Activated γδ T exert cytolytic effects against tumour cells or APCs in an MHC-independent manner.^30^ In a recent study, Merli *et al*. demonstrated that Zol infusion after administration of TCRαβ/CD19 depleted cells enriched with the mature natural killer and TCRγδ T cells isolated from haploidentical hematopoietic stem cell transplantation (HSCT) donor (haplo-HSCT) accelerated TCRγδ T cells differentiation and expansion. By this approach, it has been shown that Zol infusion after transplantation promoted immune reconstitution and reduced disease recurrence and risk of post-infection-related complications in pediatric acute leukemia patients.^50^ Several clinical studies such as Austrian Breast and Colorectal Cancer Study Group trial 12 (ABCSG-12), Zometa-Femara Adjuvant Synergy Trial (ZO-FAST), and Adjuvant Zoledronic acid to reduce recurrence (AZURE) studies demonstrated modulation of immune-suppressing regulatory T (Treg) cells by inhibiting its proliferative capacity via downregulating CCR4, PD-1, CTLA-4 and cells the immunomodulatory effects of Zol by receptor activator of nuclear factor kappa-B ligand (RANKL) on Treg cells.^51^ Furthermore, a seminal study by Weber and colleagues demonstrated that Zol alone and in combination with surgical trauma significantly increased infiltration and shifted systemic macrophage polarization toward M1 type in spleen, lung, and skin of Wister rat model.^52^ All these studies provide additional evidence of Zol contributing towards modulation of the immune system. PDAC is very difficult to treat and resistant to most conventional therapies due to dense stromal meshwork and immunosuppressive microenvironment.^53^ To study Zols immunosuppressive and immunomodulatory effects, we performed CYBERSORT analysis on the RNA-seq data obtained from PDAC patient-derived PBMCs and xenograft tissues. Our results demonstrated that Zol exerts immunomodulatory effects by modulating various immune cell types, specifically increasing plasma cells (Figure 6). In the future, the immunomodulatory effects of Zol can be exploited in conjunction with immune checkpoint inhibitors to improve the efficacy of RT for PDAC patients. The ongoing Phase I/II study of SBRT with or without Zol will evaluate the safety and efficacy of Zol+RT/5FU or capecitabine. Enrollment is ongoing, and results are anticipated in 2022.

In summary, our studies demonstrate that FDPS is a viable target to reduce PDAC RR. One of the major innovations of our study is the development of an image-guided animal model for preclinical testing of radiosensitizers for PDAC. Major caveats and limitations related to the main study includes: (a) prognostic markers that predict Zol's response in PDAC patients were not identified yet, (b) small sample size/population enrolled in our clinical trial limits the ability to relate the association of Zol+RT response and overall and disease-free survival, (c) the clinical follow-up is short, and the trial has not completed yet with only interim results, and (d) *in vivo* studies needs to be validated in spontaneous KPC mouse models. In the future, we plan to apply this approach to PDAC patients harboring oncogenic KRAS in association with additional alterations, i.e., p53, CDKN, and SMAD4 mutations/deletions, etc., to predict theefficacy of Zol on a specific patient population or subgroup of PDAC patients. Our work demonstrated the safety and efficacy of Zol+RT in a single institution randomized phase I/II clinical study.^54^ Our work may serve as a stepping stone in establishing an optimal multimodality neoadjuvant PDAC regimen by incorporating combination chemotherapy, immunotherapy, and RT with Zol.^4^,^55^,^56^ Our study also provides a rationale for applying a similar radiosensitization approach in other cancers with CBS pathway dysregulation.

## Declaration of interests

Dr. Surinder K. Batra is one of the co-founders of Sanguine Diagnostics and Therapeutics, Inc. All other authors disclosed no potential conflicts of interest.

## Appendix Supplementary materials

Image, application 1

Image, application 2

Image, application 3
