# Supplementary material for: Disruption of FDPS/Rac1 axis radiosensitizes pancreatic ductal adenocarcinoma by attenuating DNA damage response and immunosuppressive signalling
Source: eBioMedicine. 2021 Dec 28;75:103772. doi: 10.1016/j.ebiom.2021.103772 (PMC8718746; doi:10.1016/j.ebiom.2021.103772)
Supplement: Supplementary file 1 [file mmc1.pptx]

## Slide 1
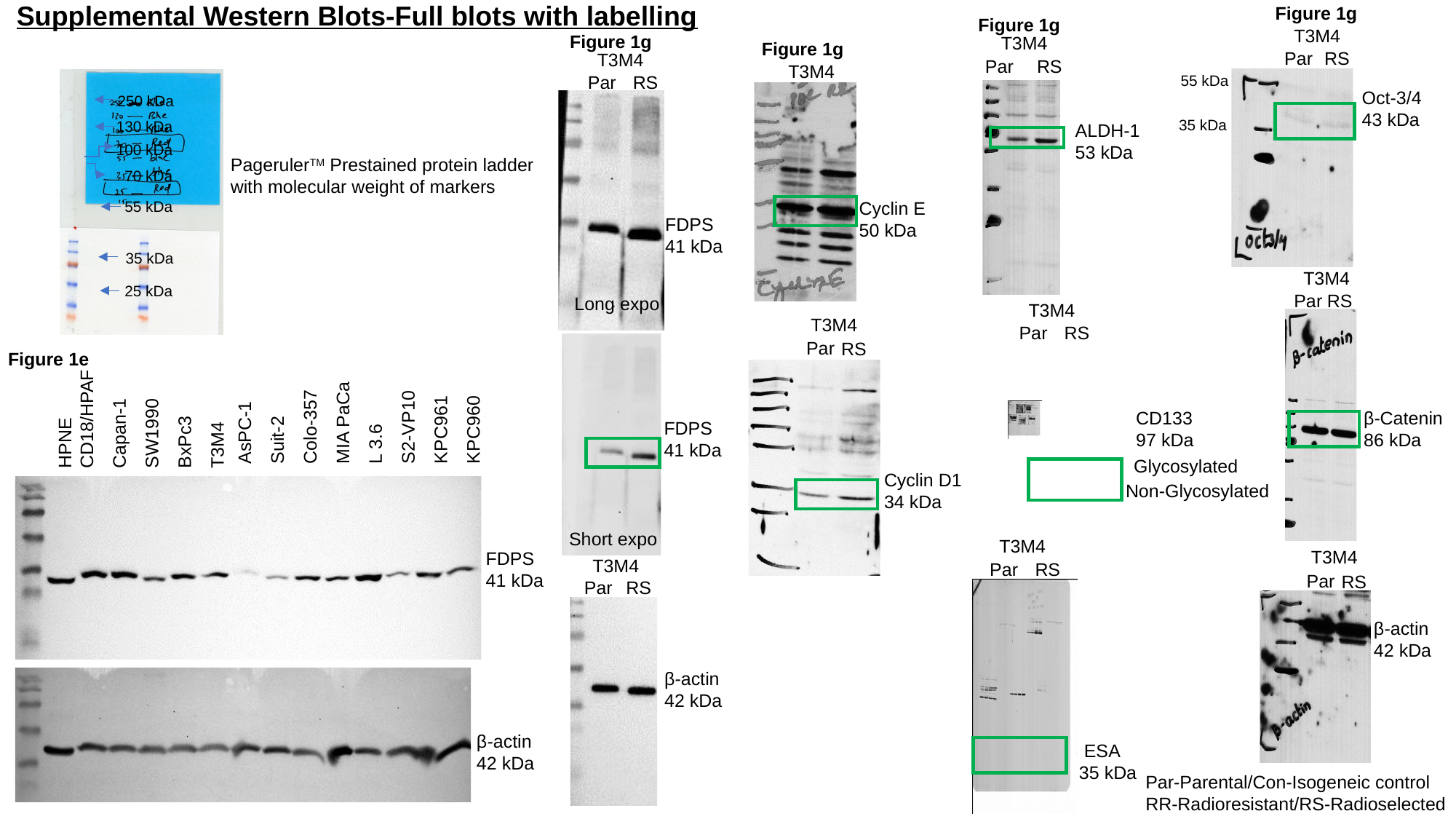

Supplemental Western Blots-Full blots with labelling
Figure 1g
Figure 1g
T3M4
Par
RS
 55 kDa
 35 kDa
Figure 1g
T3M4
Par
RS
ALDH-1
53 kDa
Figure 1g
T3M4
T3M4
Par
RS
 250 kDa
 130 kDa
 100 kDa
PagerulerTM Prestained protein ladder
with molecular weight of markers
 70 kDa
 55 kDa
 35 kDa
 25 kDa
Oct-3/4
43 kDa
Cyclin E
50 kDa
FDPS
41 kDa
T3M4
Par
RS
β-Catenin
86 kDa
Long expo
T3M4
Par
RS
CD133
97 kDa
 Glycosylated
Non-Glycosylated
T3M4
Par
RS
Cyclin D1
34 kDa
AsPC-1
Suit-2
Colo-357
MIA PaCa
L 3.6
S2-VP10
KPC961
KPC960
HPNE
CD18/HPAF
Capan-1
SW1990
BxPc3
T3M4
Figure 1e
FDPS
41 kDa
β-actin
42 kDa
FDPS
41 kDa
Short expo
T3M4
Par
RS
 ESA
35 kDa
T3M4
Par
RS
β-actin
42 kDa
T3M4
Par
RS
β-actin
42 kDa
Par-Parental/Con-Isogeneic control
RR-Radioresistant/RS-Radioselected

## Slide 2
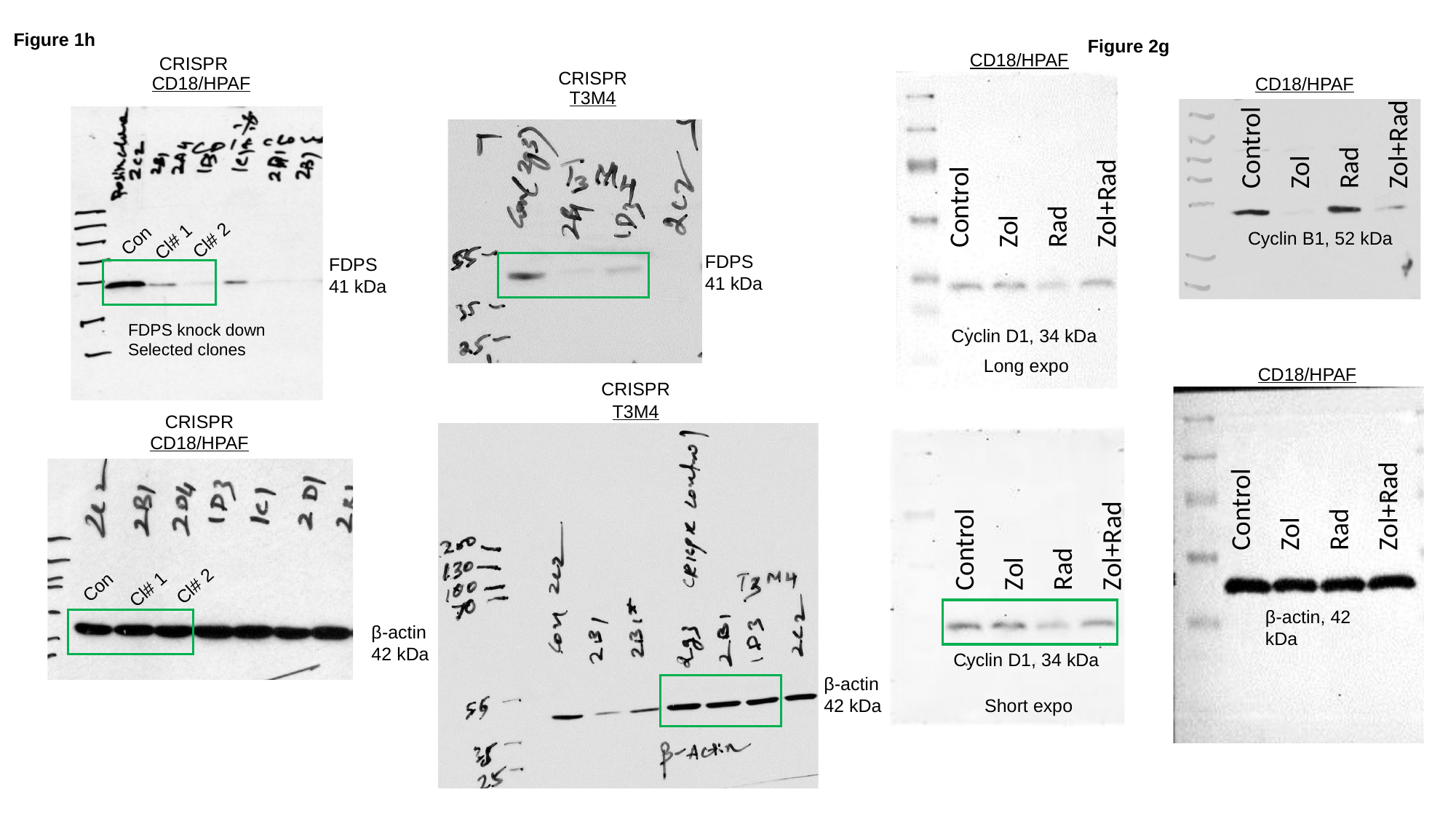

Figure 1h
Figure 2g
CD18/HPAF
CRISPR
CRISPR
CD18/HPAF
CD18/HPAF
T3M4
Control
Zol
Rad
Zol+Rad
Cyclin B1, 52 kDa
Control
Zol
Rad
Zol+Rad
Con
Cl# 2
Cl# 1
FDPS
41 kDa
FDPS
41 kDa
FDPS knock down
Selected clones
Cyclin D1, 34 kDa
Long expo
CD18/HPAF
CRISPR
Control
Zol
Rad
Zol+Rad
T3M4
CRISPR
CD18/HPAF
Control
Zol
Rad
Zol+Rad
Cl# 2
Con
Cl# 1
β-actin, 42 kDa
β-actin
42 kDa
Cyclin D1, 34 kDa
β-actin
42 kDa
Short expo

## Slide 3
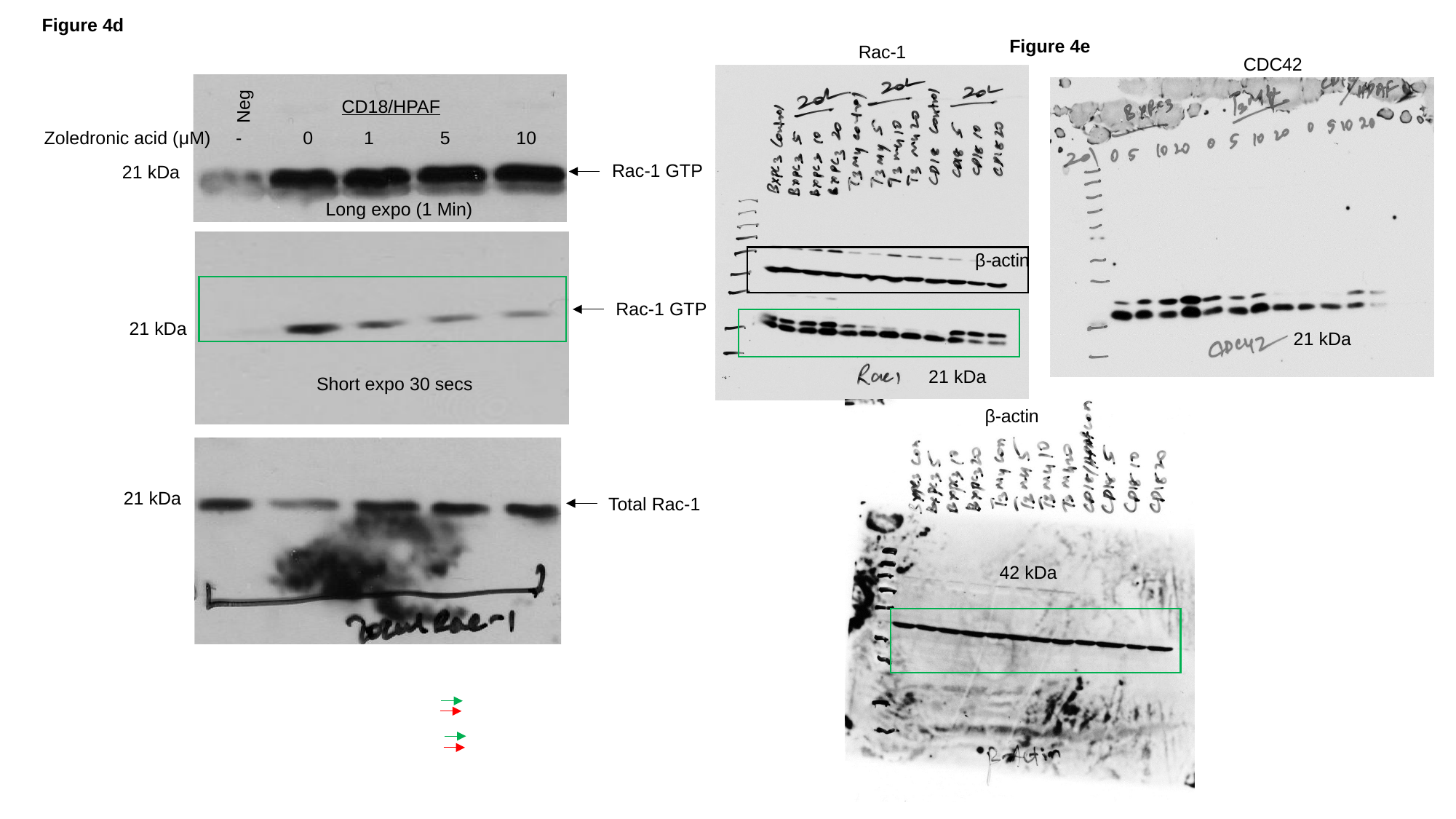

Figure 4d
Figure 4e
Rac-1
CDC42
Neg
Zoledronic acid (μM) -	 0 1 5 10
CD18/HPAF
Rac-1 GTP
21 kDa
Long expo (1 Min)
β-actin
Rac-1 GTP
21 kDa
21 kDa
21 kDa
Short expo 30 secs
β-actin
21 kDa
Total Rac-1
42 kDa

## Slide 4
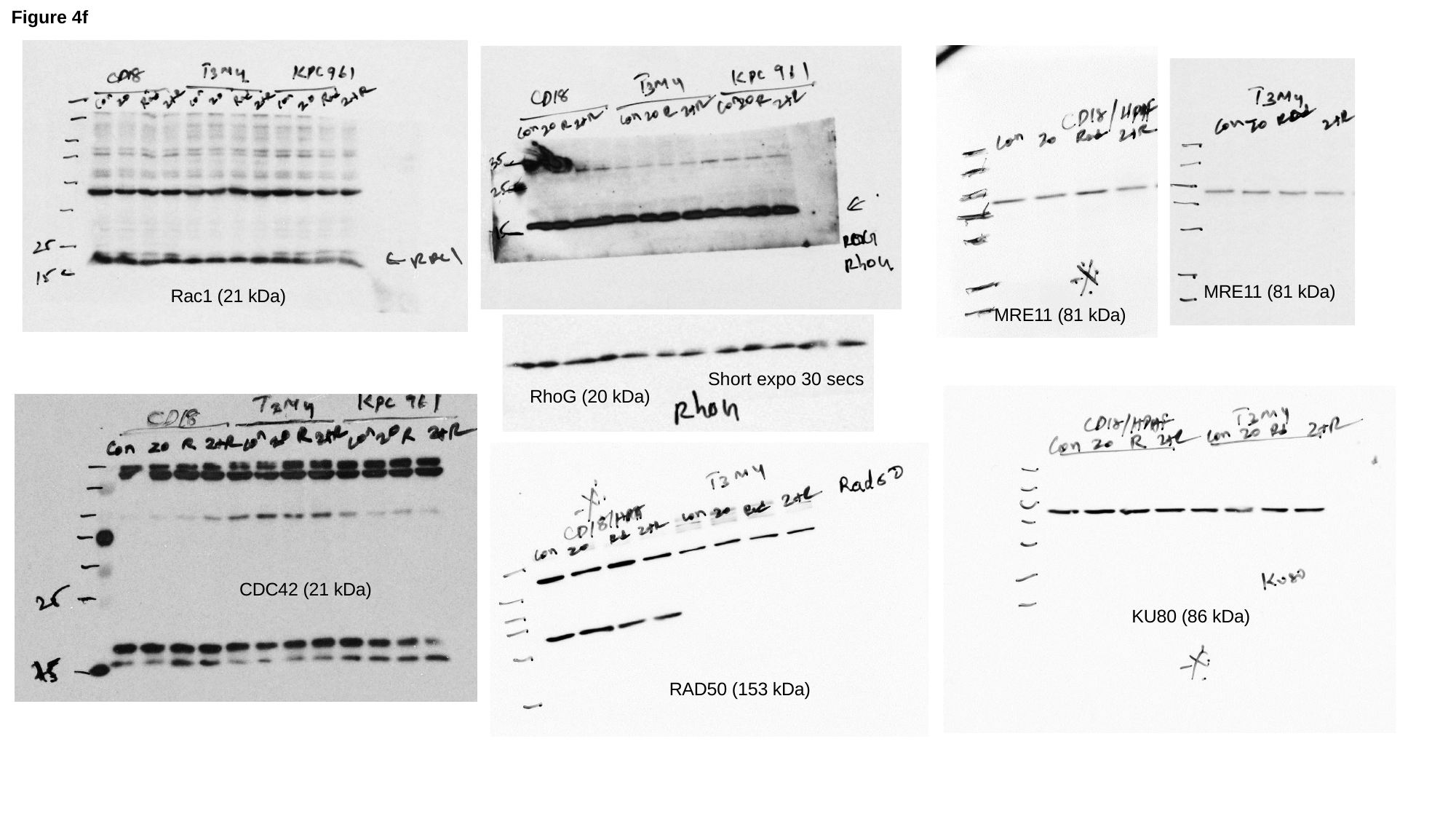

Figure 4f
Rac1 (21 kDa)
MRE11 (81 kDa)
MRE11 (81 kDa)
Short expo 30 secs
RhoG (20 kDa)
KU80 (86 kDa)
CDC42 (21 kDa)
RAD50 (153 kDa)

## Slide 5
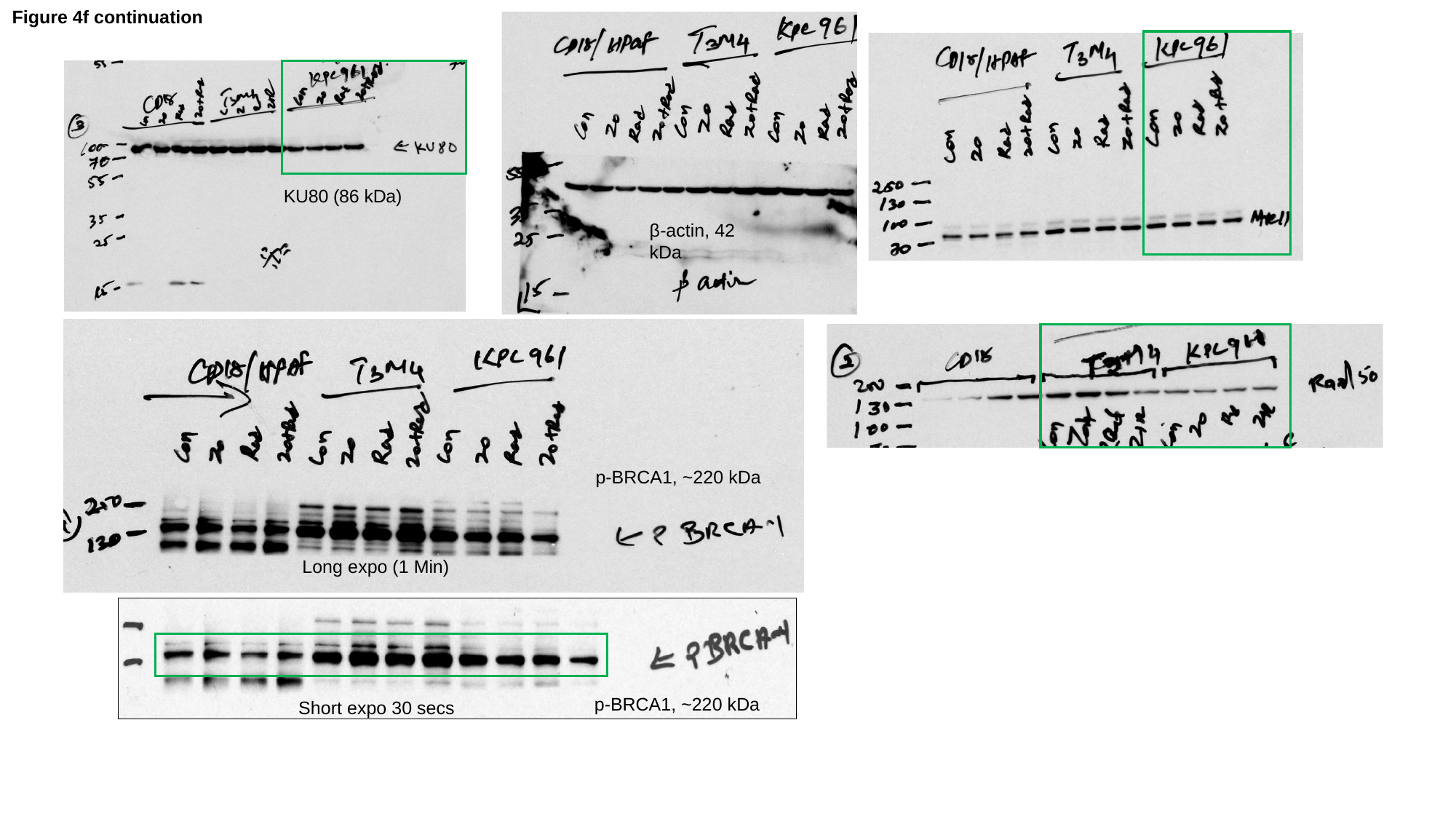

Figure 4f continuation
KU80 (86 kDa)
β-actin, 42 kDa
p-BRCA1, ~220 kDa
Long expo (1 Min)
p-BRCA1, ~220 kDa
Short expo 30 secs

## Slide 6
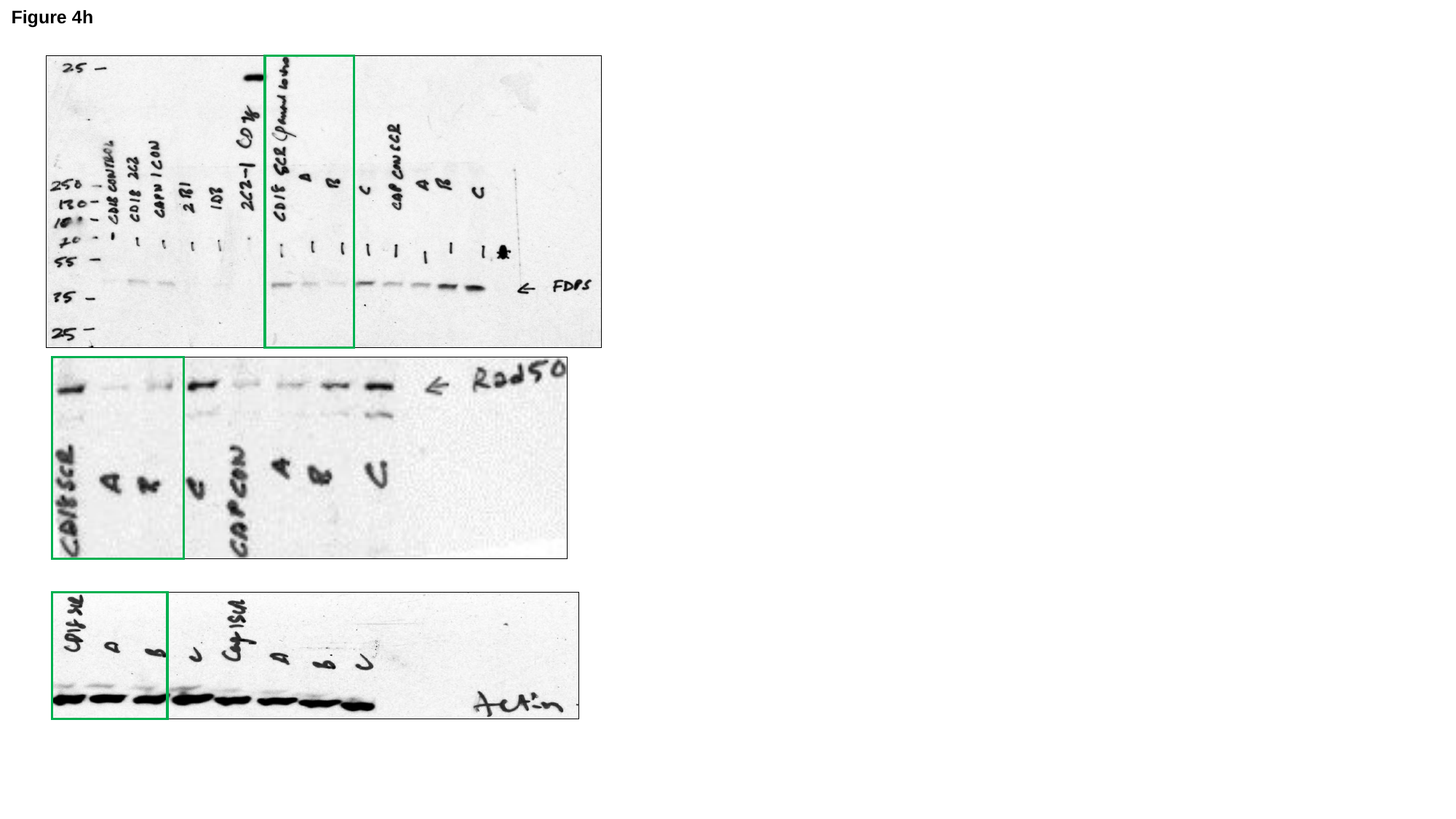

Figure 4h

## Slide 7
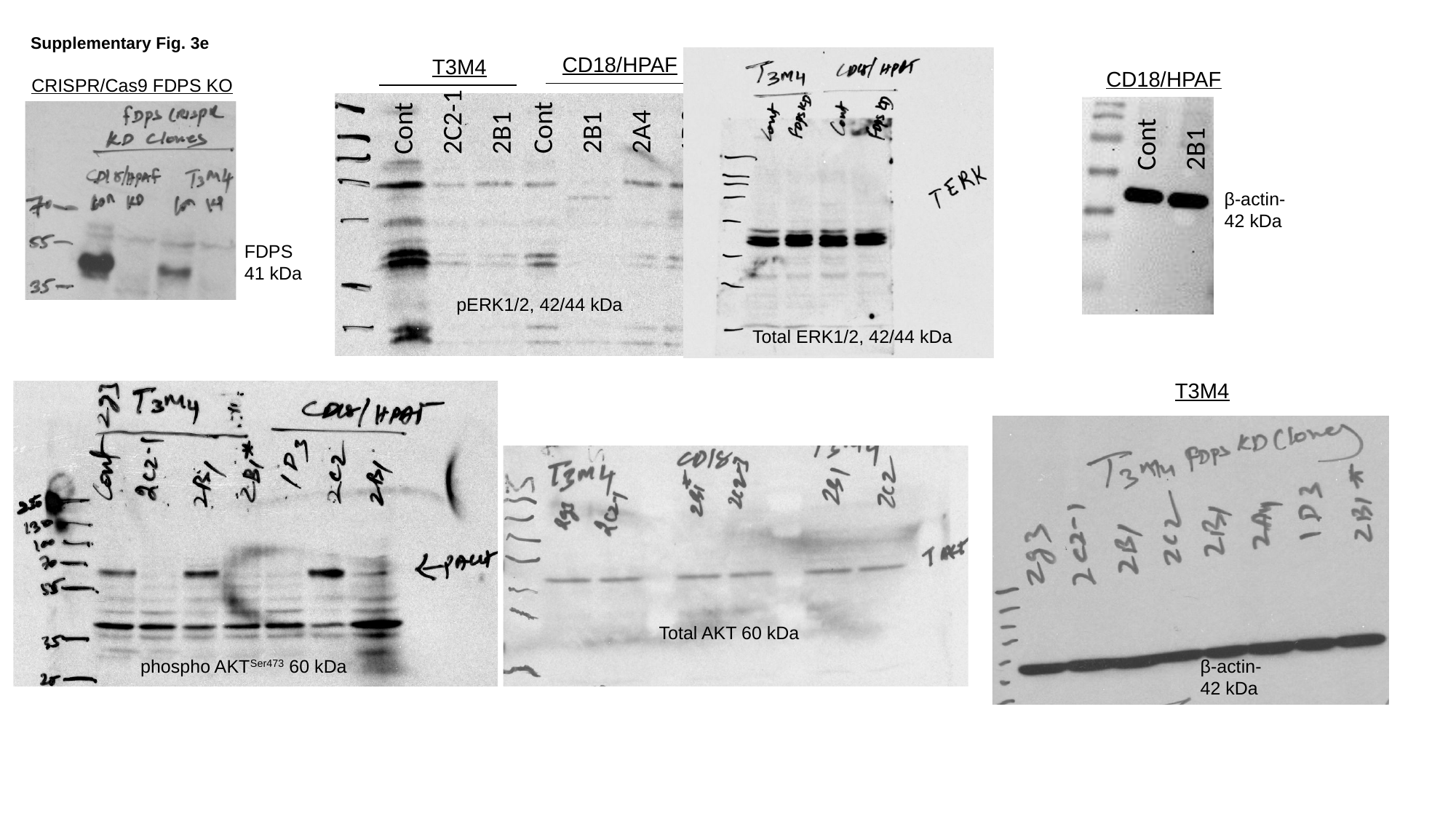

Supplementary Fig. 3e
Cont
2B1
2A4
1D3
Cont
2C2-1
2B1
CD18/HPAF
T3M4
pERK1/2, 42/44 kDa
CD18/HPAF
CRISPR/Cas9 FDPS KO
Cont
2B1
β-actin- 42 kDa
FDPS
41 kDa
Total ERK1/2, 42/44 kDa
T3M4
Total AKT 60 kDa
phospho AKTSer473 60 kDa
β-actin- 42 kDa

## Slide 8
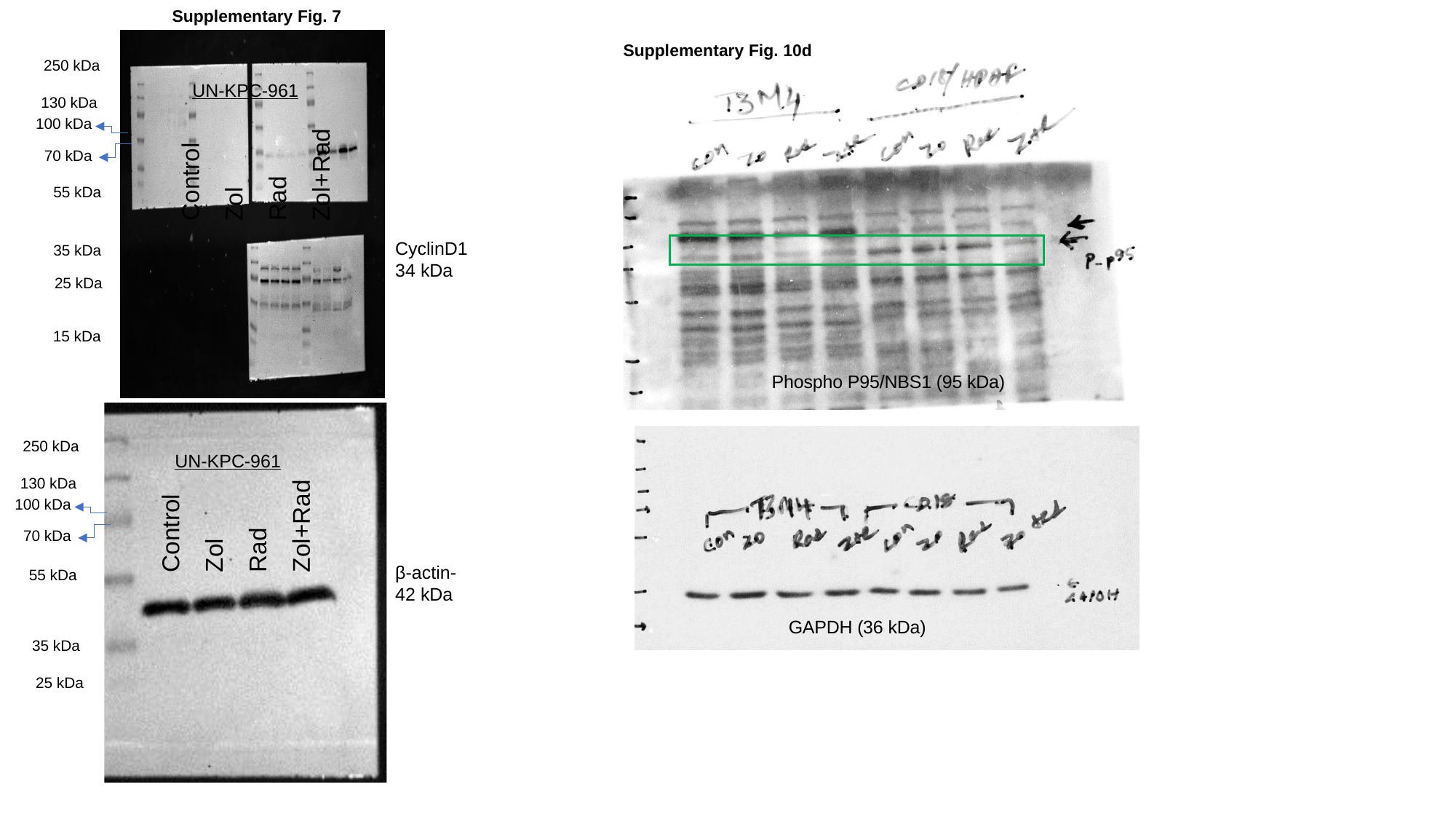

Supplementary Fig. 7
Supplementary Fig. 10d
 250 kDa
 130 kDa
 100 kDa
 70 kDa
 55 kDa
 35 kDa
 25 kDa
 15 kDa
Phospho P95/NBS1 (95 kDa)
UN-KPC-961
Control
Zol
Rad
Zol+Rad
CyclinD1
34 kDa
Control
Zol
Rad
Zol+Rad
 250 kDa
 130 kDa
 100 kDa
 70 kDa
 55 kDa
 35 kDa
 25 kDa
UN-KPC-961
β-actin- 42 kDa
GAPDH (36 kDa)
