# Supplementary material for: Disruption of FDPS/Rac1 axis radiosensitizes pancreatic ductal adenocarcinoma by attenuating DNA damage response and immunosuppressive signalling
Source: eBioMedicine. 2021 Dec 28;75:103772. doi: 10.1016/j.ebiom.2021.103772 (PMC8718746; doi:10.1016/j.ebiom.2021.103772)
Supplement: Supplementary file 2 [file mmc2.pdf]

SUPPLEMENTARY MATERIALS FOR

Disruption of FDPS/Rac1 axis radiosensitizes pancreatic ductal adenocarcinoma by attenuating DNA damage response and immunosuppressive signaling

Parthasarathy Seshacharyulu, Sushanta Halder, Ramakrishna Nimmakayala, Satyanarayana Rachagani, Sanjib Chaudhary, Pranita Atri, Ramakanth Chirravuri-Venkata, Michel M. Ouellette, Joseph Carmicheal, Shailendra K. Gautam, Raghupathy Vengoji, Shuo Wang, Sicong Li, Lynette Smith, Geoffrey A. Talmon, Kelsey Klute, Quan Ly, Bradley N Reames, Jean L Grem, Lyudmyla Berim, James C Padussis, Sukhwinder Kaur, Sushil Kumar, Moorthy P. Ponnusamy, Maneesh Jain, Chi Lin, and Surinder K Batra

Correspondence to: clin@unmc.edu and sbatra@unmc.edu

This file includes:

Tables S1 to S12
Figures S1 to S14
Clinical Trial Protocol

Table S1. Patient characteristics

		N=14
FDPS Composite score	Median (Range)	4 (0-12)
KI67 per cent positive	Median (Range)	4.5 (0-18)
Age	Median (Range)	64 (47-79)
Sex	F<br>M	6 (43%)<br>8 (57%)
Treatment	5FU<br>Nelfinavir<br>Xeloda<br>Missing	1 (8%)<br>10 (83%)<br>1 (8%)<br>2
RT Dose	2500<br>3000<br>3500<br>4000	3 (21%)<br>1 (7%)<br>5 (36%)<br>5 (36%)
Location	Head<br>Uncinate	13 (93%)<br>1 (7%)
T	2<br>3<br>4	2 (14%)<br>7 (50%)<br>5 (36%)
N1	0<br>1	5 (36%)<br>9 (64%)
M	0	14 (100%)
Radiation response	Complete<br>Near Complete<br>Partial<br>Poor or no response	1 (7%)<br>6 (43%)<br>5 (36%)<br>2 (14%)

Table S2. Pathological evaluation criteria and classification of radiation response

**Pathological Score**	**Pathological Description**	**Radiation Response**
0	No viable cancer cells	Complete
1	Single or Rare and small group of cancer cells	Near Complete
2	Existence of Residual Cancer Cells	Partial Response
3	Presence of Extensive Tumour Cells	Poor or No Response

Table S3. Comparison of FDPS and KI67 by tumour response

Label	Tumour regression score	N	Mean	SD	Median	Minimum	Maximum	p-value
FDPS intensity	Score 0/1	7	1.0	0.8	1	0	2	0.22
	Score 2/3	7	1.7	1.0	1	1	3	
FDPS per cent positive	Score 0/1	7	2.4	1.7	3	0	4	0.064
	Score 2/3	7	3.6	1.1	4	1	4	
FDPS Composite score	Score 0/1	7	3.4	2.9	3	0	8	0.15
	Score 2/3	7	6.4	4.3	4	1	12	
KI67 per cent positive	Score 0/1	7	3.2	5.4	0.5	0	15	0.040
	Score 2/3	7	7.6	5.3	7	1	18	

*Wilcoxon rank-sum test

Table S4. Mean estimates on the natural log scale.

Day	Treatment group	Mean Estimate	Standard Error	Lower	Upper
**Day 0**	**Control**	15.0033	0.7316	13.5247	16.4819
**Day 0**	**Radiation**	13.0607	0.7316	11.5821	14.5393
**Day 0**	**Zol**	13.0877	0.7316	11.6091	14.5664
**Day 0**	**Zol+Rad**	13.9937	0.7316	12.5151	15.4724
**Day 7**	**Control**	16.5970	0.7316	15.1183	18.0756
**Day 7**	**Radiation**	15.1182	0.7316	13.6396	16.5968
**Day 7**	**Zol**	15.3687	0.7316	13.8901	16.8473
**Day 7**	**Zol+Rad**	13.0030	0.7316	11.5243	14.4816
**Day 21**	**Control**	16.4812	0.7316	15.0026	17.9598
**Day 21**	**Radiation**	16.5777	0.7316	15.0991	18.0563
**Day 21**	**Zol**	16.3474	0.7316	14.8687	17.8260
**Day 21**	**Zol+Rad**	13.2324	0.7316	11.7538	14.7110

Table S4 contains the estimated means and 95% CI at each day and group combination on the natural log scale.

Table S5. Pairwise comparisons between Days at each treatment group on the natural log scale.

Adjustment for Multiple Comparisons: Tukey-Kramer													
**Simple Effect Level**	**Day**	**Day**	**Estimate**	**Standard Error**	**DF**	**t Value**	**Pr > |t|**	**Adj P**	**Alpha**	**Lower**	**Upper**	**Adj Lower**	**Adj Upper**
**Control**	**Day 0**	**Day 21**	-1.4779	0.7915	40	-1.87	0.0692	0.1615	0.05	-3.0776	0.1218	-3.4044	0.4486
**Control**	**Day 0**	**Day 7**	-1.5937	0.7915	40	-2.01	0.0508	0.1221	0.05	-3.1934	0.006076	-3.5201	0.3328
**Control**	**Day 21**	**Day 7**	-0.1157	0.7915	40	-0.15	0.8845	0.9883	0.05	-1.7155	1.4840	-2.0422	1.8108
**Rad**	**Day 0**	**Day 21**	-3.5170	0.7915	40	-4.44	<.0001	0.0002	0.05	-5.1167	-1.9173	-5.4435	-1.5905
**Rad**	**Day 0**	**Day 7**	-2.0575	0.7915	40	-2.60	0.0130	0.0341	0.05	-3.6572	-0.4578	-3.9840	-0.1310
**Rad**	**Day 21**	**Day 7**	1.4595	0.7915	40	1.84	0.0726	0.1685	0.05	-0.1402	3.0593	-0.4670	3.3860
**Zol**	**Day 0**	**Day 21**	-3.2596	0.7915	40	-4.12	0.0002	0.0005	0.05	-4.8594	-1.6599	-5.1861	-1.3331
**Zol**	**Day 0**	**Day 7**	-2.2810	0.7915	40	-2.88	0.0063	0.0170	0.05	-3.8807	-0.6812	-4.2075	-0.3545
**Zol**	**Day 21**	**Day 7**	0.9787	0.7915	40	1.24	0.2235	0.4391	0.05	-0.6211	2.5784	-0.9478	2.9051
**Zol+Rad**	**Day 0**	**Day 21**	0.7613	0.7915	40	0.96	0.3419	0.6049	0.05	-0.8384	2.3611	-1.1652	2.6878
**Zol+Rad**	**Day 0**	**Day 7**	0.9908	0.7915	40	1.25	0.2179	0.4304	0.05	-0.6089	2.5905	-0.9357	2.9173
**Zol+Rad**	**Day 21**	**Day 7**	0.2294	0.7915	40	0.29	0.7734	0.9548	0.05	-1.3703	1.8292	-1.6970	2.1559

Table S5 shows pairwise differences between the days for each of the treatment groups. There were no significant differences over time in the control or Zol+Rad group. However, the Radiation and Zol groups exhibited significant differences between Day 0 and Day 7 and Day 0 and Day 21.

Table S6. Pairwise comparisons between Treatments for each day on the natural log scale.

Simple Effect Comparisons of Day*Treatment group Least Squares Means By Day<br>Adjustment for Multiple Comparisons: Tukey													
**Simple Effect Level**	**Treatment group**	**Treatment group**	**Estimate**	**Standard Error**	**DF**	**t Value**	**Pr > |t|**	**Adj P**	**Alpha**	**Lower**	**Upper**	**Adj Lower**	**Adj Upper**
**Day 0**	**Control**	**Radiation**	1.9426	1.0346	40	1.88	0.0677	0.2538	0.05	-0.1485	4.0337	-0.8306	4.7159
**Day 0**	**Control**	**Zol**	1.9156	1.0346	40	1.85	0.0715	0.2651	0.05	-0.1755	4.0067	-0.8577	4.6888
**Day 0**	**Control**	**Zol+Rad**	1.0096	1.0346	40	0.98	0.3350	0.7639	0.05	-1.0815	3.1006	-1.7637	3.7828
**Day 0**	**Radiation**	**Zol**	-0.02705	1.0346	40	-0.03	0.9793	1.0000	0.05	-2.1181	2.0640	-2.8003	2.7462
**Day 0**	**Radiation**	**Zol+Rad**	-0.9331	1.0346	40	-0.90	0.3726	0.8039	0.05	-3.0241	1.1580	-3.7063	1.8402
**Day 0**	**Zol**	**Zol+Rad**	-0.9060	1.0346	40	-0.88	0.3864	0.8174	0.05	-2.9971	1.1851	-3.6793	1.8673
**Day 7**	**Control**	**Radiation**	1.4788	1.0346	40	1.43	0.1607	0.4890	0.05	-0.6123	3.5699	-1.2945	4.2521
**Day 7**	**Control**	**Zol**	1.2283	1.0346	40	1.19	0.2422	0.6384	0.05	-0.8628	3.3193	-1.5450	4.0015
**Day 7**	**Control**	**Zol+Rad**	3.5940	1.0346	40	3.47	0.0012	0.0066	0.05	1.5029	5.6851	0.8207	6.3673
**Day 7**	**Radiation**	**Zol**	-0.2505	1.0346	40	-0.24	0.8099	0.9949	0.05	-2.3416	1.8405	-3.0238	2.5227
**Day 7**	**Radiation**	**Zol+Rad**	2.1152	1.0346	40	2.04	0.0475	0.1892	0.05	0.02413	4.2063	-0.6581	4.8885
**Day 7**	**Zol**	**Zol+Rad**	2.3657	1.0346	40	2.29	0.0276	0.1183	0.05	0.2747	4.4568	-0.4075	5.1390
**Day 21**	**Control**	**Radiation**	-0.09647	1.0346	40	-0.09	0.9262	0.9997	0.05	-2.1875	1.9946	-2.8697	2.6768
**Day 21**	**Control**	**Zol**	0.1339	1.0346	40	0.13	0.8977	0.9992	0.05	-1.9572	2.2249	-2.6394	2.9071
**Day 21**	**Control**	**Zol+Rad**	3.2488	1.0346	40	3.14	0.0032	0.0161	0.05	1.1577	5.3399	0.4756	6.0221
**Day 21**	**Radiation**	**Zol**	0.2303	1.0346	40	0.22	0.8250	0.9960	0.05	-1.8607	2.3214	-2.5429	3.0036

Simple Effect Comparisons of Day*Treatment group Least Squares Means By Day<br>Adjustment for Multiple Comparisons: Tukey													
**Simple Effect Level**	**Treatment group**	**Treatment group**	**Estimate**	**Standard Error**	**DF**	**t Value**	**Pr > |t|**	**Adj P**	**Alpha**	**Lower**	**Upper**	**Adj Lower**	**Adj Upper**
**Day 21**	**Radiation**	**Zol+Rad**	3.3453	1.0346	40	3.23	0.0025	0.0126	0.05	1.2542	5.4364	0.5720	6.1185
**Day 21**	**Zol**	**Zol+Rad**	3.1149	1.0346	40	3.01	0.0045	0.0224	0.05	1.0239	5.2060	0.3417	5.8882

Table S6 shows differences between groups on each day. There was no significant difference between treatment groups at Day 0. On day 7, Control and Zol+Rad are different (p=0.0066). At day 21 Control and Zol+Rad are different (p=0.016), Rad and Zol+Rad are different (p=0.013), and Zol and Zol+Rad are different (p=0.022).

Table S7. Mean estimates on the natural log scale.

group	Mean Estimate	Standard Error	Lower	Upper
**Control**	-0.2392	0.1230	-0.4957	0.01742
**Rad**	-1.4851	0.1230	-1.7417	-1.2285
**Zol**	-1.1384	0.1230	-1.3950	-0.8819
**Zol+Rad**	-1.8550	0.1230	-2.1115	-1.5984

The overall test for group differences was significant (p<0.0001). Table S7 shows mean estimates for the treatment groups on the natural log scale with 95% CI.

Table S8. Pairwise comparisons between treatment groups.

<table>
<tr><th colspan="13">Differences of group Least Squares Means
Adjustment for Multiple Comparisons: Tukey</th></tr>
<tr><th>Group</th><th>Group</th><th>Estimate</th><th>SE</th><th>DF</th><th>t Value</th><th>Pr > |t|</th><th>Adj P</th><th>Alpha</th><th>Lower</th><th>Upper</th><th>Adj Lower</th><th>Adj Upper</th></tr>
<tr><td>**Control**</td><td>**Rad**</td><td>1.2459</td><td>0.1739</td><td>20</td><td>7.16</td><td><.0001</td><td><.0001</td><td>0.05</td><td>0.8831</td><td>1.6088</td><td>0.7591</td><td>1.7328</td></tr>
<tr><td>**Control**</td><td>**Zol**</td><td>0.8993</td><td>0.1739</td><td>20</td><td>5.17</td><td><.0001</td><td>0.0003</td><td>0.05</td><td>0.5364</td><td>1.2621</td><td>0.4124</td><td>1.3861</td></tr>
<tr><td>**Control**</td><td>**Zol+Rad**</td><td>1.6158</td><td>0.1739</td><td>20</td><td>9.29</td><td><.0001</td><td><.0001</td><td>0.05</td><td>1.2530</td><td>1.9787</td><td>1.1290</td><td>2.1027</td></tr>
<tr><td>**Rad**</td><td>**Zol**</td><td>-0.3467</td><td>0.1739</td><td>20</td><td>-1.99</td><td>0.0601</td><td>0.2237</td><td>0.05</td><td>-0.7095</td><td>0.01619</td><td>-0.8335</td><td>0.1402</td></tr>
<tr><td>**Rad**</td><td>**Zol+Rad**</td><td>0.3699</td><td>0.1739</td><td>20</td><td>2.13</td><td>0.0461</td><td>0.1789</td><td>0.05</td><td>0.007038</td><td>0.7327</td><td>-0.1170</td><td>0.8567</td></tr>
<tr><td>**Zol**</td><td>**Zol+Rad**</td><td>0.7165</td><td>0.1739</td><td>20</td><td>4.12</td><td>0.0005</td><td>0.0028</td><td>0.05</td><td>0.3537</td><td>1.0794</td><td>0.2297</td><td>1.2034</td></tr>
</table>

Table S8 shows pairwise differences between the groups. The control group is significantly higher than all the other groups, and there is a significant difference between Zol and Zol+Rad treated xenograft tumours weights (p=0.0028).

Table S9. Mean estimates on the square root scale.

Day	Treatment group	Mean Estimate	Standard Error	Lower	Upper
**Day 0**	**Control**	1836.28	545.23	648.33	3024.23
**Day 0**	**Rad**	1459.17	545.23	271.22	2647.12
**Day 0**	**Zol**	2206.80	545.23	1018.86	3394.75
**Day 0**	**Zol+Rad**	2354.41	545.23	1166.46	3542.36
**Day 7**	**Control**	5968.92	545.23	4780.98	7156.87
**Day 7**	**Rad**	2672.72	545.23	1484.77	3860.67
**Day 7**	**Zol**	5462.28	545.23	4274.33	6650.23
**Day 7**	**Zol+Rad**	1908.47	545.23	720.52	3096.42

There was a significant interaction between the treatment group and day, indicating the treatment effect differed by day (p=0.0008).

Table S9 contains the estimated means and 95% CI at each day and group combination on the square root scale.

Table S10. Pairwise comparisons between Days at each treatment group on the square root scale.

Adjustment for Multiple Comparisons: Tukey-Kramer													
**Simple Effect Level**	**Day**	**Day**	**Estimate**	**Standard Error**	**DF**	**t Value**	**Pr > |t|**	**Adj P**	**Alpha**	**Lower**	**Upper**	**Adj Lower**	**Adj Upper**
**Control**	**Day 0**	**Day 7**	-4132.64	612.26	12	-6.75	<.0001	<.0001	0.05	-5466.64	-2798.64	-5466.59	-2798.70
**Rad**	**Day 0**	**Day 7**	-1213.56	612.26	12	-1.98	0.0708	0.0708	0.05	-2547.55	120.44	-2547.50	120.39
**Zol**	**Day 0**	**Day 7**	-3255.47	612.26	12	-5.32	0.0002	0.0002	0.05	-4589.47	-1921.47	-4589.42	-1921.53
**Zol+Rad**	**Day 0**	**Day 7**	445.95	612.26	12	0.73	0.4804	0.4804	0.05	-888.05	1779.94	-888.00	1779.89

Table S10 shows pairwise differences between the days for each of the treatment groups. There were no significant differences over time in the Rad or Zol+Rad group. However, significant differences between Day 0 and Day 7 were observed in the control, and Zol treated groups.

Table S11. Pairwise comparisons between Treatments for each day on the square root scale.

Adjustment for Multiple Comparisons: Tukey													
**Simple Effect Level**	**Treatment group**	**Treatment group**	**Estimate**	**Standard Error**	**DF**	**t Value**	**Pr > |t|**	**Adj P**	**Alpha**	**Lower**	**Upper**	**Adj Lower**	**Adj Upper**
**Day 0**	**Control**	**Rad**	377.12	771.07	12	0.49	0.6336	0.9601	0.05	-1302.90	2057.13	-1912.03	2666.26
**Day 0**	**Control**	**Zol**	-370.52	771.07	12	-0.48	0.6395	0.9620	0.05	-2050.53	1309.49	-2659.66	1918.62
**Day 0**	**Control**	**Zol+Rad**	-518.13	771.07	12	-0.67	0.5143	0.9057	0.05	-2198.14	1161.89	-2807.27	1771.02
**Day 0**	**Rad**	**Zol**	-747.64	771.07	12	-0.97	0.3514	0.7688	0.05	-2427.65	932.38	-3036.78	1541.51
**Day 0**	**Rad**	**Zol+Rad**	-895.25	771.07	12	-1.16	0.2682	0.6610	0.05	-2575.26	784.77	-3184.39	1393.90
**Day 0**	**Zol**	**Zol+Rad**	-147.61	771.07	12	-0.19	0.8514	0.9974	0.05	-1827.62	1532.41	-2436.75	2141.54
**Day 7**	**Control**	**Rad**	3296.20	771.07	12	4.27	0.0011	0.0051	0.05	1616.19	4976.22	1007.06	5585.35
**Day 7**	**Control**	**Zol**	506.65	771.07	12	0.66	0.5235	0.9110	0.05	-1173.37	2186.66	-1782.50	2795.79
**Day 7**	**Control**	**Zol+Rad**	4060.46	771.07	12	5.27	0.0002	0.0010	0.05	2380.44	5740.47	1771.31	6349.60
**Day 7**	**Rad**	**Zol**	-2789.55	771.07	12	-3.62	0.0035	0.0161	0.05	-4469.57	-1109.54	-5078.70	-500.41
**Day 7**	**Rad**	**Zol+Rad**	764.26	771.07	12	0.99	0.3412	0.7571	0.05	-915.76	2444.27	-1524.89	3053.40
**Day 7**	**Zol**	**Zol+Rad**	3553.81	771.07	12	4.61	0.0006	0.0029	0.05	1873.80	5233.82	1264.67	5842.95

Table S11 shows differences between groups on each day. There was no significant difference between treatment groups at Day 0. However, at day 7, statistical difference was observed among groups (Control vs. Rad (P=0.0051), Control vs. Zol+Rad (P=0.0010), Rad vs. Zol (P=0.0161), and Zol vs. Zol+Rad (P=0.0029)).

Table S12. Primers used in the RT-qPCR analysis.

Gene	Forward primer	Reverse primer
TGFβ3	TCACCACAACCCTCATCTAATC	CCTGTCGGAAGTCAATGTAGAG
FGF2	CAAGCAGAAGAGAGAGGAGTTG	TCGTTTCAGTGCCACATACC
MIF	CGGACAGGGTCTACATCAACTA	TCTTAGGCGAAGGTGGAGTT
PDGFRβ	GCCTCACCACATCCGCTC	TCACACTCTTCCGTCACATTGC
IL6	TTCGGTCCAGTTGACTTCTCC	AAGAGGTGAGTGTCTGTCTGT

Fig. S1

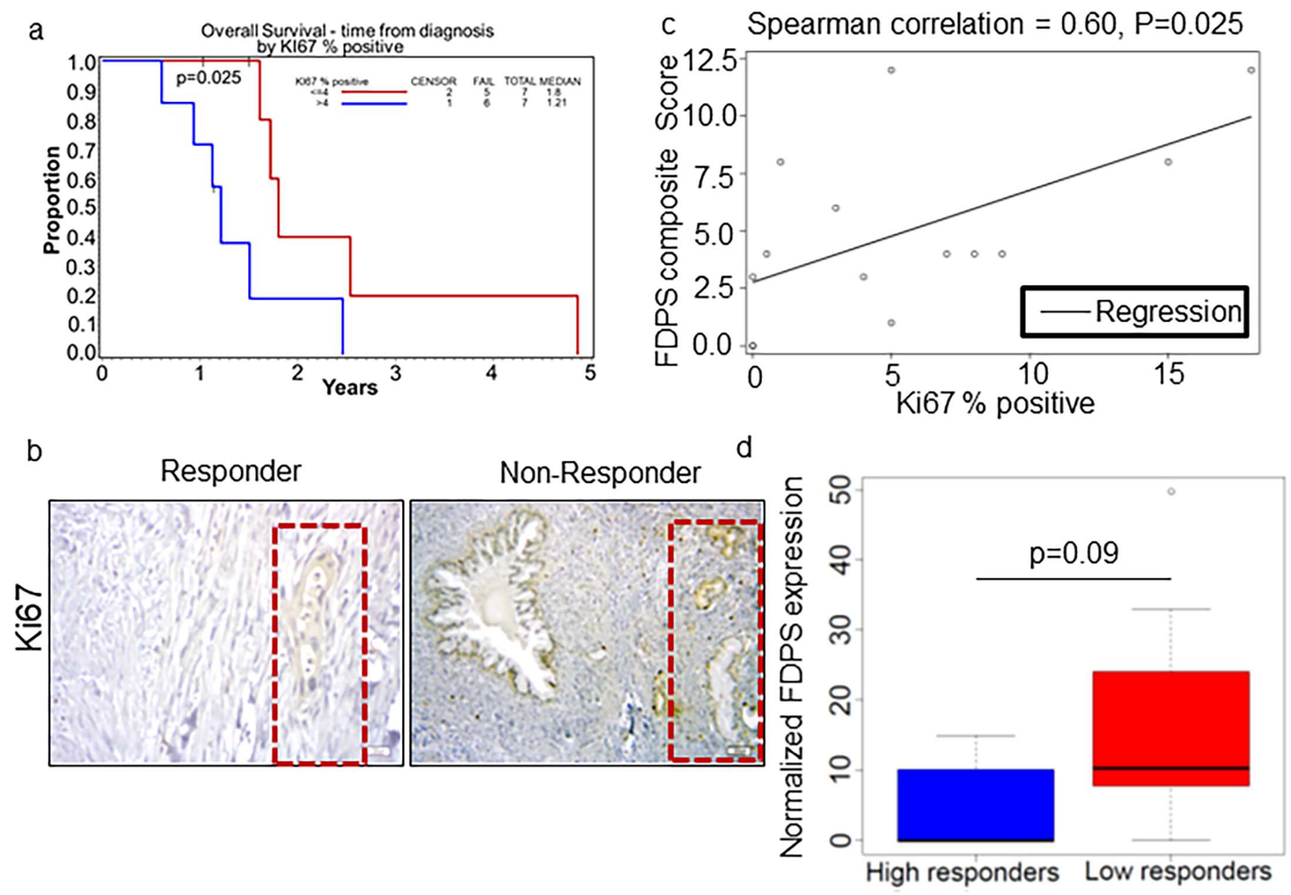

Fig. S1. FDPS and Ki67 expression predict which PDAC patients were radioresponders and non-responders. Serial sections used to detect FDPS protein expression were immunostained for Ki67 protein. (a) Kaplan-Meier survival curves were generated based on the Ki67 composite score, and the overall survival difference between radioresponder and non-responder PDAC patients was analysed. (b) Representative microscopic images show Ki67 nuclear expression in PDAC patient tissue categorized by the response. (c) Spearman correlation coefficient between FDPS and Ki67 (P=0.025) confirms positive co-expression in radioresponder and non-responder tissues. (d) Box and whisker plots show normalized *FDPS* expression and its relationship between high and low responses to RT. (p=0.09, Wilcoxon Test, High Responders (n=7), Low Responders (n=8))

Fig. S2

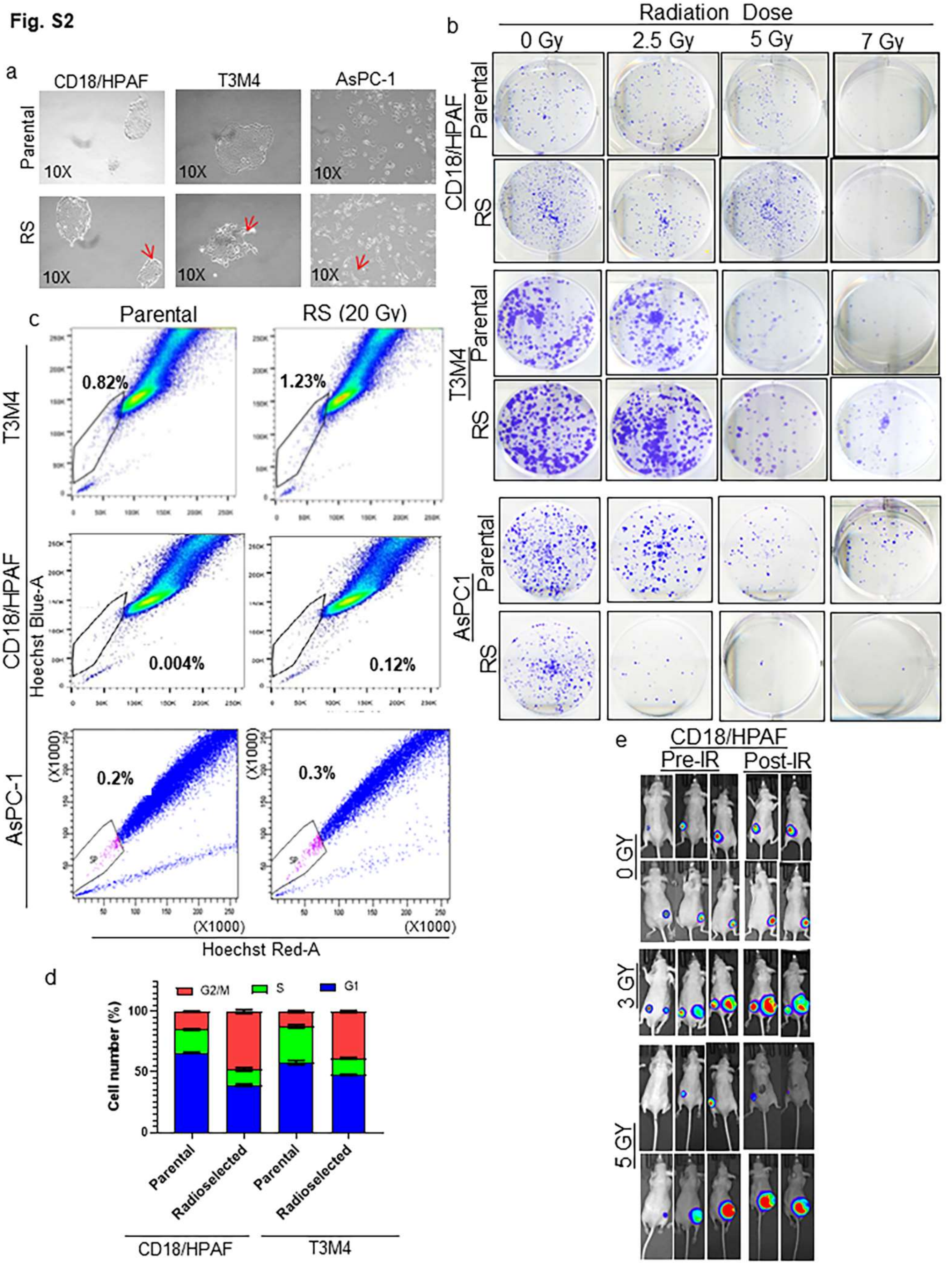
Fig. S2
a
CD18/HPAF
T3M4
AsPC-1
Parental
RS
10X
b
Radiation Dose
0 Gy
2.5 Gy
5 Gy
7 Gy
CD18/HPAF
T3M4
AsPC1
Parental
RS
c
Parental
RS (20 Gy)
T3M4
CD18/HPAF
AsPC-1
0.82%
1.23%
0.004%
0.12%
0.2%
0.3%
Hoechst Blue-A
Hoechst Red-A
(X1000)
d
G2/M
S
G1
Cell number (%)
Parental
Radioselected
CD18/HPAF
T3M4
e
CD18/HPAF
Pre-IR
Post-IR
0 GY
3 GY
5 GY

Fig. S2. FDPS expression is associated with radiosensitivity in PDAC cells. PDAC cell lines expressing high and low FDPS were treated with a fractionated irradiation dose of approximately 2 Gy once a day for five days a week, over a period of two weeks, using a linear accelerator. (a) Morphological analysis of cells in response to RT is a qualitative measure to establish and discriminate an RR isogenic cell line from its parental cell line. The parental CD18/HPAF, T3M4, and AsPC1 cells, along with their matched isogenic cell lines, were imaged (10X) using a light microscope. The upper panel represents images of parental PDAC cells, and the lower panel depicts the images of isogeneic RS PDAC cells. (b) Representative crystal violet-stained colonies survived RT (0, 2.5, 5, and 7 Gy), demonstrating relative RR between parental and RS isogenic PDAC cells. (c) Side populations (SP) are enriched in RS isogenic PDAC cells due to persistent exposure to radiation. RS and parental cells were analysed using the Hoechst 33342 dye efflux protocol and FACS analysis. SP phenotype is retained and enriched in isogenic RS cell lines as compared to parental cells. Verapamil was used to confirm SP. (d) The stacked bar graph demonstrates the average percentage of parental and RR PDAC cells in various cell cycle phases (n=3). (e) *In vivo* validation of RR in PDAC cells. Bioluminescence images of subcutaneously implanted parental and isogenic RS PDAC cells on the contralateral flanks of athymic nude mice. In vivo monitoring of the response in xenograft parental and RS PDAC cells three weeks before IR treatment and two weeks after IR treatment.

Fig. S3

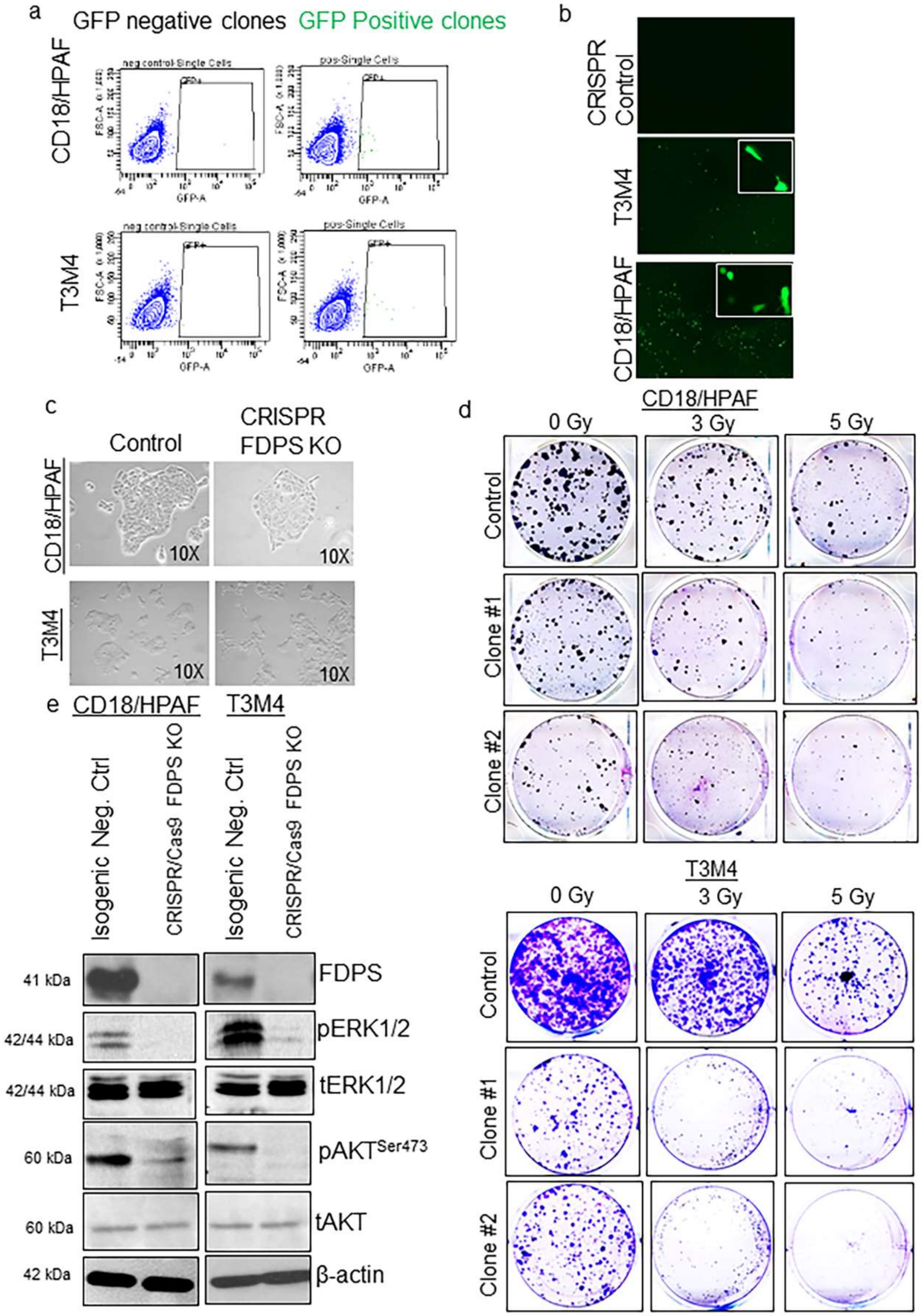

Fig. S3. Radiosensitization mediated by FDPS inhibition in PDAC cells. CRISPR knockout (KO) of FDPS in PDAC cells validates the direct cause-effect relationship of FDPS with radiosensitization. (a) FACS analysis shows a gating strategy for isolating Cas9-GFP-positive single PDAC cells. Passage-matched untransfected cells

served as a negative control. (b) Florescence microscope images show transfection efficiency of pSpCas9 BB-2A-GFP-FDPS gRNAs into PDAC cells. (c) Light microscopic images demonstrate the morphology of parental PDAC control cells and FDPS-CRISPR KO clones. (d) FDPS KO clones and parental control PDAC cells (CD18/HPAF and T3M4) were exposed to a single fraction of RT (3 or 5 Gy), incubated for 10 days, and stained with crystal violet. Staining exhibited a decrease in the number of colonies in FDPS CRISPR KO clones relative to controls. (e) Western blots probed with FDPS, phospho-AKT, and phospho-ERK antibodies confirm decreased FDPS protein with corresponding decreased activated ERK and AKT, but no change in respective total protein in the KO clones compared to control cells.

Fig. S4

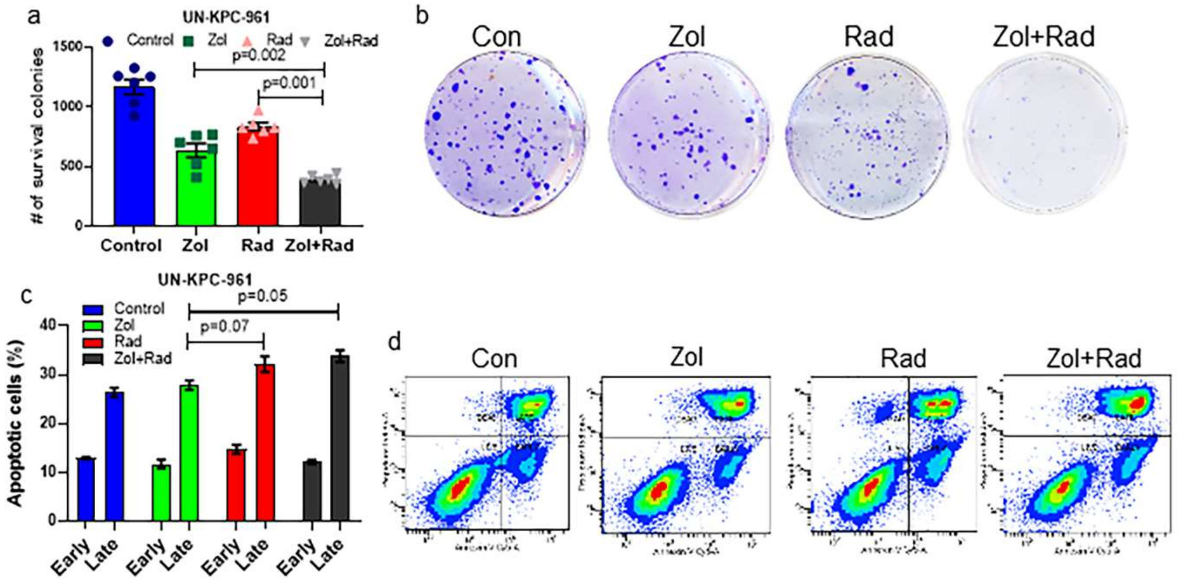

Fig. S4. Zol radiosensitizes murine UN-KPC-961 cells by decreasing the number of survival colonies and increasing apoptosis. (a) Murine syngeneic PDAC cells were treated with Zol (5µM) 4 hours before radiation and treated with 1 fraction of 7 Gy RT and incubated for 12 days. Viable colonies were stained with 0.5% crystal violet in 25% methanol. Colonies were counted using Image J software, and a scatter plot with a bar graph represents the number of survival colonies after Zol or RT alone and in combination (Zol *vs.* Zol+Rad (p=0.002), Rad *vs.* Zol+Rad (p=0.001), n=6). (b) Representative colonies survived after Zol and RT treatment. (c) UN-KPC-961 cells were treated with Zol and RT, co-stained with Annexin V-FITC and propidium iodide (PI), and analysed by flow cytometry. The bar graph show percentage of cells that underwent early and late apoptosis in the presence and absence of Zol and RT in UN-KPC-961 cells (Zol *vs.* Rad (p=0.07, late apoptosis), Zol *vs.* Zol+Rad (p=0.05, late apoptosis), n=3). (d) Representative scatter plots indicate the percentage of normal (both Annexin V and PI negative), early apoptotic (Annexin V positive and PI negative), late apoptotic (both Annexin V and PI positive), and dead (Annexin V negative and PI-positive) cells in the respective quadrant. Statistical analysis was done by student's t-test.

Fig. S5

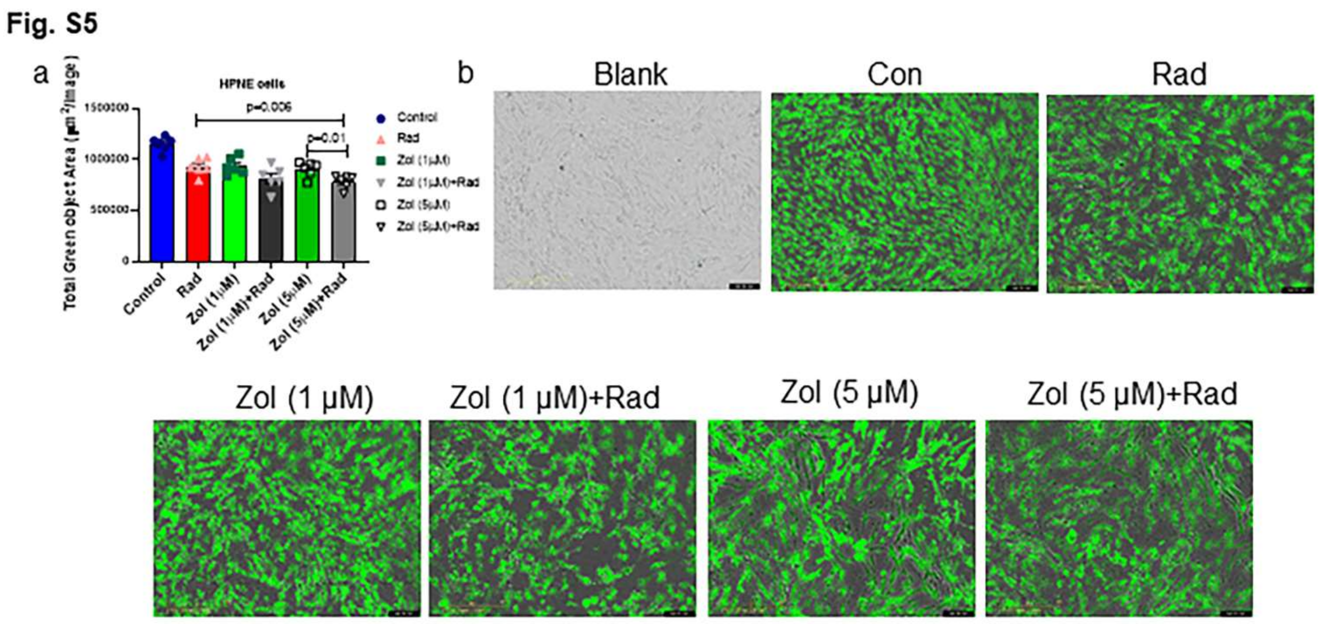

Fig. S5. Zol-mediated radiosensitivity of HPNE cells. HPNE cells (3500 cells/well) were seeded in 96 well plates and exposed with Zol and RT. After 48 h, cells were stained with calcein acetoxymethyl (calcein-AM) dye, and images were randomly captured using Incucyte live-cell analysis, and calcein fluorescence cells were quantified and analysed using the software attached to the live imaging system. High green fluorescence represents an increased percentage of live cells, and low green or red fluorescence indicates apoptotic or dead cells. (a) The bar graph shows a semi-quantitative analysis of the total number of live green positive cells per image under various treatments (Zol/RT) (Rad *vs.* Zol+Rad ($p=0.006$), Zol *vs.* Zol+Rad ($p=0.01$), $n=6$). Data are represented as mean±SEM, statistical analysis by student's t-test. (b) Representative calcein fluorescence positive cells under no calcein treatment (Blank), no drug treatment (control), radiation treatment (7 Gy), Zol alone treatment (1 µM and 5 µM) and Zol (1 µM and 5 µM) +Rad combination treatment.

Fig. S6

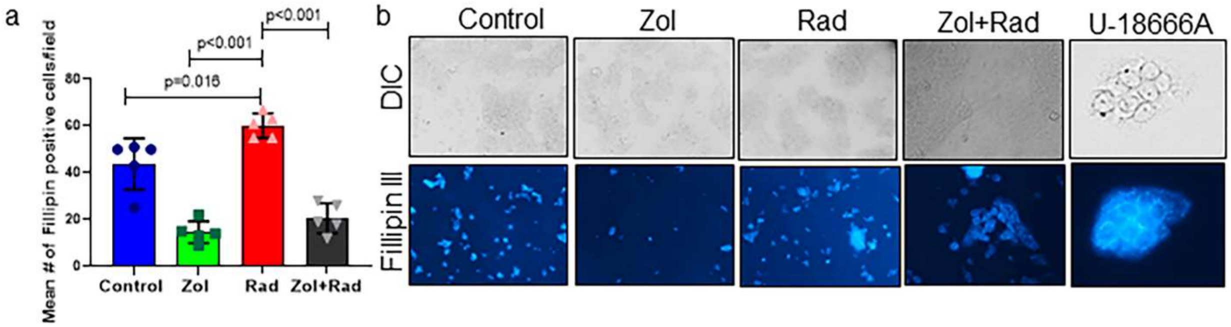

Fig. S6. Radiosensitization by Zol is associated with a reduction in cholesterol levels. Zol alone or with RT results in decreased cholesterol. (a) The bar graph represents the mean numbers of filipin cells positive in each group (Control *vs.* Rad (p=0.015), Zol *vs.* Rad (p<0.001), Rad *vs.* Zol+Rad (p<0.001), n=5). Statistical significance determined by student's t-test. (b) Representative brightfield light microscopy (upper panel) and fluorescence microscopy images (lower panel) depict filipin-positive cells upon treatment with/without Zol and RT in PDAC cells.

Fig. S7

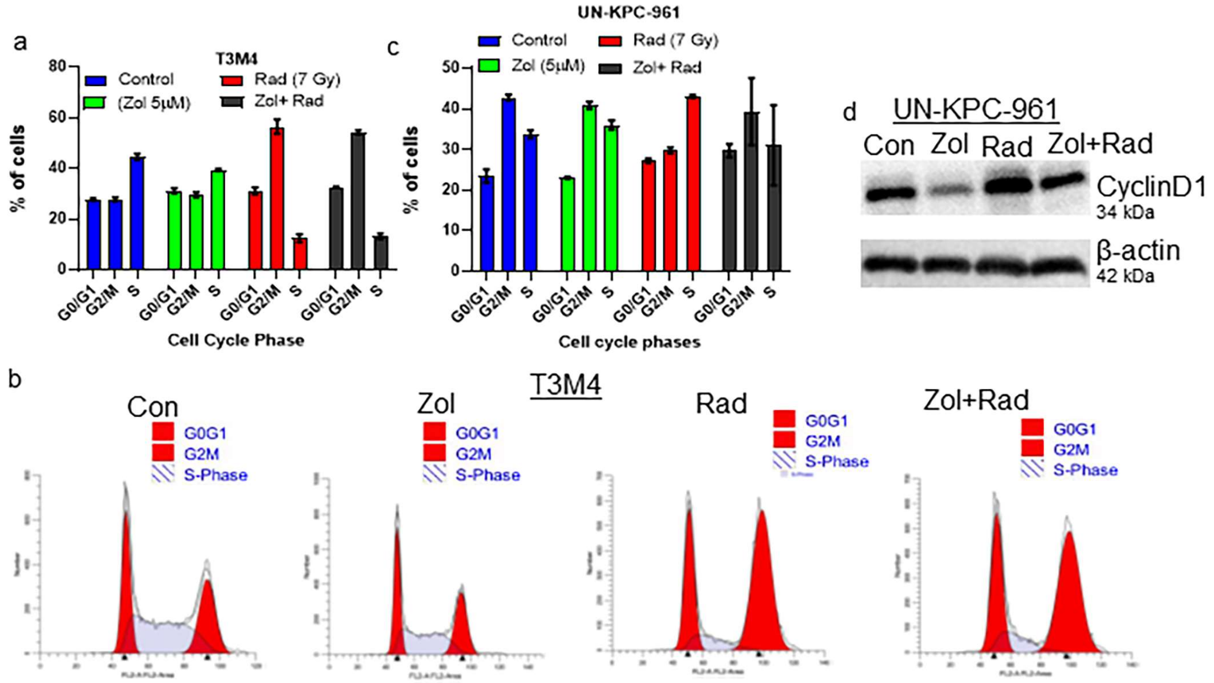

Fig. S7. The combination of Zol and RT treatment induces G0/G1 arrest by reducing G2/M and S-phase population in human T3M4 and mouse UN-KPC-961 PDAC cells. (a and c) Bar graphs showing the number of T3M4 and UN-KPC-961 PDAC cells accumulated in each Phase of the cell cycle following treatment with/without Zol and/or RT (n=3). (b) The histogram represents the percentage of cells distributed in each cell cycle phase in control and Zol- and/or RT-treated T3M4 PC cells. (d) Immunoblot analysis detecting Cyclin D1 protein expression in UN-KPC-961 PDAC cells after pre-treatment with Zol for 4 hrs followed by radiation treatment.

Fig. S8

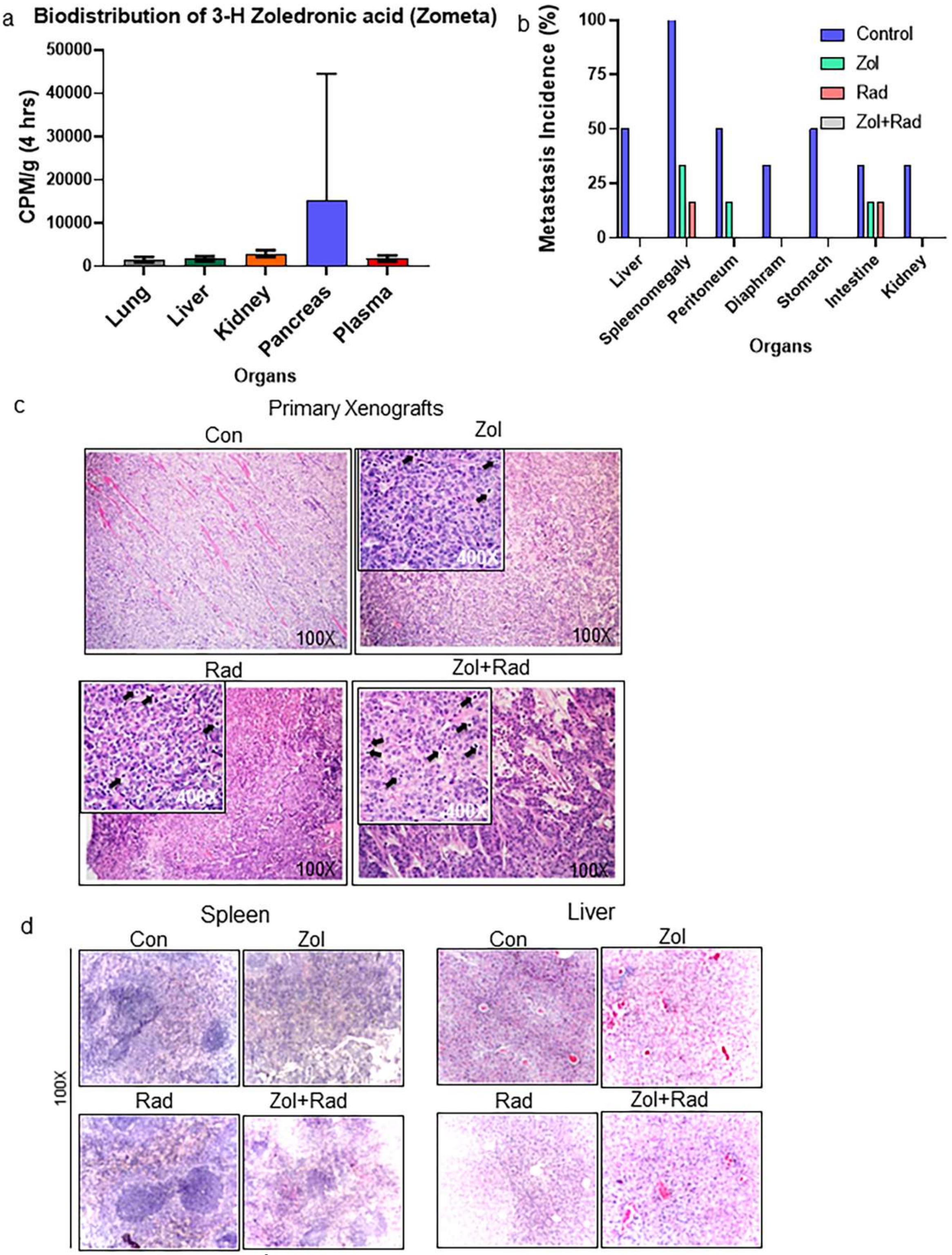

Fig. S8. Bio-distribution of ^{3}H-Zol in non-tumour-bearing mice. Radiolabeled Zol (0.5 microcuries/mouse) was administered through lateral tail vein injection. After 4 h treatment, the biodistribution of ^{3}H-Zol was examined in various vital organs, including the pancreas. (a) The bar graph shows the biodistribution of ^{3}H-Zol in various organs 4 h after administration. (b) Pre-treatment with Zol followed by RT inhibited PC tumour growth and

metastasis *in vivo*. The bar graph shows the number of animals with organ-specific metastasis detected in each treatment group (n=6). (c) Histopathological analysis of xenograft tissues pre-treated with Zol followed by RT. Hematoxylin and eosin (H&E) staining of xenograft tissues excised from athymic nude mice exposed to Zol and/or RT. Control xenograft tissue exhibited viable tumour cells, while cells treated with Zol alone showed evidence of apoptosis. Cells with RT displayed a moderate level of apoptosis with a smaller number of viable cells. Zol+RT-treated cells exhibited more apoptosis, debris, and less viability. Black arrows indicate apoptotic cells. (d) H&E staining of vital organs like the spleen and liver located close to the pancreas does not show signs of drug and radiation-related toxicities or damage.

Fig. S9

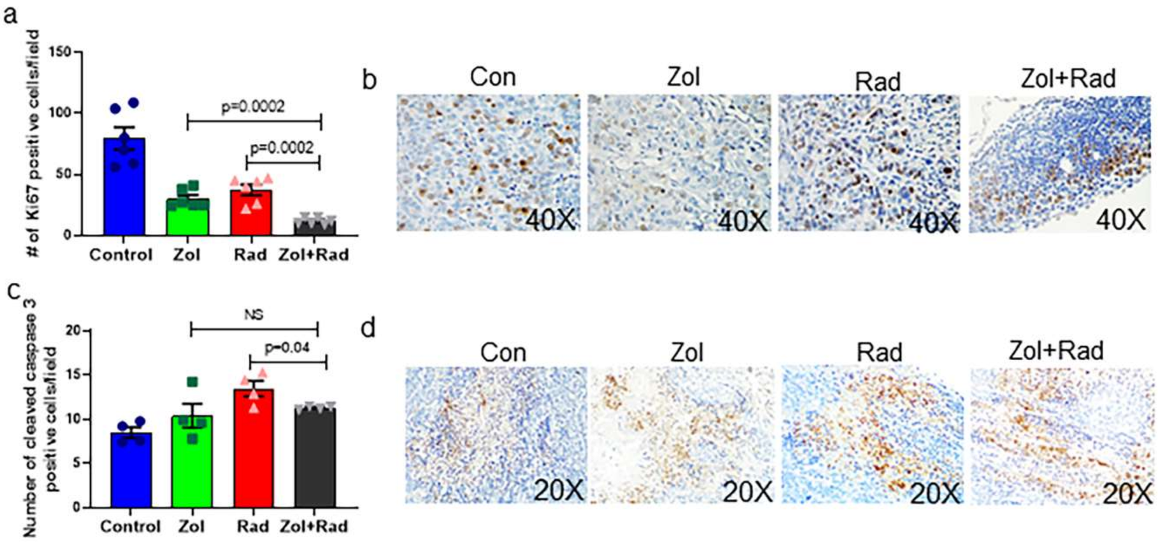

Fig. S9. Expression of Ki67 and cleaved caspase-3 in xenograft tissues. The histopathological differences between the treatment groups were assessed using proliferative marker Ki67 and apoptosis marker cleaved caspase-3 in xenograft tissues excised from *in vivo* mice model. (a and c) Scatter plot with bar graph represents the semi-quantitative analysis of xenograft tissue images showing positive expression for Ki67 (Zol *vs.* Zol+Rad (p=0.0002), Rad *vs.* Zol+Rad (p=0.0002), n=6) and cleaved caspase-3 (Zol *vs.* Zol+Rad (NS), Rad *vs.* Zol+Rad (p=0.04), n=4), Data shown as the number of positive cells (Ki67 and cleaved caspase-3) per field of the image of tissues in each group, significance determined using students t-test, Non- significant (NS). (b and d) Representative microscopic images from each group showed positive expression for Ki67 and cleaved caspase-3.

Fig. S10

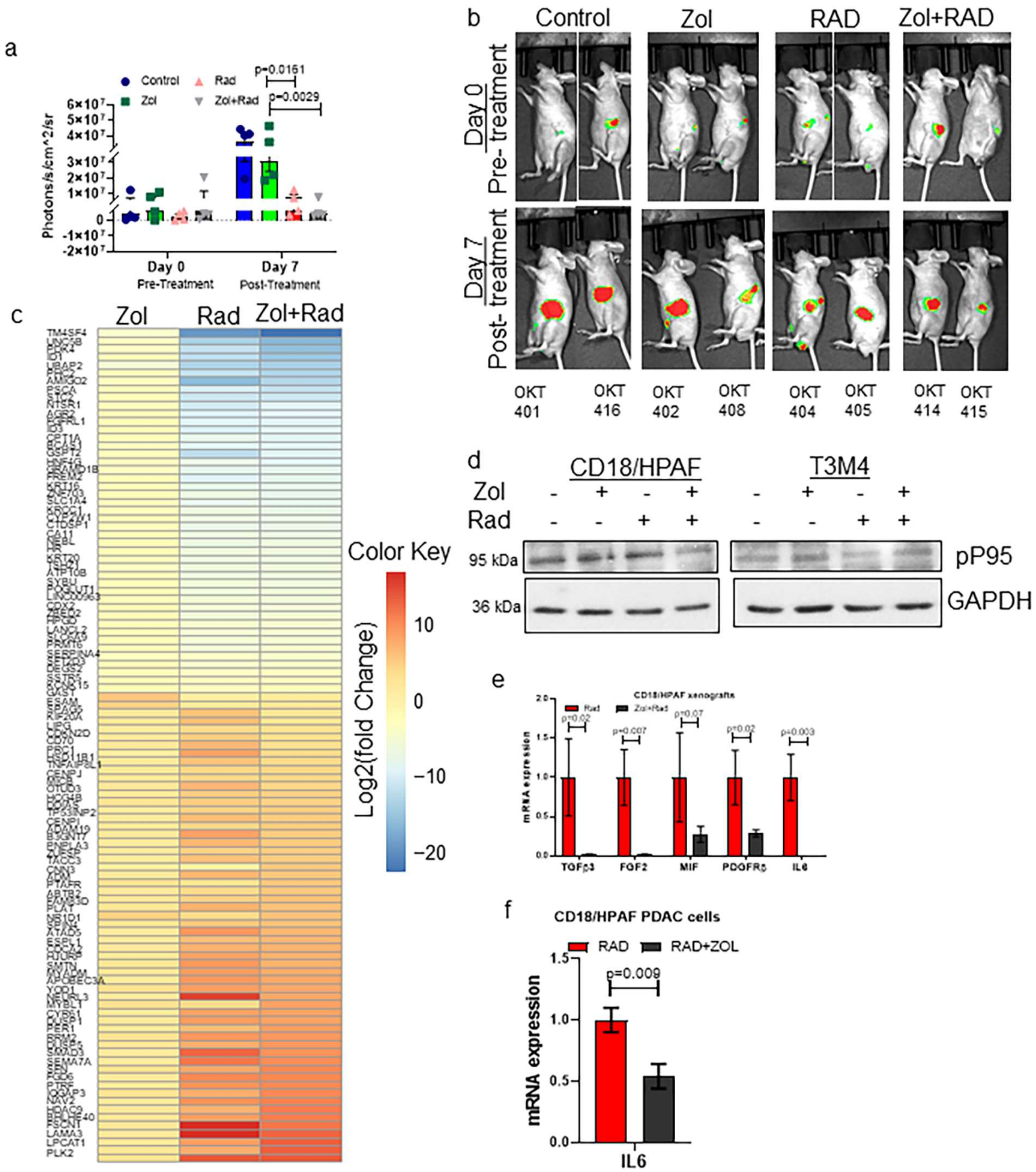

Fig. S10. Zol-mediated radiosensitization decreases xenograft growth via reducing DNA damage and repair signaling. (a) The bar graph represents the photon flux measured on the region of interest (ROI) emerging from bioluminescence signals from mice treated with/without Zol and/or RT at Day 0 (before treatment) and Day 7 (after RT) (Rad *vs.* Zol (p=0.0161), Zol *vs.* Zol+Rad (p=0.0029), n=4, ANOVA, Tukey's multiple comparison test). (b) Representative images of tumour-bearing athymic nude mice were administered luciferin and imaged using IVIS right before treatment (Day 0) and 7 days after initiation of treatment. Bioluminescence signals were

quantified and expressed as the mean of radiance (photons/s/cm^2/sr). (c) Heat map demonstrating hierarchical cluster analysis of DEG in the xenografts treated with Zol versus RT plus Zol. Genes upregulated in response to RT and Zol are marked in red, and downregulated genes are indicated in blue. (d and e) Zol inhibited DNA repair and damage signaling. (d) Immunoblot analysis detects pP95/NBS1 protein from CD18/HPAF and T3M4 PDAC cell lysates treated with Zol and RT alone and in combination. (e) RT-qPCR validation of TME-associated genes differentially responded against Zol+RT treatment identified through transcriptomic analysis of xenograft tissues. Bar graph showing genes (TGFβ3 ($p=0.02$), FGF2 ($p=0.007$), MIF ($p=0.07$), PDGFRβ ($p=0.02$), IL-6 ($p=0.003$), $n=3$) deregulated by Zol+RT combination relative to radiation alone treatment in xenograft tissues. (f) Bar graph indicates *IL6* ($p=0.009$, $n=3$) expression in CD18/HPAF PDAC cells pre-treated with Zol with/without radiation. *GAPDH* was used as an internal control. Data (e and f) are presented as the mean±standard deviation (SD) of biological replicates ($n=3$). P-values were determined using the student's t-test.

Fig. S11

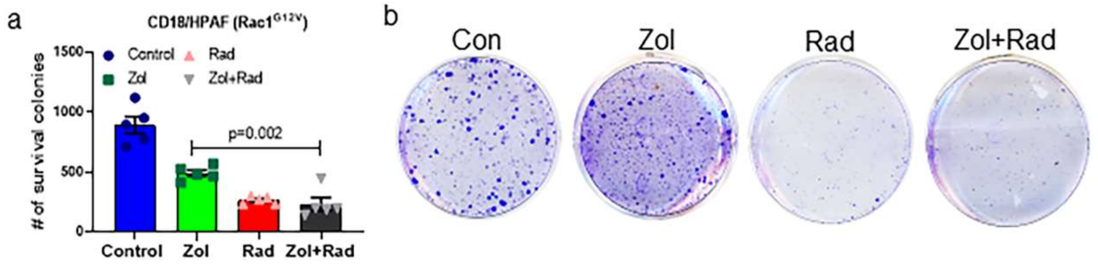

Fig. S11. Zol radiosensitizes constitutively active mutant Rac1^{G12V} overexpressed PDAC cells. CD18/HPAF PDAC cells were ectopically introduced with Rac1^{G12V} mutant construct and incubated with Zol (5µM) concentration and treated with radiation (7 Gy) and evaluated for their ability to reverse *in vitro* responses induced by Zol+RT. (a) Quantification of the number of survival colonies of CD18/HPAF Rac1^{G12V} overexpressed PDAC cells following treatment with Zol+RT (Zol *vs.* Zol+Rad ($p=0.002$), n=5). (b) Representative microscopic images of cell culture dishes (60 mm) with colonies formed against Zol+RT treatment. P-values were determined using the student's t-test.

Fig. S12

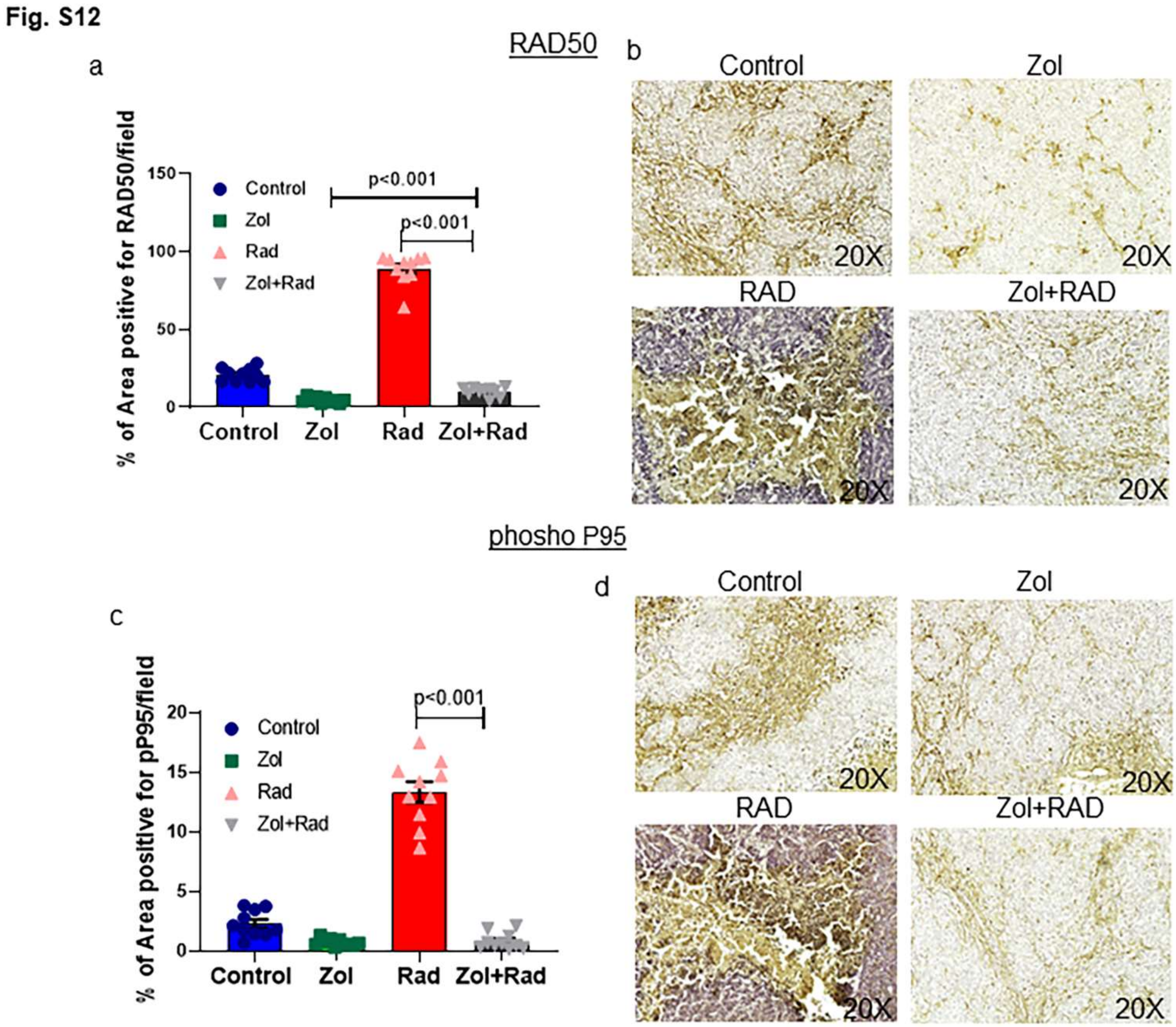

Fig. S12. Evaluation of DNA damage response proteins in xenograft tissues. Immunohistochemistry was performed on xenograft tissues using antibodies specific for Rad50 and pP95 proteins. (a and c) Scatter plot with a bar graph of the semi-quantitative evaluation of IHC staining intensity scores of RAD50 (Zol *vs.* Zol+Rad ($p<0.001$), Rad *vs.* Zol+Rad ($p<0.001$) (n=10/group), and phospho P95 (Rad *vs.* Zol+Rad ($p<0.001$)) staining distribution in xenograft tissues exposed to Zol and /or RT along with PBS treated control xenografts (n=10/group). p-values were determined using the student's t-test. (b and d) Representative light microscopic images show specific staining for Rad50 and pP95 in xenograft tumour tissues.

Fig. S13

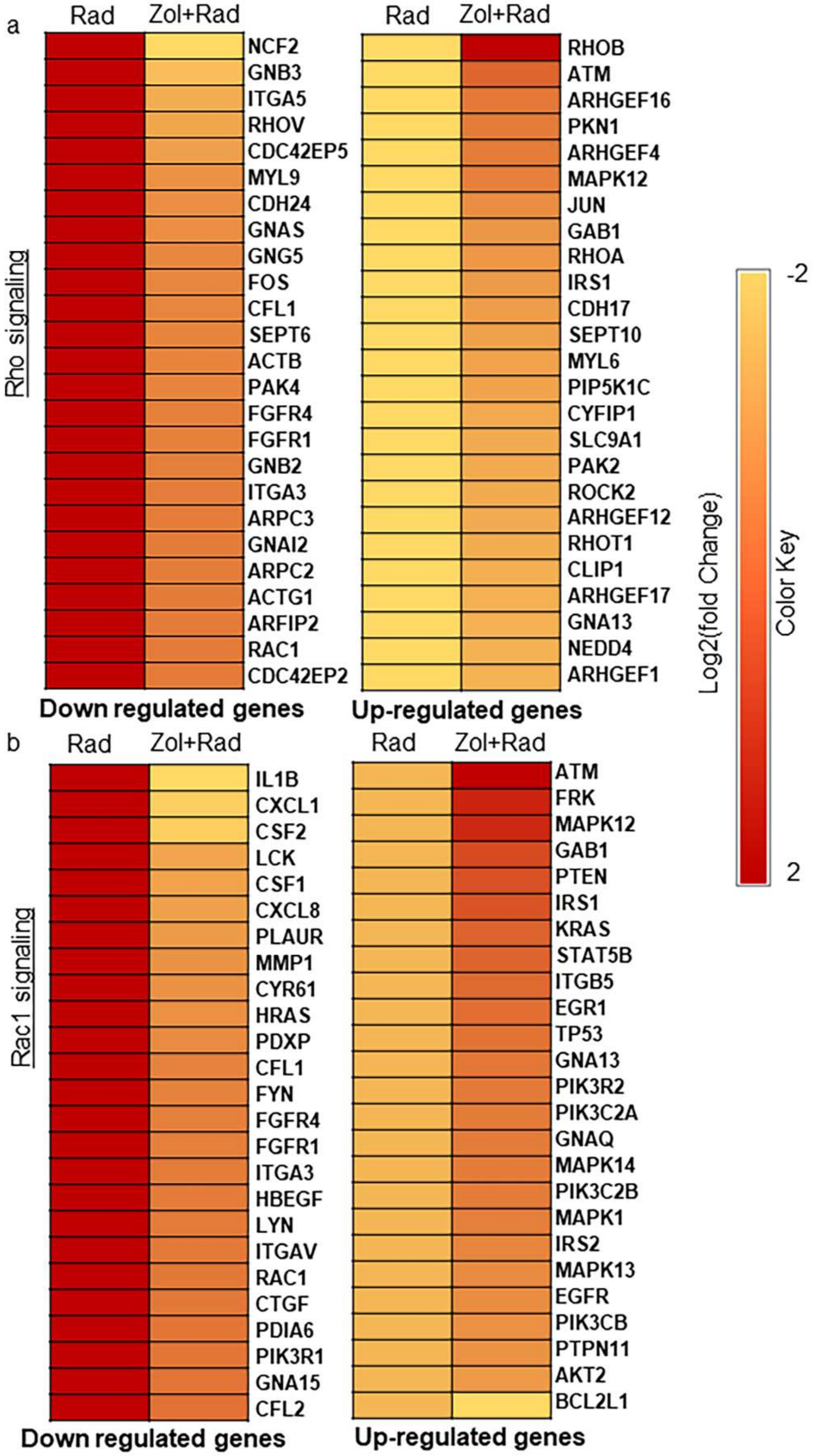

Fig. S13. Heat map showing the differential response of Rho (a) and Rac 1 (b) signaling in PC xenografts. Genes were aligned based on log2 fold change.

Fig. S14

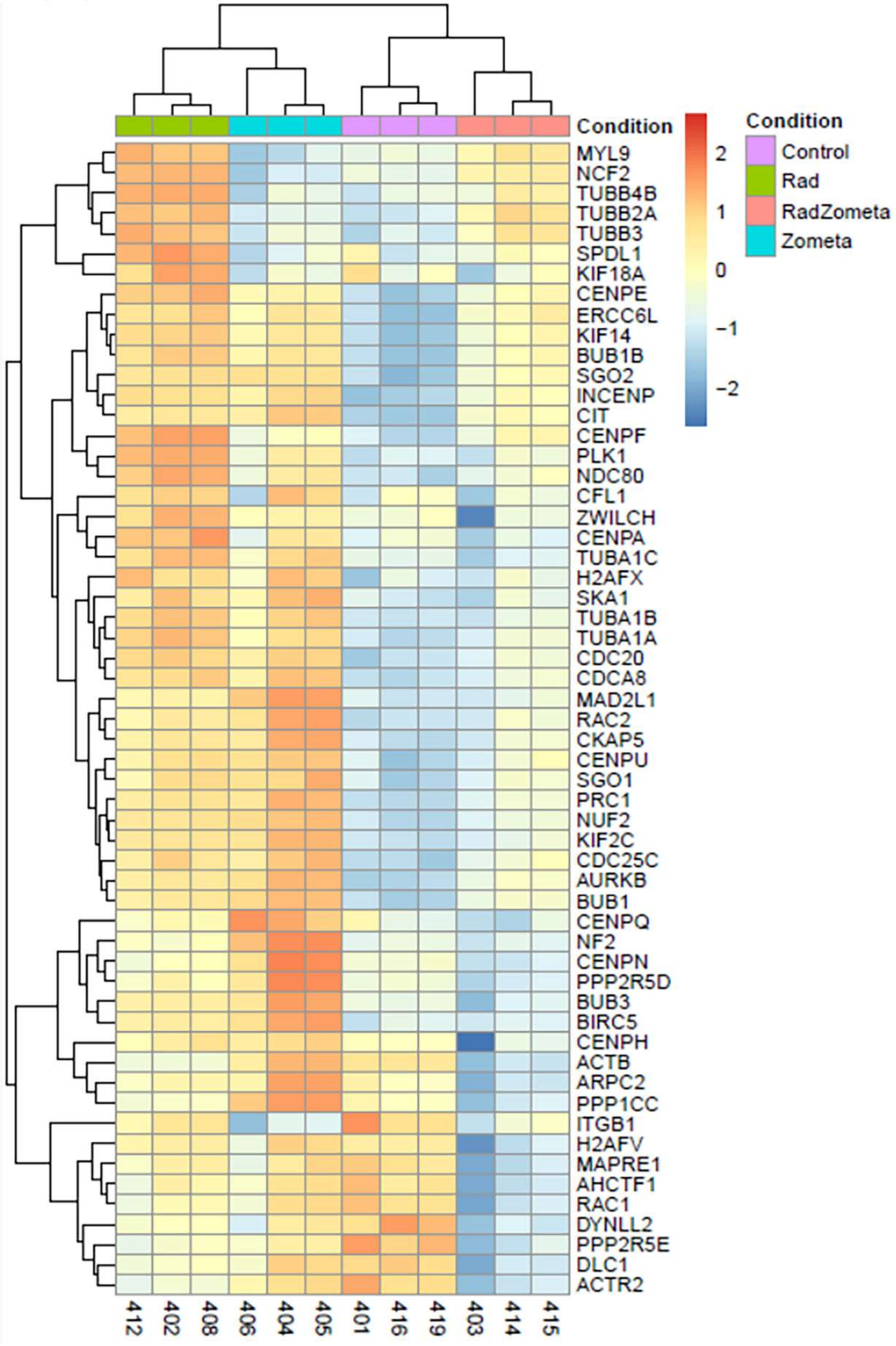

Fig. S14. Heat map showing the differential gene expression level of Rho GTPases family and subfamily genes under Zol and RT alone and in combination treatment in xenograft tissues.

Fig. S15

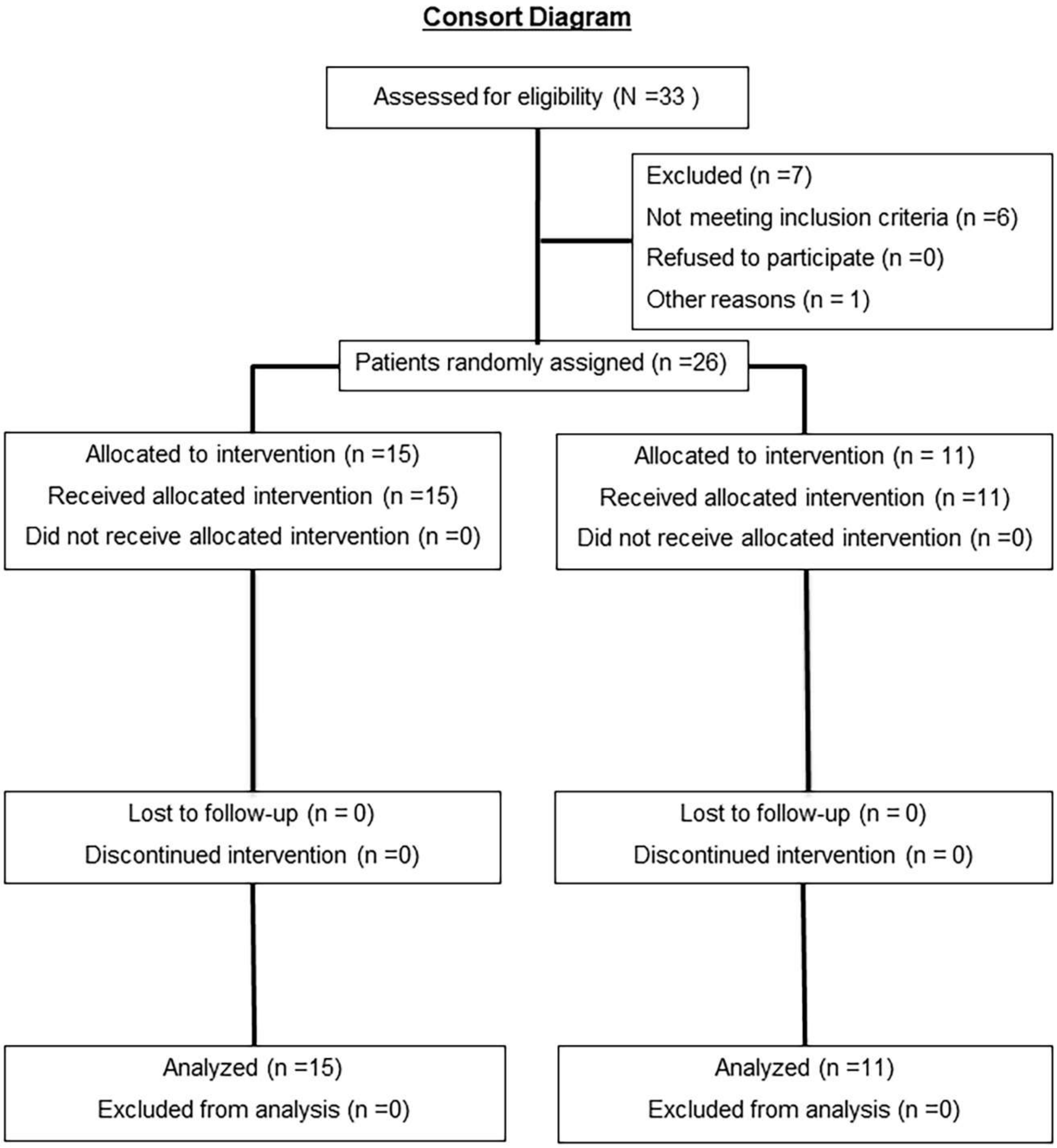

Fig. S15. Flow diagram describes the trail design, the number of patients recruited and allocated to study intervention, the number of patients followed-up, and patients considered for final analysis for the randomized phase I/II clinical study evaluating the safety and efficacy of Zol+RT.

IRB #: 552-16

A Randomized Phase II Study of the Efficacy and Safety of Hypofractionated Stereotactic Radiotherapy and 5FU or capecitabine with and without Zometa in Patients with Locally Advanced Pancreatic Adenocarcinoma.

***Principal Investigator:** Chi Lin, MD, PhD
987521 Nebraska Medical Center,
Omaha, NE 68198-7521
Phone: 402-552-3844
Fax: 402-552-3926
Email: Clin@unmc.edu

Secondary Investigators:
Lyudmyla Berim, MD
Jean L. Grem, MD
Kelsey A. Klute, MD
Quan P. Ly, MD
James C. Padussis, MD
James K. Schwarz, MD

Participating Investigators:
Chandrakanth Are, MD
Surinder Batra, PhD
Nicole de. Rosa, MD
Charles Enke, MD
Sarah P Thayer, MD
Robert B. Thompson, MD
Andrew Wahl, MD
Chi Zhang, MD
Weining Zhen, MD

Statistician:
Jane Meza, PhD
984375 Nebraska Medical Center,
Omaha, NE 68198-4375
Phone: 402-559-8407
Email: Jmeza@unmc.edu

Research Coordinators:
Amy Filler-Katz, RN,BSN,CCRP
986861 Nebraska Medical Center
Omaha, NE 68198-6861
Phone: 402-552-2790
Email: afillerkatz@unmc.edu

Brandi Booker,BSN
Phone: 402-559-8197

Study Product: Zometa
Version/Version Date 6.0 Dec. 10, 2018

SCHEMA
(Locally Advanced Pancreatic Adenocarcinoma)

Neoadjuvant chemotherapy
Folfirinox/nab-Paclitaxel plus Gemcitabine/others

↓

Post-chemotherapy restaging and Informed Consent
CBC, Chemistry, CA19-9, PET/CT, CTc/a/p

↓

Randomization: stratified by neoadjuvant chemotherapy regimens

↓

Week	Week1	Week1	Week1	Week1	Week1	Week1	Week1	Week 2-4	Week 5-9
Day -3 to -30	Day1	Day2	Day3	Day4	Day5				
Marker implant RT plan	RT	RT	RT	RT	RT			Post-RT break	Pre-op Re-stage w/wo Surgery
Planning CT & MRI	Capecitabine 650-825 mg/ m2 q12 hours (1650 mg/ m2/day) orally during radiotherapy starting the evening before day 1 of RT until the end of 4 weeks Or 5-FU infusion 225-250 mg/ m2/day IV continuous infusion beginning day 1 of RT until the end of 4 weeks								

OR

Week	Week1	Week1	Week1	Week1	Week1	Week1	Week1	Week 2-4	Week 5-9
Day -3 to -30	Day1	Day2	Day3	Day4	Day5				
Marker implant RT plan	*RT	RT	RT	RT	RT			Post-RT break	Pre-op Re-stage w/wo Surgery
Planning CT & MRI	*Zometa 4mg (IV infusion) 2-4 hours prior to radiation therapy on day 1 only.<br><br>*Capecitabine 650- 825 mg/ m2 q12 hours (per standard of care) orally during radiotherapy starting the evening before day 1 of RT until the end of 4 weeks Or 5-FU infusion 225-250 mg/ m2/day IV (per standard of care)continuous infusion beginning day 1 of RT until the end of 4 weeks								

TABLE OF CONTENTS

Page

SCHEMA .... ii/iii

1. OBJECTIVES 1

2. BACKGROUND 1

3. ELIGIBILITY CRITERIA 6

4. REGISTRATION PROCEDURES 7

5. TREATMENT PLAN 9

6. MEASUREMENT OF EFFECT 15

7. STUDY PARAMETERS 16

8. DRUG FORMULATION AND PROCUREMENT 16

9. TOXICITY REPORTING GUIDELINES 20

10. STATISTICAL CONSIDERATIONS 25

11. RECORDS TO BE KEPT 27

12. PATIENT CONSENT FORM STATEMENT 28

13. REFERENCES 30

14. DATA FORMS 34

APPENDICES

APPENDIX A
Criteria defining resectability status 35
APPENDIX B
Performance Status Criteria 36
APPENDIX C
Eligibility Criteria CRF 37
APPENDIX D
Specimen Requirement 40
APPENDIX E
NCI Common Toxicity Criteria Version 4.0 41
APPENDIX F
MEDWATCH 42

1.0 OBJECTIVES

1.1 Primary Objectives

1.1.1 To evaluate the efficacy of hypofractionated radiation therapy concurrently with Zometa and 5Fu or capcitabine.

1.2 Secondary Objectives

1.2.1 To examine the toxicity of Zometa while it is used concurrently with hypofractionated radiation therapy.

1.2.2 To evaluate local failure-free survival and overall survival, surgical resection rate and tumor response rate.

1.3 Correlative Studies

1.3.1 To quantify the amplitude of the expression of genes that are involved in cholesterol biosynthesis (ACAT2, DHCR7, ELFN2, FASN, SC4MOL, and SQLE) in pancreatic tumor tissue prior to and following the Zometa and radiation therapy if the pancreatic cancer tissue is available;

1.3.2 To measure Zometa pharmacokinetics at steady-state;

1.3.3 To evaluate tumor and organ motion with 4D CT and respiratory gating system and to evaluate the effect of tumor/organ motion on the dosimetry, local control and survival.

2.0 BACKGROUND

2.1 Neoadjuvant therapy for pancreatic adenocarcinoma

Pancreatic cancer, most commonly adenocarcinoma, is the fourth leading cause of cancer death in the United States, with an estimated 39,590 deaths in 2014 (1). The mainstay of management centers on surgical resection (if resectable); although there is no consensus, chemo(radio)therapy can be administered—if tolerated—before or after surgery (2). Although low (15% to 20%) resectability rates are associated with dismal survival, an estimated 80% to 85% of the patients recur after surgical resection, leading to a median survival of 20 to 24 months (3), and potentially even less depending on lymph nodal involvement or positive margins (4, 5). A large portion of the poor survival is associated with high and rapid distant (with or without locoregional) failure rates after surgery (6), even if adjuvant chemoradiotherapy (CRT) is administered (7). Although adjuvant therapies have been studied in greater detail than their neoadjuvant counterparts, the latter can similarly include chemotherapy alone or combined CRT. CRT can in turn be performed concurrently or sequentially. Concurrent CRT is preferred if feasible (2), but sequential regimens include induction chemotherapy followed by radiotherapy, potentially followed by chemotherapy again. The rationale for utilizing neoadjuvant therapy, commonly fluoropyrimidine-based or gemcitabine based chemotherapy or CRT (radiotherapy alone is rarely done), involves possibly down staging borderline resectable (BR) and unresectable (UR) patients, potentially making them resectable candidates. This also theoretically allows for R0 (negative margins) resection. A meta-analysis demonstrated that neoadjuvant CRT achieves R0 resection at a comparable frequency in initially resectable versus UR patients (8). Moreover, the 20% to 40% of the patients whose disease progresses while on CRT/chemotherapy are spared of unnecessary resection and associated morbidities (9–11). Because of surgical complications, early recurrence, and/or poor performance status, 15% to 30% of the resected patients may not be candidates for any adjuvant therapy (11–13). Postoperative surgical complications can delay adjuvant therapy, but neoadjuvant therapy is often completed by 90% to 100% of the patients versus only 62% for adjuvant therapy (14). It is important to note that there have been no studies to date indicating that neoadjuvant therapy, including chemotherapy versus CRT, carries survival benefits. Radiotherapy in the neoadjuvant setting provides several advantages over postoperative circumstances. Owing to a lack of tissue/architectural distortion, radiation may be better tolerated with a smaller treated volume, potentially causing reduced toxicity. In addition, surgical trauma can increase tissue hypoxia, which is a well-known cause of radioresistance, and in other cancers correlates with prognosis (15). A phase III trial comparing sequential neoadjuvant chemoradiotherapy (CRT) followed

by curative surgery to primary surgery alone for resectable, non-metastasized pancreatic adenocarcinoma is ongoing (16).

2.2. Rational for SRT and Dose

The recently completed phase II trial on neoadjuvant regimen in our institution includes several months of chemotherapy followed by 5 – 6 weeks of radiation therapy concurrent with radiation sensitizing chemotherapy, followed by a 4 - 6 weeks of post chemoradiation therapy break prior to surgery. Preliminary data from our institution indicates patients may develop disseminated disease during this lengthy period and thus become ineligible for surgery. Further, the chemoradiation is fairly debilitating. ECOG (17) conducted a phase II trial of preoperative conventional (50.4 Gy, 1.8 Gy/fraction) chemoradiation. The study showed that 51% of patients had hospital admission because of toxicities. The treatment-related toxicities are proportional to the irradiated volume and radiation dose. In M.D. Anderson, an accelerated radiotherapy schedule using 30 Gy in 10 fractions appeared to be more tolerable and equally effective (18, 19). A recent randomized trial (20) has compared preoperative short-course radiotherapy with preoperative conventionally fractionated chemoradiation for rectal cancer. The results showed no difference in actuarial 4-year overall survival (67.2% in the short-course group vs. 66.2% in the chemoradiation group, P = 0.960), disease-free survival (58.4% vs. 55.6%, P = 0.820), and crude incidence of local recurrence (9.0% vs. 14.2%, P = 0.170). The study also reported similar late toxicity (10.1% vs.7.1%, P = 0.360) and higher early radiation toxicity in the chemoradiation group (18.2% vs. 3.2%, P < 0.001). These data suggest the equivalence in efficacy between short course and long course neoadjuvant therapy Koong et al. (21) has conducted a phase I study of stereotactic radiosurgery (SRS) in patients with unresectable pancreatic cancer. Fifteen patients were treated at 3 dose levels (3 patients received 15 Gy, 5 patients received 20 Gy, and 7 patients received 25 Gy). No Grade 3 or higher acute GI toxicity was observed. In the 6 evaluable patients who received 25 Gy, the median survival was 8 months. All of patients had local control until death or progressed systemically as the site of first progression. This study suggests the feasibility of SRS in pancreatic cancer.

Following the methodology of Koong et al, one can apply the linear-quadratic formalism for radiation cell killing to "equate" schemes that vary the dose/fraction and number of fractions. This concept of biologically equivalent dose (BED) says that the total effect is given by:

$$(nd)\left\{1 + d \Big/ \frac{\alpha}{\beta}\right\}$$

where n is the # of fractions and d is the dose/fraction. The "alpha-beta ratio" characterizes the radiation response of a particular tissue; a higher value is indicative of a tissue that responds acutely to the effects of radiation. Due to their highly proliferative nature, most tumors fall into this category. Because prolonging the treatment time introduces a sparing (repair) effect in acutely responding tissues, there is significant motivation to deliver radiation in larger fractions over a shorter time.

Most recently, studies have shown that SBRT with sequential gemcitabine resulted in excellent local control of locally advanced pancreatic cancer with acceptable side effects (20, 21).

The duodenum is in closest proximity to the majority of the pancreatic head tumors, it is impossible to avoid treating this structure to a relatively high dose. Koong et al.'s data suggest that it is possible to irradiate a small volume of duodenum to a dose of 22.5 Gy in one fraction with acceptable toxicity.

While the dose-fractionation scheme employed by Koong et al resulted in no significant morbidity, we proposed a phase I trial to test hypofractionated stereotactic radiotherapy (SRT) and concurrent HIV protease inhibitor Nelfinavir (radiation sensitizer) as part of a neoadjuvant regimen in patients with locally advanced pancreatic cancer. We used more conservative starting dose in this study (5 Gy x 5) since a radiosensitizer (nelfinavir) was used to enhance the anti-tumor effect. Dose escalation of SRT/Nelfinavir

was as follows: 1) 5 Gy x 5/625 mg BID x 3 wk; 2) 5 Gy x 5/1250 mg BID x 3 wk; 3)6 Gy x 5/1250 mg BID x 3 wk; 4) 7 Gy x 5/1250 mg BID x 3 wk; 5) 7 Gy x 5/1250 mg BID x 5wk; and 6) 8 Gy x 5/1250 mg BID x 5 wk. Toxicity was assessed with CTCAE v3.

Forty-six patients have been enrolled since October, 2008 and tolerated up to the dose level 6. Median follow up is 13 months (95% CI: 3-36 months). During RT and 1.5 month post RT, ≥ grade 3 GI, hematologic and other toxicities were 2.6%, 2.6% and 13% respectively. Some of the side effects during this period were carried over from the period of induction chemotherapy. Twelve patients had resection. The resection rate is 27% (12/44.) Two patients are going to be evaluated for resection in the near future. During postoperative period, ≥ grade 3 GI, hematologic and other toxicities were 8%, 8% and 24%, respectively. The rate of ≥ grade 3 toxicity for patients at dose level 6 is (4/20) 20% which is acceptable per protocol. The protocol defined unacceptable toxicity is 2/3, 66% or 2/6, 33%. The median pathologic response scores for resected tumors were 4 (range: 0-9) with 1 complete response. The overall survival for patients with a resected tumor is significantly longer than the patients with an unresectable tumor (Log-Rank p=0.03) (see figure 1 below). Among patients with unresectable tumor, the overall survival of patients who received ≥ 35 Gy in 5 fractions is significant longer than those who received < 35 Gy in 5 fractions (Log-Rank p=0.002) (see figure 2 below). We concluded that SRT dose of 40 Gy in 5 fractions concurrent with Nelfinavir 1250 mg BID as part of neoadjuvant regimen is safe and has survival advantage. It is recommended to be the dose for the phase II trial. (59-65)

Figure 1

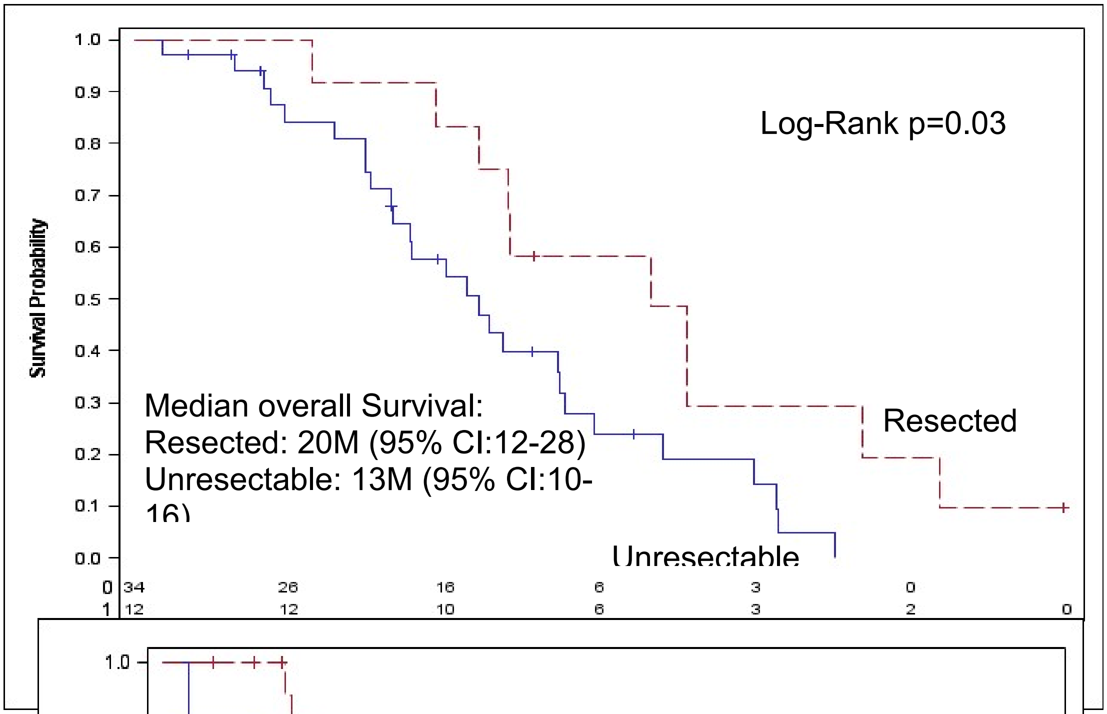

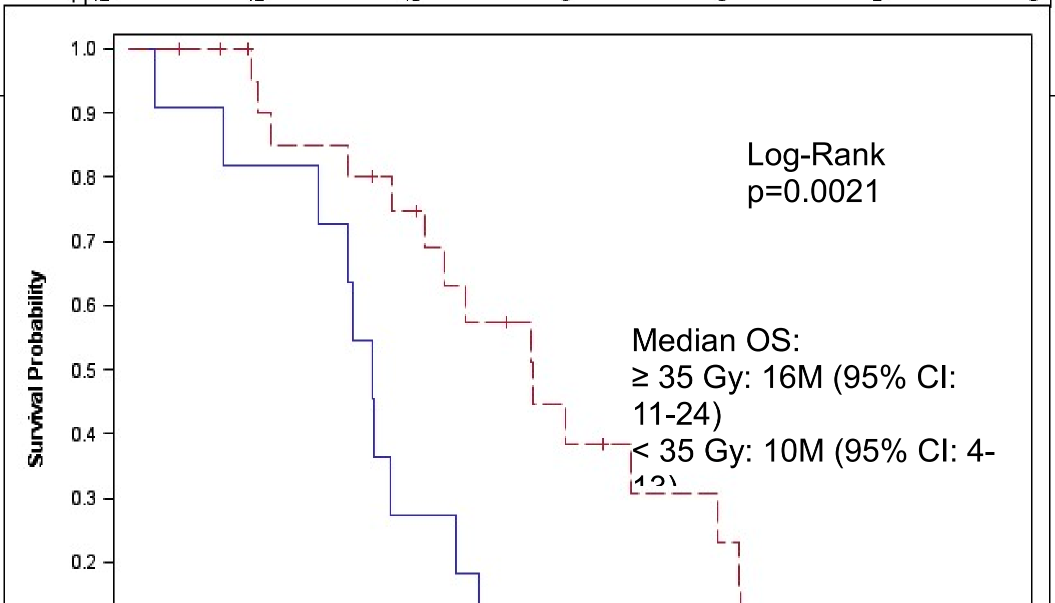

2.3 Rational for opening a second phase II trial on locally advanced pancreatic adenocarcinoma patients.

The neoadjuvant chemoradiation therapy in the current ongoing phase II trial consists of 3 cycles (9 weeks) of gemcitabine and 5 FU followed by SBRT. A phase III trial published in 2011 NEJM revealed that FOLFIRINOX was associated with a survival advantage as compared with gemcitabine for patients with metastatic pancreatic cancer (22). Another phase III trial published in 2013 NEJM showed that nab-paclitaxel plus gemcitabine significantly improved overall survival, progression-free survival, and response rate in patients with metastatic pancreatic adenocarcinoma when compared to gemcitabine alone (23). Since the publications of the above mentioned two trials, a large number of patients who appeared in my clinic have received one of the above mentioned two regimens. These patients will not be eligible for the current ongoing 441-13 phase II clinical trial which will only accrue chemotherapy-naïve patients. However, these patients will be eligible for this trial which allows patients to receive these more popular and more effective chemotherapy prior to be accrued into the trial.

2.4 Rational for using real-time motion tracking during SRT

The issue of respiratory motion has long been recognized as a major limitation in the management of radiotherapy patients. For patients receiving stereotactic body radiation therapy (SBRT), the dose per fraction is large. Therefore, the high-dose tumor volume has to be compact. We elect not to irradiate the lymphatic drainage area prophylactically since studies have shown that regional nodal failure is only 7-10% while local and distant failure has reached 50% (24, 25). With such a compact volume, tumor position must be accurately assessed throughout the radiation treatment, especially for the pancreatic tumor that moves with respiration.

Minimizing the impact of respiratory motion is essential in order to achieve further gains in the treatment of pancreatic disease. In this study, we will apply advanced imaging and delivery technology to provide added confidence in imaging and targeting. All patients will undergo a planning 4D CT. CT images will be reconstructed as a function of respiratory phases. End exhale images will be used for planning purposes, as this has been shown to be the most reproducible phase of respiration with the longest duration (26-32).

The linear accelerators with SRT capability will be used to deliver SRT. For patients consented prior to diagnostic laparoscopy, Calypso Beacon markers will be placed during the laparoscopy. The Calypso System allows for real-time tracking of tumors during cancer radiation treatment. Calypso continuously tracks the position of the tumor using an innovative technology known as "GPS for the Body®." This tracking technology is guided by two to three tiny Beacon transponders — wireless devices that each are about the size of a grain of rice. The transponders transmit safe radiofrequency waves that provide sub-millimeter, real-time information about the position and movement of the tumor.

For patients who consent after diagnostic laparoscopy, Calypso Beacon markers will be placed by Interventional Radiology. For those patients who cannot use Calypso system, radio-opaque gold markers

will be placed. The Exac Trac incorporates stereotactic x-ray capabilities for verifying target position. For soft tissue targets the system is designed to be used with radio-opaque gold markers implanted near the target. These markers are implanted prior to CT imaging and treatment planning, and should be placed close enough to the target anatomy so that they can be observed within the field of view of the x-ray localization system at the time of treatment The use of implanted makers for radiotherapy localization has been described for a number of tumor sites, including prostate (33, 34), liver (35), lung (36, 37) and pancreas (38).

When the Artiste and Truebeam linear accelerators are used to deliver radiation therapy, the maximum dimension of the tumor can be up to 10 cm. CT on rail (Artiste) or KV cone beam CT (Truebeam) together with Calypso system will be used for image-guided radiation therapy.

2.5 Rational for using Zometa as a radiation sensitizer

Recent work in radiobiology indicates that alterations in specific biochemical pathways are largely responsible for tumor radioresistance (39-43); however, these pathways cannot be generalized across cancers particularly radioresistant pancreatic cancer. Specific cellular process are considered likely to be consistently involved in decreased tumor radio-response such as DNA damage sensing and repair, inhibition of apoptosis, autophagy, and cell cycle checkpoint regulation (43-45). Unfortunately, biochemical pathways associated with these processes are too vast and complex to allow a reasonable likelihood for success in implicating potential targets with clinically relevant radioresponse through individual testing (43, 46, 47). Further, current methods for *in vivo* validation of promising *in vitro* radiation results are relegated to subcutaneous transplant murine models that fail to recapitulate the complexity of pancreatic cancer microenvironment. Pancreatic cancer is highly desmoplastic and tumor stoma has significant potential to alter cellular response to both internal and external stimuli, thereby preventing analysis of a potential radio-sensitizer's success in a disease-relevant context prior to human testing. These points are well illustrated by the fact that, despite decades of research, to date fewer than 10 genes have been shown to contribute to pancreatic cancer radioresistance (42, 48, 49), with none resulting in clinically efficacious radio-sensitizers.

In our previous studies (50), we identified novel pathways affecting radiation response through an unbiased, global analytical approach for natural and radioselected radiation resistance in PC cells. Interestingly, a total of 11 genes (FDPS, ACAT2, AG2, CLDN7, DHCR7, ELFN2, FASN, SC4MOL, SIX6, SLC12A2, and SQLE) were found to be consistently associated with radio-resistance in several cell lines as confirmed using both genomic and proteomic approaches. Importantly, pathway analysis revealed that the majority of these genes are involved in cholesterol biosynthesis (ACAT2, DHCR7, ELFN2, FASN, SC4MOL, and SQLE). Inhibition of cholesterol biosynthesis by Zometa imparted radio-sensitization to a panel of radio-resistant pancreatic cancer cells from both murine and human origins. Our data showed that pharmacologic inhibition of FDPS by Zometa radiosensitised pancreatic cancer cell lines, with a radiation enhancement ratio between 1.26 and 1.5. Further, Zometa treatment resulted in radiosensitisation of pancreatic cancer in an allograft mouse model.

The dose of Zometa for this study was chosen because it is within the range of Zometa used in clinical trials (4–16 mg) and because it is well below the highest reported non-lethal single dose (10 mg kg_1) in mice (Zometa package insert, 2006). Possible reasons for not reaching significance in the in vivo studies could be single dose administration of Zometa to more accurately reflect clinical dosing, use of a highly radioresistant cell line, or a sample size of mice too small to reach significance.[50]

Zometa has been used continuously in patients with bone metastases. There are reports of patients on Zometa who have received radiation therapy; no increase in side effects from the radiation have been reported (51, 52). An RTOG published study(RTOG 0518) using RT and Zometa (4 mg) concurrently

without any significant toxicity (73).

In summary, there is clearly strong rationale to proceed with a clinical trial of Zometa and radiation in pancreatic cancer: (a) Preclinical work demonstrates inhibition of cholesterol biosynthesis results in radio-sensitization in pancreatic cancer cells. (d) Zometa has been safely administered to cancer patients over the last decade with minimal side effects.

3.0 ELIGIBILITY CRITERIA

3.1 Inclusion Criteria

3.1.1 Pathologically confirmed adenocarcinoma of the pancreas. Patients with either initially diagnosed or recurrent locally advanced disease. The maximum dimension of the treatment target must be ≤ 10 cm. Locally advanced disease defined as: T 1-2N+MO or T3-4 NxMo, or borderline resectable and unresectable adenocarcinoma without distant metastatic disease or resectable T3-4 NxMo disease or M1 with controlled distant disease.

3.1.2 Patients with inoperable conditions with resectable disease (T1-2NoMo)

3.1.3 Age: Patients must be 19 years of age or older. (This is the age of consent in Nebraska. Pancreatic cancer does not occur in the pediatric age group.)

3.1.4 Karnofsky Performance Status of 60% or better. (Appendix B)

3.1.5 Patients who received recent chemotherapy for pancreatic cancer are eligible. Patients who received chemotherapy > 5 years ago for malignancies other than pancreatic cancer are also eligible, provided that chemotherapy was completed > 5 years ago and that there is no evidence of the second malignancy at the time of study entry.

3.1.6 Patients who received radiation therapy > 5 years ago for malignancies other than pancreatic cancer and whose radiation therapy field is not overlapping with the 20% isodose line of current radiation field are eligible, provided that radiation therapy was completed > 5 years ago and that there is no evidence of the second malignancy at the time of study entry.

3.1.7 All malignant disease must be able to be encompassed within a single irradiation field.

3.1.8 All patients must have radiographically assessable disease

3.1.9 Patients must have a ANC greater than or equal to 1500/µL and platelet count greater than or equal to 100,000/µL

3.1.10 Patients must have a serum creatinine less than or equal to 2.0 mg/dL and total bilirubin less than or equal to 2.0 mg/dL in the absence of biliary obstruction. If the patient has biliary obstruction, biliary decompression will be required. Either endoscopic placement of a biliary stent or percutaneous transhepatic drainage is acceptable. Once biliary drainage has been established, institution of protocol therapy may proceed when the total bilirubin falls to 4.0 mg/dL or lower.)

3.1.11 Patients must have a Calculated Creatinine Clearance of ≥35 .

3.1.12 The patient must be aware of the neoplastic nature of his/her disease and willingly provide written, informed consent after being informed of the procedure to be followed, the experimental nature of the therapy, alternatives, potential benefits, side-effects, risks, and discomforts.

3.2 Exclusion criteria

3.2.1 Patients with a known allergy to Zometa or to antiemetics appropriate for administration in conjunction with protocol-directed therapy.

3.2.2 Uncontrolled inter-current illness including, but not limited to ongoing or active infection requiring intravenous antibiotics, symptomatic congestive heart failure, unstable angina pectoris, or serious, uncontrolled cardiac arrhythmia, that might jeopardize the ability of the patient to receive the therapy program outlined in this protocol with reasonable safety.

3.2.3 Pregnant and nursing women are excluded from this study because the effects of Zometa on a nursing infant are not fully known and abdominal radiation therapy all have the potential for teratogenic or abortifacient effects.

3.2.4 Patients with prior malignancy will be excluded except for adequately treated basal cell or squamous cell skin cancer, adequately treated noninvasive carcinomas, or other cancers from which the patient has been disease-free for at least 5 years.

3.2.5 Patients with active duodenal ulcer or bleeding or history of a gastrointestinal fistula or perforation or other significant bowel problems (severe nausea, vomiting, inflammatory bowel disease and significant bowel resection).

3.2.6 Patients with known HIV infection, or hepatic insufficiency.

3.2.7 Patients may not be receiving or have received Zometa during/or within 3 weeks prior to treatment with Zometa.

3.3 Inclusion of Women and Minorities

Both men and women and members of all races and ethnic groups are eligible for this trial.

4.0 REGISTRATION PROCEDURES

All patients with pancreatic cancer or suspicious pancreatic masses referred to the Nebraska Medical Center (NMC) / UNMC are evaluated in a multidisciplinary team conference. On initial presentation, a history and physical examination are performed, laboratory data obtained, and performance status is assessed. Imaging studies obtained include a high-resolution multi-detector computed tomography (CT) of the chest,abdomen and pelvis. Further imaging studies will be obtained as clinically indicated. Any pathologic specimens obtained at referring institutions are reviewed for accuracy. Patients with suspicious pancreatic masses will require pancreatic biopsy for confirmation of malignancy. Biopsy techniques available include percutaneous, endoscopic ultrasound guidance, and laparoscopic. Patients without evidence of metastatic disease on imaging studies will be evaluated for potential resectability (NCCN Guidelines- Appendix A).

Imaging studies available for use in defining resectability include CT scan (MRI scan will be done if CT scan is not sufficient for evaluating the resectability), endoscopic ultrasound, and laparoscopic ultrasound. The CT scan obtained is an imaging protocol specifically designed to evaluate the pancreas using a triple phase contrast scan with thin (3 mm) cross-sectional images with 3-dimensional reconstruction availability. CT mesenteric angiography will be obtained when clinically indicated. Endoscopic ultrasound and laparoscopic ultrasound will be utilized when additional information regarding tumor relationship with surrounding vascular structures, or when diagnostic tissue samples are required.

Patients with resectable or borderline resectable pancreatic adenocarcinoma who meet the eligibility criteria will be offered participation in the treatment portion of this trial. More subjects will participate in this pre-therapy evaluation phase than will ultimately be found to be suitable candidates for the chemotherapy/radiation therapy.

Patients without peritoneal disease will have CT-guided placement of Calypso Beacons or markers around the pancreatic cancer. Patients who undergo a laparoscopy at an outside facility or have a laparoscopy done prior to consent will have two Calypso Beacons or markers placed by Interventional Radiology prior to Radiation Therapy.

The standard of care outside clinical trials setting for patients with localized or locally advanced pancreatic cancer with resectable disease is surgery followed by adjuvant therapy. In patients with locally advanced pancreatic adenocarcinoma that is potentially resectable (provided an antitumor

response is obtained) or unresectable, palliative chemotherapy alone or in combination with palliative radiation therapy is considered the standard of care.

NOTE: Before patients are enrolled into the treatment portion of the study, an eligibility checklist (Appendix C) must be completed to verify the subject meets the eligibility and may be used as source documentation if it has been reviewed, signed, and dated prior to registration by the treating physician.

Some insurance carrier's may decline to cover the costs of usual medical care if the patient is participating in a clinical trial. The patient will be provided assistance by the research nurse coordinator in determining if the insurance carrier will decline coverage. Insurance carriers may or may not pay for study related expenses. The patient can then decide if they wish to participate.

4.1 Eligibility Verification/Registration

Before patients are enrolled into the study, an eligibility checklist (Appendix C) must be completed to verify the subject meets the eligibility criteria. Date of enrollment is defined as the date of the start of study treatment / first protocol related intervention. The eligibility check list will be maintained in the study file.

Study personnel will provide the UNMC Fred & Pamela Buffett Cancer Center PRMS office (ZIP 6805) a copy of the signed and dated consent form for each UNMC subject registered to the protocol within 7 days that includes the following information:

- Protocol Number
- Patient Identification: Patient's name NMC medical record number
- Patient demographics: gender, birth date (mm/dd/yyyy), race, ethnicity

5.0 TREATMENT PLAN

5.1 Marker implantation Standard of Care (SOC) (day -3 to day -30)

Patients without peritoneal disease will have CT-guided placement of Calypso Beacons or markers around the pancreatic cancer.

Patients who undergo a laparoscopy at an outside facility or have a laparoscopy done prior to consent, will have two Calypso Beacons or markers placed by Interventional Radiology prior to Radiation Therapy. The Calypso Beacons or markers will be placed by Interventional Radiology during chemotherapy cycles. Prophylactic antibiotics will be given to prevent infection. Calypso Beacons or marker position and geometry will be documented on a pre-RT staging CT scan. For some patients, either Calypso Beacons or markers may not be needed. This decision is made by the radiation oncologist.

CT scan of the chest will be performed for restaging; **an MRI scan of the abdomen** will be done for staging and SRT planning. Patients with no metastasis will proceed with SRT. For patients with recurrent disease, patients should have no other disease.

Week -1-3: Treatment planning for Stereotactic Radiotherapy (SRT).

5.2 Administration of Zometa (Res) and Chemotherapy

Week 1(day one radiation therapy): 4 mg as a single-use intravenous infusion over no less than 15 minutes. Administer with oral calcium supplements of 500 mg and 400 international units of vitamin D daily for 30 days from the day of administration of Zometa.

The goal dose of zoledronic acid is 4 mg, but the selection of dose is determined based on calculated creatinine clearance at baseline, using the formula below:

Serum creatinine will be measured within 7 days prior to administration of study drug. The goal dose of zoledronic acid (Zometa®) is 4 mg, but the selection of dose is determined based on calculated serum creatinine clearance (see parameters below) estimated by Cockcroft-Gault formula:
CrCl male = [(140 – age) x (wt in kg)]
[(sCR) x (72)]
CrCl female = 0.85 x (CrCl male)

The laboratory result must be available prior to administration of the dose of zoledronic acid

**Dose Determination Based on Calculated Creatinine Clearance**	
**Creatinine Clearance**	**Zoledronic Acid Dose**
> 60 ml/min	4 mg
50-60 ml/min	3.5 mg
40-49 ml/min	3.3 mg
35-39 ml/min	3 mg

Treatment will be administered on an outpatient basis. Reported adverse events and potential risks are described in Section 8. Appropriate dose modifications are described in Section 5.10. No investigational or commercial agents or therapies other than those described in this protocol may be administered with the intent to treat the patient's malignancy.

5.2.1 **Capecitabine 650- 825 mg/ m2 q12 hours (per standard of care) orally during radiotherapy starting the evening before day 1 of RT until the end of 4 weeks**

Or

5-FU infusion 225-250 mg/ m2/day IV (per standard of care) infusion beginning day 1 of RT until the end of 4 weeks .

5.3 Stereotactic Radiotherapy (SRT) (SOC)

Week 1: Start **Stereotactic Radiotherapy** (SRT) the total dose of 40 Gy will be delivered in 5 Fractions over 5 consecutive days for 1 week (details of SRT are provided in Section 5.9).

5.4 Pre-Surgery (SOC)

week 5-9: **Restaging**, a **CT scan of the chest and abdomen** will be performed for restaging 4-8 weeks after completing SRT to assess disease response. An **MRI scan** may be performed if CT scan is not sufficient for assessing the resectability.

If surgical resection is not possible and the clinician determines that chemotherapy should be resumed, patients will complete study and will be evaluated for further chemotherapy.

5.5 Surgery (if resectable or potentially resectable)

Week 6-9: Patients without metastasis and with resectable disease will undergo definitive surgery. If no contraindication for surgical resection is identified, resection will be performed 6-8 weeks after completing SRT. At the time of surgical resection, an extensive examination of the abdomen will be performed to exclude the presence of metastatic disease. All operations will be performed with curative intent with resection of all gross tumor (ie R0 [negative margins]. Resection of adjacent involved organs or vascular structures will be performed as clinically indicated.

Standardized histopathologic analysis of resected specimens will be performed. Margins to be evaluated include: common bile duct, pancreatic, retroperitoneal (tissue between superior mesenteric artery and duodenum), as well as tissue along the superior mesenteric vein and artery. Examination of regional lymphatics will be performed according to standard pathology techniques.

5.6 Details of SRT: The SRT treatment will consist of image-guided radiotherapy.

5.6.1 Patient's positioning: The treatment position of the patient is supine, with the arms placed above the head. The immobilization device (thermoplastic mask) will include total body to make sure that the patients' position is the same during planning, simulation and treatment.

5.6.2 Patient data acquisition: Treatment planning 4D CT scans are required to define tumor, clinical, and planning target volumes. The treatment planning 4D CT scan with IV contrast will be acquired with the patient in the same position and immobilization device as for treatment. All tissues to be irradiated will be included in the CT scan. CT scan thickness should be ≤ 3 mm through the region that contains the primary target volumes. Conventional MRI scans (T1 and T2) may be included to assist in definition of target volumes. FDG PET-CT information may be included in the treatment planning; no extra scans will be performed for study purposes. The GTV, CTV and PTV, and normal tissues (OAR) must be outlined on all CT slices in which the structures exist.

5.6.3 Volumes: The definition of volumes will be in accordance with the 1993 ICRU Report #50:

5.6.4 Prescribing, Recording and Reporting Photon Beam Therapy. The **Gross Tumor Volume (GTV)** is defined as all known gross disease determined from CT, clinical information, endoscopic findings, FDG PET-CT and/or conventional MRI. The **Integrated Tumor Volume based on CT/MRI/PET (GTV_{fusion})** is defined as gross disease on the free breathing CT scan, MRI scan and FDG-PET scan. These scans will be correlated by imaging fusion technique. The volume will be delineated by the treating physician on the above scans separately. The GTV_{CT}, GTV_{MRI} and GTV_{PET} (if done) will be eventually fused together to generate GTV_{fusion}. The integrated GTV is created with 4D CT information to compensate internal organ motion. Patients who have the maximal dimension of the GTV_{fusion} > 8 cm will not be eligible for the study. The **Clinical Target Volume (CTV)** is defined as the GTVs plus areas considered to contain potential microscopic disease. In this study, the CTV is defined as areas around GTVs (i.e. both the primary tumor and the lymph nodes containing clinical or radiographic evidence of metastases) and areas around SMA. The **Planning Target Volume (PTV)** will provide a margin around integrated CTV to compensate for the variability of treatment set-up and internal organ motion. The integrated GTV will receive 8 Gy x 5, The CTV will received 6 Gy x 5, and the PTV will receive 5 Gy x 5. If the GTV or CTV or PTV overlaps with small bowel or stomach, then the overlapping area will receive 4 Gy x 5.

5.6.5 Organs at Risk (OAR) The normal tissue volumes to be contoured include:the skin surface (the tissue within the skin surface and outside all other critical normal structures and PTVs is designated as unspecified tissue), spinal cord (spinal cord contours will be defined at least 5 mm larger in the radial dimension than the spinal cord itself, i.e. the cord diameter on any given slice will be 10 mm larger than the cord itself), duodenum, stomach, liver, right kidney, left kidney, small bowels exclude duodenum, spleen

5.6.6 The treatment technique: The Novalis accelerator (BrainLAB AG, Heimstetten, Germany) will be used to deliver SRT. It incorporates stereotactic x-ray capabilities for verifying target position. This consists of two floor mounted x-ray tubes and two opposing amorphous silicon (aSi) flat panel detectors mounted to the ceiling. Each x-ray tube/detector pair is configured to image through the linac isocenter with a coronal field of view of approximately 18cm in both the superior-inferior (S-I) and left-right (L-R) directions at isocenter. For soft tissue targets the system is designed to be used with radio-opaque gold markers implanted near the target. These markers are implanted prior to CT imaging and treatment planning, and should be placed close enough to the target anatomy so that they can be observed within the field of view of the x-ray localization system at the time of treatment. Specific patient breathing characteristics is determined during 4D CT. If

the breathing pattern is adequate, respiratory-gated delivery, which is, turning the beam on only at a specified phase of respiration will be determined. This "freezes" target motion and allows reduction of beam margins, thereby reducing the amount of irradiated normal tissue (in this case, normal liver). The Novalis system is well suited to gated delivery and has been evaluated extensively by Tenn et al (53). The following is a brief procedural summary from that work which will be incorporated into this study: *The patient is set up in the treatment room and IR reflective markers with adhesive bases are attached to their anterior surface so that breathing motion can be monitored. A second set of IR reflective markers is rigidly attached to the treatment couch and used as a reference against which the movement of patient markers is measured. These rigidly mounted reflectors are also used to track couch location during the patient positioning process. The 3D movement of the patient's anterior surface is tracked via the IR markers and the anterior-posterior (A-P) component of this trajectory is used to monitor breathing motion. The system plots breathing motion versus time and a reference level is specified on this breathing trace. This designates the point in the breathing trace at which the verification x-ray images will be triggered. The two images are obtained sequentially at the instant the breathing trace crosses this level during exhale phase. Because the patient is localized based on these images, the gating level is set at the same phase in the breathing cycle at which the planning CT data was obtained. Within each image the user locates the positions of the implanted markers. From these positions the system reconstructs the 3D geometry of the implanted markers and determines the shifts necessary to bring them into alignment with the planning CT. The patient is subsequently positioned according to the calculated shifts. Finally, a gating window (beam-on region) during which the linac beam will be delivered is selected about the reference level. The system can gate the beam in both inhale and exhale phases of the breathing cycle. Subsequent x-ray images verifying the location of the implanted markers locations are obtained at the gating level continuously during treatment. If marker positions remain within tolerance limits the target position may also be assumed to be correctly positioned. If they are outside the limit, the newly obtained images can be used to reposition the patient and maintain treatment accuracy.* When the Artiste and Truebeam linear accelerators are used to deliver radiation therapy, the maximum dimension of the tumor can be up to 10 cm. CT on rail (Artiste) or KV cone beam CT (Truebeam) will be used for image-guided radiation therapy.

5.6.7 Dose computation: The treatment plan used for each patient will be based on an analysis of the volumetric dose, including DVH analyses of the PTV and critical normal structures. Treatment planning should be accomplished with multiple coplanar/noncoplanar conformal beams or arcs to allow for a high degree of dose conformality. The uniformity requirement will be +10% -5% of the total dose at the prescription point within the tumor volume. The IMRT may be used if there is a benefit of decreasing tissue complications.

5.6.8 Equipment and tools. Beam's Eye View techniques will be used to select the beam isocenter and direction to fully encompass the target volume but minimizing the inclusion of the critical organs in order to select the plan that minimizes the dose to normal tissues.

5.6.9 Dose specification. The prescription dose is the isodose which encompasses at least 95% of the planning target volume (PTV). Prescription dose to the PTVs shall be according to the following: The gross tumor and gross lymph node metastasis will receive a total 40 Gy. DVHs must be generated for all critical normal structures (OAR):The dose to the kidney will require careful monitoring and kidney volumes must be defined on simulation fields. The percent of total kidney volume (defined as the sum of the left and right kidney volume) receiving 15 Gy (3 Gy per fraction) should be required to be less than 35% of the total kidney volume. The maximum dose to any point within the spinal cord should not exceed 15 Gy (3 Gy per fraction). At least 700 ml or 35% of normal liver (entire liver minus cumulative GTV) should receive at total dose less than 15 Gy (3 Gy per fraction). The maximum point dose to the stomach or small bowel except duodenum should not exceed 32Gy. An isodose distribution of the treatment at the central axis indicating the position of kidneys, liver and spinal cord is required. Dose recording:The reported doses for each

PTV shall include the prescription dose as well as the maximum point dose, % target volume receiving > 110% and >115% of its prescribed dose and the % target volume receiving < 93% of the prescribed dose, and the mean dose to the PTV. Doses to the organs at risk will also be recorded.Dose homogeneity:No more than 20% of any planning target volume (PTV) will receive >110% of its prescribed dose. No more than 1% of any planning target volume (PTV) will receive <93% of its prescribed dose. No more than 1% or 1 cc of the tissue outside the PTVs will receive >110% of the dose prescribed to the primary PTV.

5.6.10 Fractionation schedule: The total dose of 40 Gy will be delivered in 5 Fractions over 5 consecutive days.

5.6.11 Treatment Verification:The location of the implanted markers will be verified on daily x-rays.

5.6.12 Quality Assurance Documentation: A copy of the daily treatment record will be maintained in the radiation oncology department. Isodose distribution at the central axis will be recorded. Exact track IGRT data will be collected. Breath gating will be used and data will be collected.

5.6.13 Radiation modification for hematologic toxicity: Blood counts will be measured once weekly. Radiation will not be started if the AGC is < 1000/µl and platelets are < 50,000/µl, and the start of radiation will be delayed until the blood counts are above this level.

5.7 Dose Modifications ***(The NCI Common Toxicity Criteria version 4.03, APPENDIX E, will be used, available at http://ctep.cancer.gov/reporting/ctc_v40.html).***

5.7.1 Dose Modifications for Zometa: Because only one dose of Zometa is given, there are no planned dose modifications for Zometa.

5.7.2 Dose Modification for SRT: Because only 5 daily doses of SRT are planned, the radiation will be interrupted only if the patient experiences grade 3 or worse non-hematologic toxicity or grade 4 hematologic toxicity during the period in which SRT is administered. CBC and differential will be measured pre- and post-SRT.

A physician will be immediately available for emergency treatment of the patient in the event of such a life-threatening reaction. The patient will then be continuously monitored until they are stabilized.

5.8 Supportive Care Guidelines

5.8.1 Prophylactic Anti-Emetic Premedication Patients must be pre-medicated for nausea & vomiting with the following antiemetic regimens as outlined below. These recommendations follow the ASCO guidelines for the use of anti-emetic therapy (54): capecitabine5-FU: Dexamethasone 8 mg PO (or equivalent) 30 min prior to 5FU. Radiation to upper abdomen: ondansetron 8 mg or granisetron 2 mg PO daily (or equivalent) < 1 hr prior to radiation, and Prilosec 20 mg PO daily(or equivalent) < 1 hr prior to radiation. Alternative anti-emetic agents:palonsetron 0.25 mg IV pre-therapy (or equivalent), prochlorperazine 5-10 mg PO or 5-10 mg IV q 6-8 hr, promethazine 12.5-25 mg PO/PR/IM/IV q 4-6 hr, lorazepam 1-2.5 mg PO or IV given the night before and just after chemo, ondansetron 8 mg PO or IV.

5.8.2 Diarrhea Patients will be instructed to begin taking loperamide after the first poorly formed or loose stool or first episode of 2 or more bowel movements in one day. Loperamide should be taken in the following manner: 4 mg at the first onset of diarrhea, then, 2 mg every 2 hours around-the-clock until diarrhea-free for at least 12 hours. Patients may take loperamide 4 mg every 4 hours during the night. Loperamide should not be taken prophylactically. Patients must notify the research team as to when they initiated loperamide therapy. If diarrhea persists despite loperamide therapy, then the patient should be evaluated for the need for IV fluid & electrolyte replacement. Alternative anti-diarrhea agents:Somatostatin analog (Octreotide7) 100 - 500 mcg SC/IV tid; maximum daily dose = 1500 mcg/day; alternatively, somatostatin analog may be given at 25-50 mcg/hour as a continuous IV infusion. Atropine/diphenoxylate which is available as either a 0.025/2.5 tab, or 0.025/2.5 per 5 mL liquid. Patients should take 1-2 tabs PO tid or qid or 5-10 mL

PO tid/qid.Atropine/difenoxin (Motofen7) 0.025/1 tab; 2 tabs PO x 1, then 1 tab PO q 2-4 hr (max 8 tabs per day).Paregoric: (an antidiarrheal opiate): 5 - 10 mL ORALLY 1-4 times daily: maximum 40 mL/day. OTC meds: bismuth subsalicylate 262 mg tabs: 2 tabs PO q 1 hr prn; maximum 4200 mg/24 hr

5.8.3 **Stomatitis** Mild symptoms will be treated with topical antiseptic & analgesic agents. Various versions of "magic mouthwash" exist, and represent a combination product for the topical treatment of mild oropharyngeal pain. Such mouthwashes usually consist of viscous lidocaine, diphenhydramine, aluminum and magnesium hydroxide, 70% sorbitol, and orange flavoring. Both viscous lidocaine and diphenhydramine have local anesthetic properties, while the aluminum hydroxide component of Maalox has a beneficial drying property. Patients with severe pain require systemic narcotic analgesics. Topical anti-fungal agents will be added as clinically indicated. More severe symptoms will require all of the above as well as stronger analgesic agents & admission to hospital for IV fluid & electrolyte replacement if dehydrated.

5.8.4 **Treatment of Fever & Neutropenia** Subjects developing a fever of 100.5º C or higher will have a CBC with WBC differential obtained along with a history & physical examination to look for signs of infection. If the ANC is < 500/µL, the patient will be treated with empiric antibiotic therapy as an inpatient & undergo appropriate radiographic & laboratory investigation for sources of infection, & development of a specific treatment plan. Fever & neutropenia occurring during RT will require interruption of RT. If the ANC is between 500-1000/µL, antibiotic therapy will be instituted if there is clinical suspicion of an infection. Daily CBCs with differentials will be obtained if the patient remains febrile. If the ANC is > 1000/µL, & there is no clinical evidence of an infection, then therapy may resume.

5.8.5 **Use of Leukocyte Colony-Stimulating Factors and Erythropoetin:** Colony stimulating factors (CSFs) will be used as clinically indicated according to ASCO guidelines (55). Use of any CSFs must be discontinued at least 24 hours prior to initiation of the next cycle of chemotherapy & must be documented in the patient record. Epoetin will be used as clinically indicated according to ASCO guidelines (56).

5.8.6 **Pancreatic Enzyme Replacement:** Patients with symptoms of pancreatic insufficiency should receive pancreatic enzyme supplement.

5.8.7 **Nutritional Supplementation:** Patients will be evaluated by a nutritionist to review their caloric needs and daily caloric intake. Patients will be encouraged to supplement their nutrition with a high-protein, low fat nutritional drink during the neoadjuvant therapy phase of the trial. Strong consideration should be given for early, elective placement of an enteral feeding tube if the patient has lost 10% or more of their body weight.

5.9 **Duration of Study:** We estimate that there will be about 40 patients enrolled into of this protocol treated over a 5 year period

5.10 **Duration of Follow up:** The patient will be seen every 3 months for the 1st year and every 4 months for 2nd year, then every six months thereafter. The patients will be assessed for long term toxicity from combination of Zometa and radiation. Restaging radiographic scans, CBC and chemistry panel will be performed at each of these visits as part of standard of care.

5.11 **Post-trial Assessments:** Patients who go off study treatment at any time during the trial will be followed for 30 days after the last day of treatment or until other disease-related treatment begins. For all patients, drug-related SAEs and AEs will be followed until baseline or ≤ grade 1 levels. Patients may refuse to participate in the post-trial assessments.

5.12 **Criteria for Removal from Study**

5.12.1 Progression of Disease

5.12.2 If at any time the constraints of this protocol are detrimental to the patient's wellbeing, or if the patient is unable to comply with the requirements of the protocol, the patient will be removed from protocol therapy. In this event, the reason(s) for withdrawal will be documented.

5.12.3 If the patient experiences an adverse reaction that, in the opinion of the investigator, necessitates the removal of the patient from the study, including any unresolved serious adverse event.

5.12.4 Any patient who suffers a serious systemic allergic response or severe degree of intolerance to the study medication will be withdrawn from further study treatment but will be followed up for a period of three years following treatment.

5.12.5 Development of intercurrent medical problems that would make continued protocol therapy detrimental to the patient's safety.

5.12.6 There is concurrent illness or other reasons that would, in the opinion of the investigator, affect assessment of clinical status or conduct of the study to a significant degree.

5.12.7 The patient completes study treatment as per study schedule.

5.12.8 The patient chooses to discontinue treatment or follow-up.

In this event, the reason(s) for withdrawal will be documented. The reason(s) for withdrawing the patient from the treatment portion of the study will be documented in the case report form. If available, the following information will be recorded in the case report form: date of disease relapse, date of death, cause of death, and autopsy report.

6.0 MEASUREMENT OF EFFECT

6.1 Tumor Response: Measurable Disease Response: CTEP's. Criteria for Radiographic Response Evaluation (RECIST) guidelines will be followed: A quick reference to the RECIST guidelines can be downloaded at the following URL: http://ctep.cancer.gov/guidelines/recist.html Patients enrolled in this study must have a measurable pancreatic cancer which is defined as lesions that can be accurately measured in at least one dimension: [longest diameter to be recorded] on the CT scan or MRI scan. The same method of assessment & the same technique should be used to characterize each identified & reported lesion at baseline & during follow-up.

Parameters to Measure Response Outcome:	
1	Clinical examination
2	Chest radiograph
3	CT & MRI scans
4	Tumor markers
5	Cytology & histology

6.2 Response criteria:

For CT/MRI:

Taking into account the measurement of the longest diameter only for those lesions with size response, response criteria are defined as:

Complete Response (CR): the disappearance of a lesion.

Partial Response (PR): at least a 30% decrease in the longest diameter of a lesion, taking as reference the longest diameter recorded since the treatment started.

Stable Disease (SD): neither sufficient shrinkage to qualify for partial response nor sufficient increase to qualify for progressive disease, taking as reference the longest diameter since the treatment started.

Progressive Disease (PD): at least a 25% increase in the longest diameter of a lesion, taking as reference the longest diameter recorded since the treatment started.

For PET: PET Response Criteria in Solid Tumors (PERCIST) will be followed:
CMR:
Complete resolution of 18F-FDG uptake within the measurable target lesion so that it is less than mean liver activity and at the level of surrounding background blood pool activity.Disappearance of all other lesions to background blood pool levels.
No new suspicious 18F-FDG avid lesions.
PMR:
Reduction of a minimum of 30% in target measurable tumor 18F-FDG SUL peak, with absolute drop in SUL of at least 0.8 SUL units.
No increase >30% of SUL or size in all other lesions
No new lesions
SMD:
Not CMR, PMR, or Progressive metabolic disease (PMD)
No new lesions
PMD:
>30% increase in 18F-FDG SUL peak, with >0.8 SUL units increase in tumor SUV peak from the baseline scan in pattern typical of tumor and not of infection/treatment effect.
OR
Visible increase in the extent of 18F-FDG tumor uptake.
OR
New 18F-FDG avid lesions which are typical of cancer and not related to treatment effect or infection. PET improves evaluation of patients with normal or equivocal CT findings. PET response will override the CT/MRI response. For example, if the patient has a PR or SD or PD on CT/MRI but a CMR on PET, then it will be considered as CR (The scar tissue can be bigger than the tumor on a CT scan but will be negative on PET). If the patient has a SD on a CT scan but a PMD on PET, then it will be considered as PD.

6.3 **Time to Treatment Failure:** The time to treatment failure will be defined as from the first date of therapy until the date the patient is removed from study for any reason.

6.4 **Survival:** Survival will be defined as from the first date of therapy until the date the patient dies.

6.5 **Toxicity criteria:** The NCI Common Toxicity Criteria Adverse Events version 4.03 will be used to grade toxicity; it is available at the following internet site: http://ctep.cancer.gov/forms/CTCAEv4.pdf.

6.6 **Monitoring of peri-and post-marker implantation morbidity:** The marker position and geometry will be documented on simulation CT images. Information regarding the development of infectious complications, bleeding, and any implantation associated adverse events will be recorded.

7.0 STUDY PARAMETERS

	Screening Staging Marker **BASELINE**	Zometa	RT	Break				OR			
Week		1	1	2	3	4	5	6	7	8	9
Day	-3 to -30	1	1	8	15	21	28	15	1	8	15
Eligibility	X										
**Serum Pregnancy Test** (If Female)	X										
**Pain assessment** prior to the start of RT, 3-4 weeks post radiation, and 2-4 weeks post-surgery	X		X				X				
**Zometa**		X									
**Stereotactic Radiotherapy**			X								
**Surgery**								X			
**History/physical examination**	X	Every month									
**Weight**	X	Every month									
**Performance status** (Karnofsky)	X	Every month									
**CBC, diff, Plt**	X	Every month									
**Comprehensive metabolic panel**	X	Every month									
**CA19-9**	X	Every month									
**PET/CT**	X	Prior to Surgery									
**MRI abdomen**	X	Prior to Surgery									
**CT Chest/abdomen/pelvis with oral/IV contrast**	X	Prior to Surgery									

Zometa 4 mg IV is given day 1: 2-4 hours prior to RT
RT = hypofractionated radiation therapy daily Monday-Friday x 1 week to start at week 1
OR= Surgery to be performed sometime during week 6-9

8.0 DRUG FORMULATION AND PROCUREMENT

8.1 Zometa

8.1.1 Chemistry Chemical Name:(1-Hydroxy-2-imidazol-1-yl-phosphonoethyl) phosphonic acid monohydrate. Chemical formula:C5H10N2O7P2. H2O, molecular mass:272.09 g/mol, Physical form: A white crystalline powder, Solubility:highly soluble in 0.1N sodium hydroxide solution, sparingly soluble in water and 0.1N hydrochloric acid, and practically insoluble in organic solvents.

8.1.2 General description: Preclinical data indicate that low micromolar concentrations of zometa are cytostatic and pro-apoptotic in vitro to a range of human cancer cell lines (breast, prostate, lung, bladder, myeloma), and that this anti-tumor efficacy can be synergistically enhanced by combination with other anti-cancer drugs . Zometa is also anti-proliferative for human fetal osteoblasts and promotes their differentiation, a property potentially relevant for the treatment of bone metastases in prostate cancer. Zometa inhibits the proliferation of human endothelial cells in vitro and is anti-angiogenic in vivo. Zometa at picomolar concentrations inhibits tumor cell invasion through extracellular matrix.

8.1.3 Pharmacokinetics

8.1.3.1 Distribution: Single or multiple (q 28 days) 5-minute or 15-minute infusions of 2, 4, 8 or 16 mg Zometa® were given to 64 patients with cancer and bone metastases. The post-infusion decline of zometa concentrations in plasma was consistent with a triphasic process showing a rapid decrease from peak concentrations at end-of-infusion to <1% of Cmax after 24 hours post infusion with population half-lives of t ½α 0.24 hours and t ½β 1.87 hours for the early disposition phases of the drug, and followed by a prolonged period of very low concentrations in plasma between days 2 and 28 post infusion, with a terminal elimination half-life t½γ of 146 hours. The area under the plasma concentration versus time curve (AUC0-24h) of zometa was linearly related to dose. The accumulation of zometa measured over three cycles was low, with mean AUC0-24h ratios for cycles 2 and 3 versus 1 of 1.13 ± 0.30 and 1.16 ± 0.36, respectively. In vitro and ex vivo studies showed low affinity of zometa for the cellular components of human blood. Binding to human plasma proteins was approximately 56% and independent of the concentration of zometa.

8.1.3.2 Metabolism: Zometa does not inhibit human P450 enzymes in vitro. Zometa does not undergo biotransformation in vivo. In animal studies, <3% of the administered intravenous dose was found in the feces, with the balance either recovered in the urine or taken up by bone, indicating that the drug is eliminated intact via the kidney. Following an intravenous dose of 20 nCi 14C-zoledronic acid in a patient with cancer and bone metastases, the radioactivity excreted in the urine consisted solely of intact drug.

8.1.3.3 Excretion: In 64 patients with cancer and bone metastases on average (± s.d.) 39 ± 16% of the administered zometa dose was recovered in the urine within 24 hours, with only trace amounts of drug found in urine post day 2. The cumulative percent of drug excreted in the urine over 0-24 hours was independent of dose. The balance of drug not recovered in urine over 0-24 hours, representing drug presumably bound to bone, is slowly released back into the systemic circulation, giving rise to the observed prolonged low plasma concentrations days 2 to 28 post dose. The 0 – 24 hour renal clearance of zometa was on average (± s.d.) 3.7 ± 2.0 L/h. Zometa clearance was independent of dose and demographic variables. Effects of body weight, gender, and race on clearance were within the bounds of the inter-patient variability of clearance, which was 36%. In a study in patients with cancer and bone metastases, increasing the infusion time of a 4 mg dose of zometa from 5 minutes (n=5) to 15 minutes (n=7) resulted in a 34% decrease in the zometa concentration at the end of the infusion ([mean + SD] 403 + 118 ng/mL vs 264 + 86 ng/mL) and a 10% increase in the total AUC (378 + 116 ng x h/mL vs 420 + 218 ng x h/mL). The difference between the AUC means was not statistically significant.

8.1.4 Clinical Trial

8.1.4.1 Hypercalcemia of Malignancy: Two identical multicenter, randomized, double-blind, double-dummy studies of Zometa 4 mg given as a 5-minute intravenous infusion or pamidronate 90 mg given as a 2-hour intravenous infusion were conducted in 185 patients with hypercalcemia of malignancy (HCM). NOTE: Administration of Zometa 4 mg given as a 5-minute intravenous infusion has been shown to result in an increased risk of renal toxicity, as measured by increases in serum creatinine, which can progress to renal failure. The incidence of renal toxicity and renal failure has been shown to be reduced when Zometa 4 mg is given as a 15-minute intravenous infusion. Zometa should be administered by intravenous infusion over no less than 15 minutes. (See WARNINGS and DOSAGE AND ADMINISTRATION.) The treatment groups in the clinical studies were generally well balanced with regards to age, sex, race, and tumor types. The mean age of the study population was 59 years; 81% were Caucasian, 15% were Black, and 4% were of other races. Sixty percent of the patients were male. The most common tumor types were lung, breast, head and neck, and renal.In these studies, HCM was defined as a corrected serum calcium (CSC) concentration of ≥12.0 mg/dL (3.00 mmol/L). The primary efficacy variable was the proportion of patients having a complete response, defined as the lowering of the CSC to ≤

10.8 mg/dL (2.70 mmol/L) within 10 days after drug infusion. To assess the effects of Zometa versus those of pamidronate, the two multicenter HCM studies were combined in a pre-planned analysis. The results of the primary analysis revealed that the proportion of patients that had normalization of corrected serum calcium by Day 10 were 88% and 70% for Zometa 4 mg and pamidronate 90 mg, respectively (p=0.002). (see Figure 1) In these studies, no additional benefit was seen for Zometa 8 mg over Zometa 4 mg; however, the risk of renal toxicity of Zometa 8 mg was significantly greater than that seen with Zometa 4 mg.

8.1.5 Precautions: Patients being treated with Zometa should not be treated with Reclast. Adequately rehydrate patients with hypercalcemia of malignancy prior to administration of Zometa and monitor electrolytes during treatment. Monitor serum creatinine before each dose. Osteonecrosis of the jaw has been reported. Preventive dental exams should be performed before starting Zometa. Avoid invasive dental procedures. Zometa can cause fetal harm. Women of childbearing potential should be advised of the potential hazard to the fetus and to avoid becoming pregnant. Hypocalcemia: Correct before initiating Zometa. Adequately supplement patients with calcium and vitamin D. Monitor serum calcium closely with concomitant administration of other drugs known to cause hypocalcemia to avoid severe or life-threatening hypocalcemia. Contraindications: Hypersensitivity to any component of Zometa or severe renal impairment**.** Side effects: The most common adverse events (greater than 25%) were nausea, fatigue, anemia, bone pain, constipation, fever, vomiting, and dyspnea. Atypical subtrochanteric and diaphyseal femoral fractures have been reported in patients receiving bisphosphonate therapy. These fractures may occur after minimal or no trauma. Evaluate patients with thigh or groin pain to rule out a femoral fracture.

8.1.6 Route of Administration: Zometa® is available in vials as a sterile powder for reconstitution for intravenous infusion. Each vial contains 4.264 mg of zometa, corresponding to 4 mg zometa on an anhydrous basis. Dosage forms: 4 mg/100 mL single-use ready-to-use bottle or 4 mg/5 mL single-use vial of concentrate. Dose and Usage: 4 mg as a single-use intravenous infusion over no less than 15 minutes. Reduce the dose for patients with renal impairment. Coadminister oral calcium supplements of 500 mg and a multiple vitamin containing 400 international units of vitamin D daily. Administer through a separate vented infusion line and do not allow to come in contact with any calcium or divalent cation-containing solutions

8.2 Fluorouracil (5-FU)

8.2.1 Chemistry

Chemical Name:	5-fluoropyrimidine- 2,4(1H,3H)-dione.
Chemical formula:	$C_4H_3FN_2O_2$
molecular weight:	130.1
Physical form:	A white to almost white, practically odourless, crystalline powder
Solubility:	Sparingly soluble in water; slightly soluble in ethanol

8.2.2 Mechanism of Action: 5-FU is an analog of uracil, & can be metabolized intracellularly to both ribonucleotides & deoxyribonucleotides. Fluorouridine triphosphate can be incorporated into RNA by RNA polymerase, & consequently interferes with RNA processing & function. Fluorodeoxyuridine monophosphate (FdUMP) is a potent inhibitor of thymidylate synthase, the enzyme in the de novo pathway of pyrimidine biosynthesis that coverts dUMP to dTMP. Depletion of dTTP interferes with DNA synthesis & repair. FdUTP can also be incorporated into DNA.

8.2.3 Clinical Formulation: 5-FU is available in 10 ml ampules as a colorless aqueous solution containing 500 mg/10 ml. The pH of the solution is adjusted to approximately 9.2 with sodium hydroxide. This undiluted preparation is suitable for direct intravenous injection.

Storage: Unused vials may be stored at room temperature & should be protected from light. If a precipitate forms due to exposure to low temperatures, resolubilize by heating to 140 1F & shaking vigorously. Allow the solution to cool to body temperature before administering.
Stability: The vials bear an expiration date. 5-FU solutions may discolor slightly during storage, but the potency & safety are not adversely affected. 5-FU prepared for protracted continuous infusion is stable for 14 - 28 days (Handbook on Injectable drugs: Lawrence A. Trissel, 9th Edition. American Society of Health-System Pharmacists= Product Development Office. 1996).

8.2.4 Clinical Pharmacology: 5-FU is cleared predominantly by enzymatic means: catabolism of 5-FU to dihydrofluorouracil by dihydropyrimidine dehydrogenase is the rate-limiting step in 5-FU clearance. The half-life of 5-FU in plasma averages 8-12 minutes. Less than 10% of the administered dose is excreted as parent drug in the urine.

8.2.5 Clinical toxicity

GI: nausea, vomiting, diarrhea, anorexia, enteritis, stomatitis, esophagopharyngitis, GI bleeding
Cutaneous: alopecia, dermatitis, pigmentation abnormalities, palmar plantar erythrodysesthesia, erythema, photosensitivity, ulceration
Systemic: lethargy, malaise
Ocular toxicity: conjunctivitis, lacrimation, lacrimal duct stenosis, photophobia
Myelosuppression: leukopenia, thrombocytopenia, anemia
Cardiac: chest pain and rhythm abnormalities
CNS toxicity: acute cerebellar syndrome, confusion, headache

8.2.6 Drug Interactions: Calcium leucovorin, warfarin. Possible: zidovudine, allopurinol. Several investigational drugs are inhibitors of dihydropyrimidine dehydrogenase; these include the antiviral drugs brivudine and soruvidine.

8.2.7 Route of Administration: 5-FU will be given as a 24 hr continuous IV infusion starting on day one of RT: weekly for 4 weeks using an ambulatory infusion pump.

8.3 Capecitabine

8.3.1 Other names: Xeloda®
Classification: Antimetabolite, cytotoxic.

8.3.2 Mechanism of Action: Capecitabine is a fluoropyrimidine carbamate with antineoplastic activity. It is an orally administered systemic prodrug of 5'-deoxy-5-fluorouridine (5'-DFUR) which is converted to 5-fluorouracil.

8.3.3 Storage and stability: Capecitabine should be stored at room temperature, excursions permitted to 15° to 30°C (59° to 86°F), with container tightly closed.

8.3.4 Route of administration: Tablets should be swallowed with water 30 minutes after the end of a meal (breakfast and dinner). Take daily starting the evening before day 1 of RT until the end of 4 weeks.

8.3.5 Availability: Capecitabine is supplied as a biconvex, oblong film-coat tablets for oral administration. Each light-peach colored tablet contains 150 mg capecitabine, and each peach colored tablet contains 500 mg capecitabine. Capecitabine is commercially available.

8.3.6 Pharmacology: Capecitabine is a prodrug of 5-fluorouracil, activated by a cascade of three enzymes. After oral administration it passes unchanged from the GI tract and is metabolized in the liver by 60 kDa carboxylesterase (previously known as acylamidase isozyme A), to 5'-DFCR. This is then converted to 5'-DFUR by cytidine deaminase located in the liver and also in tumor tissues. Further metabolism of 5'-DFUR then occurs at the site of the tumor under the action of PyNPase, to 5-FU. The exposure of normal body tissues to systemic 5-FU is, therefore, minimized. The peak plasma concentrations for the drug and its two main metabolites occurs shortly (0.5 - 1.5 hours) after administration. The concentrations decline exponentially with a half-life of 0.5 - 1 hour

8.3.7 Adverse Events

Blood: neutropenia, coagulation disorder, idiopathic thrombocytopenic purpura, pancytopenia.
Cardiac: angina pectoris, cardiomyopathy. For ≥ Grade 2 cardiac toxicity that is attributable to 5-FU or capecitabine, patients will be permanently discontinued from therapy.
Gastrointestinal: Diarrhea, nausea, vomiting, stomatitis, intestinal obstruction, rectal bleeding, GI hemorrhage, esophagitis, gastritis, colitis, duodenitis, hematemesis, necrotizing enterocolitis.
Dermatologic: Hand-and-Foot Syndrome (painful erythema and swelling of the hands and/or feet), increased sweating, photosensitivity, radiation recall syndrome.
Infections: fever, oral candidiasis, upper respiratory tract infection, urinary tract infection, bronchitis, pneumonia, sepsis, bronchopneumonia, gastroenteritis, gastrointestinal candidiasis, laryngitis, esophageal candidiasis.
Immune System: drug hypersensitivity
Hepatobiliary: hepatic fibrosis, cholestatic hepatitis, hepatitis.
Metabolism: cachexia, hypertriglyceridemia.

8.3.8 Drug interactions

Sorivudine and Brivudine: A metabolite of these investigational antiviral agents, 5-bromovinyluracil, is a potent inhibitor of dihydropyrimidine dehydrogenase, the enzyme that catabolyzes 5-FU. Patients should not receive concurrent therapy with either of these antiviral agents while receiving capecitabine.
Musculoskeletal: bone pain, joint stiffness.
Neurological: ataxia, encephalopathy, depressed level of consciousness, loss of consciousness.
Phenytoin: Increased phenytoin plasma concentrations have been reported during concomitant use of capecitabine with phenytoin, suggesting a potential interaction. Patients taking phenytoin concomitantly with capecitabine should be regularly monitored for increased phenytoin plasma concentrations and associated clinical symptoms
Psychiatric: confusion.
Respiratory: dyspnea, epistaxis, bronchospasm, respiratory distress.
Renal and Urinary: nocturia
Vascular: hypotension, hypertension, venous phlebitis and thrombophlebitis, deep venous thrombosis, lymphoedema, pulmonary embolism, cerebrovascular accident.

9.0 TOXICITY REPORTING GUIDELINES

Reporting until 30 days after surgery. This protocol will comply with monitoring and adverse event reporting requirements of the UNMC/Fred & Pamela Buffett Cancer Center Data Monitoring plan. The protocol will adhere to the institutional and FDA guidelines for the toxicity reporting.All patients will be closely followed for toxicity from the time of informed consent until 30 days after last administration of study medication. Adverse event and serious adverse events will be followed until baseline or ≤ grade 1 levels. Toxicity will be assessed using the NCI CTCAE version 4.03 (Appendix E). All adverse events will be followed to a satisfactory conclusion. Serious adverse events should be followed until resolution, death, or until no further improvement is reasonably expected. Deaths occurring within 30 days of study treatment regardless of relationship will be reported to the UNMC IRB and Fred & Pamela Buffett Cancer Center DSMC.In addition to complying with all applicable regulatory reporting laws and regulations, all serious adverse events and toxicities will be reported to the University of Nebraska Medical Center, Institutional Review Board (IRB) and Fred & Pamela Buffett Cancer Center Cancer Center Data and Safety Monitoring Committee (DSMC).

9.1 Definitions

9.1.1 Adverse Event

An adverse event (AE) is defined as any untoward medical occurrence in a subject or clinical

investigation subject administered a pharmaceutical product and which does not necessarily have a causal relationship with this treatment. An adverse event can therefore be any unfavorable and unintended sign (including an abnormal laboratory finding), symptom, or disease temporally associated with the use of a medicinal (investigational) product, whether or not the event is considered causally related to the use of the product.

An elective surgery or procedure that is scheduled to occur during a study will not be considered an adverse event if the surgery or procedure is being performed for a pre-existing condition and the surgery or procedure has been planned before study entry. However, if the pre-existing condition deteriorates unexpectedly during the study (*e.g.,* the surgery is performed earlier than planned), then the deterioration of the condition for which the elective surgery or procedure is being done will be considered an adverse event.

An adverse event can result from use of the drug as stipulated in the protocol or labeling, as well as from accidental or intentional overdose, drug abuse, or drug withdrawal. Any worsening of a pre-existing condition or illness is considered an adverse event. Laboratory abnormalities and changes in vital signs are considered to be adverse events if they result in discontinuation from the study, necessitate therapeutic medical intervention, meet protocol specific criteria (see Section 5.0, Treatment Plan) and/or if the investigator considers them to be adverse events. In general, if a laboratory abnormality or change in vital sign is associated with a specific diagnosis that is being reported concurrently as an adverse event (e.g. elevated creatinine with renal failure or sinus tachycardia in febrile neutropenia) the findings that support the diagnosis do not need to be reported as separate adverse events unless the investigator feels it is appropriate.

9.1.2 Treatment-emergent Adverse Event

Treatment-emergent adverse event is defined as any adverse event with onset or worsening from the time that the first dose of study drug is administered until 30 days after the final dose of study drug is administered.

9.1.3 Unexpected Adverse Event

An unexpected adverse event is any adverse drug event that is not listed in the current labeling/Investigator's Brochure. This includes events that may be symptomatically and pathophysiologically related to an event listed in the labeling, but differ from the labeled event because of greater severity or specificity. For example, under this definition, hepatic necrosis would be unexpected (by virtue of greater severity) if the labeling only referred to elevated hepatic enzymes or hepatitis. Similarly, cerebral thromboembolism and cerebral vasculitis would be unexpected (by virtue of greater specificity) if the labeling only listed cerebral vascular accidents. "Unexpected," as used in this definition, refers to an adverse drug experience that has not been previously observed (i.e., included in the labeling) rather than from the perspective of such experience not being anticipated from the pharmacological properties of the pharmaceutical product.

9.1.4 Serious Adverse Event

A serious adverse event is one that at any dose (including overdose) and regardless of causality that:

- Results in death
- Is life-threatening[1]
- Requires inpatient hospitalization or prolongation of existing hospitalization
- Results in persistent or significant disability or incapacity[2]
- Is a congenital anomaly or birth defect

- Is an important medical event[3]
- Pregnancy

[1]"Life-threatening" means that the subject was at immediate risk of death at the time of the serious adverse event; it does not refer to a serious adverse event that hypothetically might have caused death if it were more severe.

[2]"Persistent or significant disability or incapacity" means that there is a substantial disruption of a person's ability to carry out normal life functions.

[3]Medical and scientific judgment should be exercised in deciding whether expedited reporting is appropriate in situations where none of the outcomes listed above occurred. Important medical events that may not be immediately life-threatening or result in death or hospitalization but may jeopardize the patient or may require intervention to prevent one of the other outcomes listed in the definition above should also usually be considered serious. Examples of such events include allergic bronchospasm requiring intensive treatment in an emergency room or at home, blood dyscrasias or convulsions that do not result in inpatient hospitalization, or the development of drug dependency or drug abuse. A new diagnosis of cancer during the course of a treatment should be considered as medically important.

9.2 Adverse Event Reporting and Definitions Per University of Nebraska Medical Center, IRB and Fred & Pamela Buffett CancerCenter Data and Safety Monitoring Committee (DSMC) and Allos Therapeutics Drug Safety and Surveillance

This protocol will adhere to all institutional guidelines for adverse event reporting. Adverse events will be evaluated using the NCI Common Terminology Criteria for Adverse Events (CTC-AE) version 4.02. (Appendix E)

9.2.1 IRB REPORTING

All internal serious adverse events (AE) must be reported to the IRB promptly through the electronic RSS system and in no case later than two (2) business days following PI notification that the event occurred *if* the principal investigator determines that conditions A, B, and C are met:

a. The AE is unexpected, *AND*
b. The AE is related to, or possibly related to, the drug, biologic, device, or other research intervention, *AND*
c. The AE is more than minor in nature which is defined as requiring treatment from a health professional.

All *unexpected*, internal, fatal AEs must be reported promptly to the IRB, no later than ***24 hours*** through the electronic RSS system following PI notification that the event occurred. If documentation is still pending, the IRB office must be notified by a telephone call or e-mail.

The RSS system is accessed through a link on the UNMC IRB website (http://unmc.edu/irb).

9.2.2 FRED & PAMELA BUFFETT CANCER CENTER DATA AND SAFETY MONITORING COMMITTEE (DSMC) REPORTING

All serious adverse events (expected or unexpected, regardless of attribution) and toxicities ≥ grade 3 will be reported to the University of Nebraska Medical Center, Fred & Pamela Buffett Cancer Center Data and Safety Monitoring Committee (DSMC) in accordance with DSMC guidelines. The investigator will assign a causal relationship for all reportable AE's, using the terminology of probably related (AE has strong temporal relationship to study drug or recurs on re-challenge, another etiology is unlikely or significantly less likely), possibly related (AE has strong temporal relationship to study drug, alternative etiology is equally or less likely), probably not related (AE has little or no temporal relationship to study drug and/or a more likely etiology exists), or not related (AE related to underlying or concurrent illness).

AEs will be collected from the time the subject signs the consent form and ending 30 days following the final chemotherapy. All AEs will be followed until resolution or a cause is identified. Prescription medication taken to relieve symptoms of the AE will be recorded in addition to the

outcome. AEs judged by the investigator as not related or probably not related to the treatment will not be followed beyond the 30 days after the final chemotherapy. Transplant related Adverse Experiences (AE's) or Serious Adverse Experiences (SAE's) will NOT be collected.

Severity of AE. The severity of events reported on the AE case report form will be determined by the principal investigator according the NCI Common Toxicity Criteria (CTC version 4.03).

The likelihood of relationship of the AE to the study drugs will be determined by the investigator based on the following definitions:

Not related: The subject was not exposed to the study treatment or another cause is obvious.

Probably not related: The AE is most likely explained by another cause, and the time of occurrence of the AE is not reasonably related to the study treatment.

Possibly related: Study treatment administration and AE occurrence reasonably related in time, and the AE is explained equally well by causes other than study treatment, or treatment administration and AE occurrence are not reasonably related in time, but the AE is not obviously a result of other causes.

Probably related: Study treatment administration and AE occurrence are reasonably related in time, and the AE is more likely explained by study treatment than by other mechanisms.

Definitely related: There occurrence and timing of the AE are clearly attributable to the study treatment.

Copies of the AE report will be submitted to the IRB (when required), the Fred & Pamela Buffett Cancer Center's Data Safety and Monitoring Committee and the Fred & Pamela Buffett Cancer Center Clinical Trials Office.

It is the responsibility of the sponsor-investigator to submit to the FDA IND Safety Reports in accordance with 21 CFR 312.32. In addition the sponsor-investigator must notify the Ethics Review Committee/Institutional Review Board (EC/IRB) of a serious adverse event in writing in accordance with international and local laws and regulations. SAEs not meeting expedited criteria will be made available to FDA by the sponsor-investigator via the annual report. The Investigator will utilize the FDA MedWatch Form (Appendix F) for the reporting of adverse events and follow up information to those events. The form can be found at the following URL: http://www.fda.gov/medwatch

Additionally, serious adverse events will be reported to the IRB, SRC and the Data Safety Monitoring Committee by the Investigator.

9.3 Monitoring

The UNMC Fred & Pamela Buffett Cancer Center Scientific Review Committee will review this protocol on at least an annual basis. In its initial review, the DSMC will make a recommendation for the frequency of DSMC monitoring based on an assessment of risk associated with study-associated therapy, per the DSMC policy.All adverse events and toxicity reporting will be reported

to the UNMC Fred & Pamela Buffett Cancer Center Data and Safety Monitoring Committee (DSMC). This study will undergo audit on at least a quarterly basis by the UNMC Fred & Pamela Buffett Cancer Center Audit Committee.

10.0 STATISTICAL CONSIDERATIONS

Study Design: This is an open-label, randomized Phase II, Simon two-stage study with an safety run-in in subjects with locally advanced pancreatic adenocarcinoma. Since this is an open-label trial, descriptive statistics will be employed to analyze the data. Summary statistics for continuous variables will include the mean, standard deviation, median, and range (minimum, maximum). Categorical variables will be presented as frequency counts and percentages and time-to-event variables will be summarized by Kaplan-Meier plots, medians and ranges. All statistical tests will be one-sided with a type I error rate of 10%. All confidence intervals will be constructed at the 90% confidence level. All statistical analyses will be performed using SAS Version 9.4 or later. Failure-free survival will be defined as the date of administration study drug to the date of first appearance of tumor lesions by imaging, or death. Overall survival will be measured from the date of first of study drug to the date of death. Patients who are lost to follow-up will be censored at the date they were last known to be alive.

10.1 Primary End Point

10.1.1 To evaluate local control at 4, 8 and 12 months of follow-up.

10.2 Secondary End Points

10.2.1 To examine the toxicity of the current used dose of Zometa when given concurrently with hypofractionated radiation therapy. Toxicity will be graded by NCI Common Toxicity Criteria (CTCAE Version 4.03). (The currrent used dose of Zometa will be reduced if there are more than one out of first six patients with unexpected grade 3 hematologic or non-hematologic toxicities not medically controlled that is possibly, probably or definitely related to Zometa)

10.2. To evaluate local failure-free survival and overall survival.

10.2. To evaluate the surgical complete resection (negative margin) rate.

10.2. To evaluate tumor response rate on pathology specimen (see 6.2.2 for definition of response).

10.2. To evaluate tumor response rate on PET/CT/MRI (see 6.2.2 for definition of response)

10.2. To evaluate tumor and organ motion with 4D CT and respiratory gating system.

10.2. To evaluate the effect of tumor/organ motion on the dosimetry, local control and survival.

10.3 Correlative endpoints:

10.3.1 To quantify the amplitude of the expression of genes that are involved in CBS (ACAT2, DHCR7, ELFN2, FASN, SC4MOL, and SQLE) in pancreatic tumor tissue prior to radiation and following Zometa and radiation therapy if tissue is available;

10.3.2 To measure Zometa pharmacokinetics at steady-state;

ARM B: After patients have received Zometa , serial blood samples will be obtained at the following time points during steady-state:

*0 h (to measure the trough concentration), 1 h, and before radiation treatments on days,2,3,4,5.

ARM A: (non-zometa arm) subjects will have a pre and post radiation therapy blood sample drawn on day one. Blood will be collected into 10-ml green-top Vacutainer tubes containing sodium heparin. The collected heparinized blood will be placed on ice immediately and transported to the laboratory of Dr. Batra (ESH 9015). The samples will be centrifuged for 10 min at 800 x g at 4º C as soon as possible. The plasma will then be removed from the tube, placed in a labeled cryovial, immediately frozen and stored at ≤ -20º C until analysis. Pharmacokinetic log sheets will be used to record the time the dose of Zometa is given, the planned time of the samples, the actual time the samples were taken,. The blood collection tubes

will have pre-prepared labels that contain a unique patient identification number, date and time. The actual time is written on the tube when the sample is drawn.

10.3.3 To evaluate the value of 4DCT and respiratory gating in pancreatic cancer SRT

10.4 Sample Size

We estimate that there will be about 20 patients enrolled into each arm over a 5 year period; the first six patients in the arm that patients receiving Zometa will also be evaluated for the safety of Zometa given concurrently with radiation therapy.

With conventional fractionated radiation therapy, about 60% of these patients would be expected to develop local recurrence by four months of follow-up. With the radiation therapy and Zometa outlined in this protocol, we would hope to reduce this rate to 30%. The study design will follow a two-stage design using a response endpoint to determine if the regimen is acceptable. The following monitoring rule will be applied: Optimal two-stage design to test the null hypothesis that P<=0.400 versus the alternative that P>=0.700, alpha=0.10, 90% power. After testing the regimen on 11 patients in the first stage, the trial will be terminated if 5 or fewer patients have local control. If the trial goes on to the second stage, a total of 20 patients will be studied. If the total number of patients with local control is less than or equal to 10, the regimen is rejected.

10.5 Interim analyses

The first interim analysis will occur when 6 subjects have completed treatment and 1 month follow up in the arm receiving Zometa. Further enrollment will be halted if there is more than one patient with unexpected grade ≥ 4 hematologic or non-hematologic toxicities not medically controlled that is possibly, probably or definitely related to Zometa.

The second interim analysis will occur when 11 evaluable subjects have completed treatment and 4 months of follow up in each arm. Further enrollment into the cohort will be halted if there are < 5 patients have local control observed among the 11 subjects within 4 months of follow up from the end of the radiation therapy in both Zometa and control arms. The enrollment will continue if one arm has ≥ 5 patients have local control observed among the 11 subjects within 4 months of follow up from the end of the radiation therapy.

The final analysis will occur when all patients have completed their study participation.

10.6 Stopping Rules

Monitoring for toxicity: The study would be suspended if 2 or more toxic deaths were observed in the first 14 patients (14%). This monitoring rule would indicate that the treatment is too toxic about 6% of the time if the true toxic death rate was 4%, and would be considered too toxic 66% of the time if the true toxic death rate was 15%. Pending a review of the toxicity experience, the protocol would either be terminated, or amended to specify modifications in the therapy to reduce the risk of toxicity.

10.7 Analysis populations

All patients who received study medication will be considered evaluable for the safety analysis regardless of the duration of treatment.

10.8 Efficacy analysis:

Local progressive disease (LPD) is defined as at least a 50% increase in the longest diameter of a lesion, taking as reference the longest diameter recorded since the treatment started. The number

and proportion of patients experiencing LPD will be reported. An exact one-sided 90% confidence interval will be constructed round the local progressive disease rate.

Secondary endpoints of surgical complete resection rate, pathological response rate, tumor response rate on PET/CT/MRI will be analyzed as described above for disease control. Failure-free survival and overall survival will be analyzed using Kaplan-Meier plots, medians and ranges.

10.9 Safety analysis

Safety variables to be analyzed are adverse events. Adverse events will be tallied for overall frequency (number and percentage of subjects), worst reported severity, and relationship to study drugs. Serious adverse events will be summarized similarly. Listings of deaths, SAEs and AEs leading to early termination of study treatment or premature withdrawal from study will also be provided.

11.0 RECORDS TO BE KEPT

Information regarding the actual treatments, adverse effects, radiographic and laboratory information, and pathology are to be recorded on appropriate forms. See attached Data forms. Serious adverse events, when noted, will be recorded on site via the standard serious adverse effects form.

11.1 Quality assurance: Complete records must be maintained in a research chart on each patient treated on the protocol. These records should include primary documentation (e.g., lab. report slips, X-ray reports, scan reports, pathology reports, physician notes, etc.) which confirm that:

- The patient met the eligibility criteria.
- Signed informed consent was obtained prior to treatment.
- Treatment was given according to protocol (dated notes about doses given & reasons for any dose modifications).
- Toxicity was assessed according to protocol (laboratory report slips, etc.).
- Response was assessed according to protocol (x-ray, scan, lab reports, dated notes on measurements & clinical assessment, as appropriate).

11.2 Forte Research (Oncore) EDC

Data will be stored electronically for this study on the Forte Research (Oncore) secure server. Data forms will not differ from the paper versions with the exception of an electronic format containing the UNMC Fred & Pamela Buffett Cancer Center and Forte logo.

Forte Research places significant emphasis on providing useful tools in the form of features and functionality in our products that institutions can choose to adopt i n the development of an overallcompliance program. This document outlines the OnCore technical controls that may be implemented to support your institutions Federal Information Security Management Act of 2002 (FISMA) and Federal Information Processing Standards (FIPS) compliance requirements.

OnCore makes available many application capabilities for incorporation within a broader research compliance program. OnCore offers internal security safeguards, external security safeguards, and other system features as outlined in this document, that are managed by your institutions OnCore administrator(s). See attached Oncore FISMA and FIPS Reg compliance controls document

12.0 PATIENT CONSENT

12.1 Human Subjects Research Protection Training

All personnel involved in this research project will have completed the OHRP-approved computer based training course on the Protection of Human Research Subjects. All clinical and correlative research included in this application will have approval by the institutional review board.

12.2 Study Population

Patients are from all socio-economic groups and will be entered into the study without bias with respect to gender or race. Attempts will be made to recruit minorities. No vulnerable subjects will be included in the study.

12.3 Sources of Material

Pathology material (frozen tissue if available, if not then 5-6 unstained slides or a block) must be reviewed, and the diagnosis confirmed by University Nebraska Medical Center pathology department as outlined in the protocol.(retrospectively)

12.4 Recruitment and Informed Consent

Patients with an initial diagnosis of locally advanced pancreatic carcinoma seen and evaluated at The Nebraska Medical Center (TNMC) will be available for recruitment These patients will be informed of the nature of this study, and will be asked to participate on a voluntary basis after informing them of the possible risks and benefits of the study. A number of public registries may be accessible to health care providers and prospective subjects as listed below.

National Library of Medicine - http://clinicaltrials.gov (NCT01336933)
National Cancer Institute - http://www.cancer.gov (NCI-2011-00254)

12.5 Subject Competency

Subjects will be eligible to participate in the study only if they are competent to give informed consent. A subject that the investigators judges to be incompetent will not be enrolled.

12.6 Process of Informed Consent

If the patient chooses to be a participant in this study, informed consent will be obtained by the investigators. The study and procedures involved including the risks will be explained in detail to each subject. It will be clearly explained to the subject that this is a research study and that participation is entirely on a voluntary basis. Subjects will be given the option to discuss the study with a family member, friend, counselor or, another physician. The participating investigators will be available to discuss the study with them.

12.7 Subject/Representative Comprehension

When the process of informed consent is completed, the subject will be asked to state in his/her own words, the purpose of the study, the procedures that will be carried out, potential risk, potential benefits to the subject, the alternatives and the right to withdraw from the study. If there is any indication that a given subject's comprehension is anything less than accurate, the points of confusion will be discussed and clarified.

12.8 Information Purposely Withheld.

The results of the tests done solely for research purposes will not be disclosed to the subject. No other information will be purposely withheld from the subject.

12.9 Potential Benefits of the Proposed Research to the Subjects

It is anticipated that the use of neoadjuvant therapy in this patient population would result in greater tumor response and possibly prolong survival. The neoadjuvant therapy might convert patients with locally advanced disease to a resectable status.

12.10 Potential Benefits to Society.

Information obtained from this study may help other patients by contributing to the knowledge of the biology of pancreatic cancer, and whether this treatment offers potential advantages over other treatments currently available.

12.11 Potential Risks

The use of cytototoxic chemotherapy, external beam radiotherapy, and surgical resection are associated with numerous potential risks. Combined chemotherapy/radiation is considered a valid treatment option for patients with locally advanced pancreatic cancer who are not surgical candidates. Adjuvant therapy following surgical resection for pancreatic cancer is considered a valid treatment option. It is believed the treatment option outlined in the study will not pose significant additional risks compared to conventional treatment.

12.12 Therapeutic Alternatives

If patients choose not to participate in this study they may elect to receive standard therapy as per their primary oncologist, which may include surgery, chemotherapy, or radiation, or a combination of these approaches. The treatment recommendations may or may not be similar to treatment as described in this protocol (pre-operative chemotherapy, followed by external beam radiotherapy and nelfinavir), followed by tumor resection and additional chemotherapy). As yet, there is no proven benefit to the use of neoadjuvant chemotherapy and chemotherapy/radiation prior to surgery in this patient population, and the use of the SRT and nelfinavir as outlined in this protocol document is considered investigational.

12.13 Risk/Benefit Relationship

Although there are inherent risks involved because of the use of chemotherapy, radiotherapy in combination with surgical resection, we anticipate that patients who receive the treatment phase of the protocol will do no worse than expected with standard therapy, and may experience an improved outcome. The risk is considered to be acceptable in the setting of cancer.

12.14 Consent Form Documents

No information will be purposely withheld from the patients. The consent document used in this study will include the adult consent document. See attached consent form

13.0 REFERENCES

1. American Cancer Society. *Cancer Facts & Figures 2014*. Atlanta: American Cancer Society; 2014.
2. National Comprehensive Cancer Network. Pancreatic adenocarcinoma.2016. Available at: http://www.nccn.org/professionals/physician_gls/pdf/pancreatic.pdf. Accessed January 4, 2016.
3. Vincent A, Herman J, Schulick R, et al. Pancreatic cancer. *Lancet*.2011;378:607–620.
4. Willett CG, Lewandrowski K, Warshaw AL, et al. Resection margins in carcinoma of the head of the pancreas. Implications for radiation therapy. *Ann Surg*. 1993;217:144–148.
5. Evans DB, Pisters PW, Lee JE, et al. Preoperative chemoradiation strategies for localized adenocarcinoma of the pancreas. *J Hepatobiliary Pancreat Surg*. 1998;5:242–250.
6. Van den Broeck A, Sergeant G, Ectors N, et al. Patterns of recurrence after curative resection of pancreatic ductal adenocarcinoma. *Eur J Surg Oncol*. 2009;35:600–604.

7. Hattangadi JA, Hong TS, Yeap BY, et al. Results and patterns of failure in patients treated with adjuvant combined chemoradiation therapy for resected pancreatic adenocarcinoma. *Cancer*. 2009;115:3640–3650.
8. Gillen S, Schuster T, Meyer Zum Buschenfelde C, et al. Preoperative/neoadjuvant therapy in pancreatic cancer: a systematic review and meta-analysis of response and resection percentages. *PLoS Med*. 2010;7:e1000267.
9. Raut CP, Evans DB, Crane CH, et al. Neoadjuvant therapy for resectable pancreatic cancer. *Surg Oncol Clin N Am*. 2004;13:639–661.
10. Wayne JD, Abdalla EK, Wolff RA, et al. Localized adenocarcinoma of the pancreas: the rationale for preoperative chemoradiation. *Oncologist*. 2002;7:34–45.
11. Sohn TA, Yeo CJ, Cameron JL, et al. Resected adenocarcinoma of the pancreas—616 patients: results, outcomes, and prognostic indicators. *J Gastroenterol Surg*. 2000;4:567–579.
12. Klinkenbijl JH, Jeekel J, Sahmoud T, et al. Adjuvant radiotherapy and 5-fluorouracil after curative resection of cancer of the pancreas and periampullary region: phase III trial of the EORTC gastrointestinal tract cancer cooperative group. *Ann Surg*. 1999; 230:776–784.
13. Spitz FR, Abbruzzese JL, Lee JE, et al. Preoperative and postoperative chemoradiation strategies in patients treated with pancreaticoduodenectomy for adenocarcinoma of the pancreas. *J Clin Oncol*. 1997;15:928–937.
14. Lim KH, Chung E, Khan A, et al. Neoadjuvant therapy of pancreatic cancer: the emerging paradigm? *Oncologist*. 2012;17:192–200.
15. Brizel DM, Sibley GS, Prosnitz LR, et al. Tumor hypoxia adversely affects the prognosis of carcinoma of the head and neck. *Int J Radiat Oncol Biol Phys*. 1997;38:285–289.
16. Tachezy M, Gebauer F, Petersen C, et al. Sequential neoadjuvant chemoradiotherapy (CRT) followed by curative surgery vs. primary surgery alone for resectable, non-metastasized pancreatic adenocarcinoma: NEOPA- a randomized multicenter phase III study (NCT01900327, DRKS00003893, ISRCTN82191749). *BMC Cancer*. 2014;14:411.
17. Pisters PW, Hudec WA, Lee JE, Raijman I, Lahoti S, Janjan NA, et al. Preoperative chemoradiation for patients with pancreatic cancer: toxicity of endobiliary stents. J Clin Oncol. 2000 Feb;18(4):860-7.
18. Breslin TM, Hess KR, Harbison DB, Jean ME, Cleary KR, Dackiw AP, et al. Neoadjuvant chemoradiotherapy for adenocarcinoma of the pancreas: treatment variables and survival duration. Ann Surg Oncol. 2001 Mar;8(2):123-32.
19. Pisters PW, Abbruzzese JL, Janjan NA, Cleary KR, Charnsangavej C, Goswitz MS, et al. Rapid-fractionation preoperative chemoradiation, pancreaticoduodenectomy, and intraoperative radiation therapy for resectable pancreatic adenocarcinoma. J Clin Oncol. 1998 Dec;16(12):3843-50.
20. Schellenberg D, Goodman KA, Lee F, Chang S, Kuo T, Ford JM, et al. Gemcitabine chemotherapy and single-fraction stereotactic body radiotherapy for locally advanced pancreatic cancer. Int J Radiat Oncol Biol Phys. 2008 Nov 1;72(3):678-86.
21. Mahadevan A, Jain S, Goldstein M, Miksad R, Pleskow D, Sawhney M, et al. Stereotactic body radiotherapy and gemcitabine for locally advanced pancreatic cancer. Int J Radiat Oncol Biol Phys. Nov 1;78(3):735-42.
22. Conroy, T., Desseigne, F., Ychou, M., et al.. FOLFIRINOX versus Gemcitabine for Metastatic Pancreatic Cancer N Engl J Med 2011;364:1817-25.
23. Von Hoff, D. D., Ervin, E., Arena, FP., et al.. Increased Survival in Pancreatic Cancer with nab-Paclitaxel plus Gemcitabine, N Engl J Med 2013;369:1691-703
24. McGinn CJ, Zalupski MM, Shureiqi I, Robertson JM, Eckhauser FE, Smith DC, et al. Phase I trial of radiation dose escalation with concurrent weekly full-dose gemcitabine in patients with advanced pancreatic cancer. J Clin Oncol. 2001 Nov 15;19(22):4202-8.

25. Muler JH, McGinn CJ, Normolle D, Lawrence T, Brown D, Hejna G, et al. Phase I trial using a time-to-event continual reassessment strategy for dose escalation of cisplatin combined with gemcitabine and radiation therapy in pancreatic cancer. J Clin Oncol. 2004 Jan 15;22(2):238-43.
26. Keall P. 4-dimensional computed tomography imaging and treatment planning. Semin Radiat Oncol. 2004 Jan;14(1):81-90.
27. Ford EC, Mageras GS, Yorke E, Ling CC. Respiration-correlated spiral CT: a method of measuring respiratory-induced anatomic motion for radiation treatment planning. Med Phys. 2003 Jan;30(1):88-97.
28. Low DA, Nystrom M, Kalinin E, Parikh P, Dempsey JF, Bradley JD, et al. A method for the reconstruction of four-dimensional synchronized CT scans acquired during free breathing. Med Phys. 2003 Jun;30(6):1254-63.
29. Pan T, Lee TY, Rietzel E, Chen GT. 4D-CT imaging of a volume influenced by respiratory motion on multi-slice CT. Med Phys. 2004 Feb;31(2):333-40.
30. Rietzel E, Pan T, Chen GT. Four-dimensional computed tomography: image formation and clinical protocol. Med Phys. 2005 Apr;32(4):874-89.
31. Vedam SS, Keall PJ, Kini VR, Mostafavi H, Shukla HP, Mohan R. Acquiring a four-dimensional computed tomography dataset using an external respiratory signal. Phys Med Biol. 2003 Jan 7;48(1):45-62.
32. Wink N, Panknin C, Solberg TD. Phase versus amplitude sorting of 4D-CT data. J Appl Clin Med Phys. 2006 Winter;7(1):77-85.
33. Litzenberg DW, Balter JM, Lam KL, Sandler HM, Ten Haken RK. Retrospective analysis of prostate cancer patients with implanted gold markers using off-line and adaptive therapy protocols. Int J Radiat Oncol Biol Phys. 2005 Sep 1;63(1):123-33.
34. Kupelian PA, Willoughby TR, Meeks SL, Forbes A, Wagner T, Maach M, et al. Intraprostatic fiducials for localization of the prostate gland: monitoring intermarker distances during radiation therapy to test for marker stability. Int J Radiat Oncol Biol Phys. 2005 Aug 1;62(5):1291-6.
35. Kitamura K, Shirato H, Shimizu S, Shinohara N, Harabayashi T, Shimizu T, et al. Registration accuracy and possible migration of internal fiducial gold marker implanted in prostate and liver treated with real-time tumor-tracking radiation therapy (RTRT). Radiother Oncol. 2002 Mar;62(3):275-81.
36. Imura M, Yamazaki K, Shirato H, Onimaru R, Fujino M, Shimizu S, et al. Insertion and fixation of fiducial markers for setup and tracking of lung tumors in radiotherapy. Int J Radiat Oncol Biol Phys. 2005 Dec 1;63(5):1442-7.
37. de Mey J, Van de Steene J, Vandenbroucke F, Verellen D, Trappeniers L, Meysman M, et al. Percutaneous placement of marking coils before stereotactic radiation therapy of malignant lung lesions. J Vasc Interv Radiol. 2005 Jan;16(1):51-6.
38. Koong AC, Le QT, Ho A, Fong B, Fisher G, Cho C, et al. Phase I study of stereotactic radiosurgery in patients with locally advanced pancreatic cancer. Int J Radiat Oncol Biol Phys. 2004 Mar 15;58(4):1017-21.
39. Boothman DA, Greer S, Pardee AB (1987) Potentiation of halogenated pyrimidine radiosensitizers in human carcinoma cells by beta-lapachone (3,4-dihydro-2,2-dimethyl-2H-naphtho[1,2-b]pyran- 5,6-dione), a novel DNA repair inhibitor. Cancer Res 47(20): 5361–5366.
40. Macaulay VM, Salisbury AJ, Bohula EA, Playford MP, Smorodinsky NI, Shiloh Y (2001) Downregulation of the type 1 insulin-like growth factor receptor in mouse melanoma cells is associated with enhanced radiosensitivity and impaired activation of Atm kinase. Oncogene 20(30):4029–4040.
41. Munshi A, Kurland JF, Nishikawa T, Chiao PJ, Andreeff M, Meyn RE (2004) Inhibition of constitutively activated nuclear factor-kappaB radiosensitizes human melanoma cells. Mol Cancer Ther 3(8): 985–992.

42. Kimple RJ, Vaseva AV, Cox AD, Baerman KM, Calvo BF, Tepper JE, Shields JM, Sartor CI (2010) Radiosensitization of epidermal growth factor receptor/HER2-positive pancreatic cancer is mediated by inhibition of Akt independent of ras mutational status. Clin Cancer Res 16(3): 912–923.
43. Hui Z, Tretiakova M, Zhang Z, Li Y, Wang X, Zhu JX, Gao Y, Mai W, Furge K, Qian CN, Amato R, Butler EB, Teh BT, Teh BS (2009) Radiosensitization by inhibiting STAT1 in renal cell carcinoma. Int J Radiat Oncol Biol Phys 73(1): 288–295.
44. Wolff RA, Chiao P, Lenzi R, Pisters PW, Lee JE, Janjan NA, Crane CH, Evans DB, Abbruzzese JL. Current approaches and future strategies for pancreatic carcinoma. Invest New Drugs 2000;18:43-56.
45. Ghaneh P, Costello E, Neoptolemos JP. Biology and management of pancreatic cancer. Postgrad Med J 2008;84:478-97.
46. Hazard L (2009) The role of radiation therapy in pancreas cancer. Gastrointest Cancer Res 3(1): 20–28.
47. Roldan GE, Gunderson LL, Nagorney DM, Martin JK, Ilstrup DM, Holbrook MA, Kvols LK, McIlrath DC (1988) External beam versus intraoperative and external beam irradiation for locally advanced pancreatic cancer. Cancer 61(6): 1110–1116.
48. Schwartz DL, Bankson JA, Lemos R, Jr., Lai SY, Thittai AK, He Y, Hostetter G, Demeure MJ, Von Hoff DD, Powis G. Radiosensitization and stromal imaging response correlates for the HIF-1 inhibitor PX-478 given with or without chemotherapy in pancreatic cancer. Mol Cancer Ther 2010;9:2057-67.
49. Eguchi H, Ishikawa O, Ohigashi H, Takahashi H, Yano M, Nishiyama K, Tomita Y, Uehara R, Takehara A, Nakamura Y, Nakagawa H. Serum REG4 level is a predictive biomarker for the response to preoperative chemoradiotherapy in patients with pancreatic cancer. Pancreas 2009;38:791-8.
50. Souchek, J., Baine, M., Lin, C., Rachagani, S., Gupta, S., Kaur, S., Lester, K., Zheng, D., Chen, S., Smith, L., Lazenby, A., Johansson, S.L., Jain, M., and Batra, S. "Unbiased analysis of pancreatic cancer radiation resistance reveals cholesterol biosynthesis as a novel target for radiosensitization" British Journal of Cancer, (15 July 2014) doi:10.1038/bjc.2014.385
51. Denham JW and Steigler A. Picking the Optimal Duration of Hormonal Therapy in Men With High-Risk and Locally Advanced Prostate Cancer Treated With Radiotherapy Semin Radiat Oncol 23:206-214, 2013
52. Brufsky A, Harker WG, Beck JT, Carroll R, Tan-Chiu E, Seidler C, Hohneker J, Lacerna L, Petrone S, Perez EA. Zoledronic acid inhibits adjuvant letrozole-induced bone loss in postmenopausal women with early breast cancer. J Clin Oncol 2007;25:829-36
53. Tenn SE, Solberg TD, Medin PM. Targeting accuracy of an image guided gating system for stereotactic body radiotherapy. Phys Med Biol 2005; 50:5443-62.
54. Kris MG, Hesketh PJ, Somerfield MR, Feyer P, Clark-Snow R, Koeller JM, et al. American Society of Clinical Oncology guideline for antiemetics in oncology: update 2006. J Clin Oncol. 2006 Jun 20;24(18):2932-47.
55. Smith TJ, Khatcheressian J, Lyman GH, Ozer H, Armitage JO, Balducci L, et al. 2006 update of recommendations for the use of white blood cell growth factors: an evidence-based clinical practice guideline. J Clin Oncol. 2006 Jul 1;24(19):3187-205.
56. Rizzo JD, Lichtin AE, Woolf SH, Seidenfeld J, Bennett CL, Cella D, et al. Use of epoetin in patients with cancer: evidence-based clinical practice guidelines of the American Society of Clinical Oncology and the American Society of Hematology. J Clin Oncol. 2002 Oct 1;20(19):4083-107.
57. Lin C, Chen S, Baine MJ. "Set-up error correction using internal markers on patients with pancreatic adenocarcinoma receiving stereotactic body radiotherapy and its association with the

patient's body mass index". Modern Practices in Radiation Therapy, 2012;ISBN 979-953-307-341-4.
58. Lin C, Sasson AR, Ly QP, Kos ME, Schwarz JK, Are C, Chen S, Sehi ED, Enke CA, Grem JL. A Phase 1 Study of Hypofractionated Stereotactic Radiotherapy and Concurrent HIV Protease Inhibitor Nelfinavir as part of a Neoadjuvant Regimen in Patients with Locally Advanced Pancreatic. Int J Rad Onc Biol Phys 2011;81: S68 (abstr 133).
59. Lin C, Lazenby AJ, Hussain SM, Sasson AR, Chen S, Ly QP, Schwarz JK, Kos ME, re C, em JL. Borderline Resectable Pancreatic Adenocarcinoma: Tumor Volume and CA19-9 Reduction Following Neoadjuvant Chemotherapy and Fractionated Stereotactic Body Radiotherapy Correlates with Pathologic Response. Int J Rad Onc Biol Phys 2012;84: S320 (abstr 2270).
60. Lin C, Chen S, Enke CA, Zhou S. Set-up Error Correction Using Internal Markers on Patients with Pancreatic Adenocarcinoma Receiving Stereotactic Body Radiotherapy and it's Association with the Patient's Body Mass Index. Int J Rad Onc Biol Phys 2011;81: S333 (abstr 2252).
61. Chen S, Lin C, Schubert L, Enke CA, Zhou S. Using 4D-CT Images and Fiducial Markers to Quantify Respiratory-Induced Pancreatic Head Tumor Motion for Patients Receiving Stereotactic Body Radiation Therapy (SBRT). Int J Rad Onc Biol Phys 2011;S340 (abstr 2267).
62. Chen S, Lin C, Enke CA, Zhou S. Respiratory-induced Setup Errors Should Be Considered for Patients with Tumor Affected by Respiratory Motion and Treated with free breathing IGRT: A pilot study on Patients with Pancreatic Head tumor. Int J Rad Onc Biol Phys 2013;84: S720 (abstr 3337).
63. Chen S, Lin C, Schubert L, Deng H, Enke CA, Zhou S. Does Pancreatic Head Tumor Motion on the 4DCT Accurately Predict the Tumor Motion During the Treatment for Patients Receiving Gated Stereotactic Body Radiation Therapy (SBRT). Int J Rad Onc Biol Phys 2011; 81: S87 (abstr 173).
64. Ford EC, Herman J, Yorke E, Wahl RL. (18)F-FDG PET/CT for image-guided and intensity-modulated radiotherapy. *J Nucl Med* 2009; 50:1655-1665
65. Topkan E, Yavuz AA, Aydin M, Onal C, Yapar F, Yavuz MN. Comparison of CT and PET-CT based planning of radiation therapy in locally advanced pancreatic carcinoma. *J Exp Clin Cancer Res* 2008; 27:41
66. Rose DM, Delbeke D, Beauchamp RD, et al. ^{18}Fluorodeoxyglucose-positron emission tomography in the management of patients with suspected pancreatic cancer. *Ann Surg* 1999; 229:729-737; discussion 737-738
67. Higashi T, Sakahara H, Torizuka T, et al. Evaluation of intraoperative radiation therapy for unresectable pancreatic cancer with FDG PET. *J Nucl Med* 1999; 40:1424-1433
68. Maisey NR, Webb A, Flux GD, et al. FDG-PET in the prediction of survival of patients with cancer of the pancreas: a pilot study. *Br J Cancer* 2000; 83:287-293
69. Yoshioka M, Sato T, Furuya T, et al. Role of positron emission tomography with 2-deoxy-2-[F-18] fluoro-d-glucose in evaluating the effects of arterial infusion chemotherapy and radiotherapy on pancreatic cancer. *J Gastroenterol* 2004; 39:50-55
70. Bang S, Chung HW, Park SW, et al. The clinical usefulness of 18-fluorodeoxyglucose positron emission tomography in the differential diagnosis, staging, and response evaluation after concurrent chemoradiotherapy for pancreatic cancer. *J Clin Gastroenterol* 2006; 40:923-929

71. Kuwatani M, Kawakami H, Eto K, et al. modalities for evaluating chemotherapeutic efficacy and survival time in patients with advanced pancreatic cancer: comparison between FDG-PET, CT, and serum tumor markers. *Intern Med* 2009; 48:867-875

72. Cameron K, Golan S, Simpson W, et al. Recurrent pancreatic carcinoma and holangiocarcinoma: 18F-fluorodeoxyglucose positron emission tomography/computed tomography (PET/CT). bdom Imaging 2011; 36:463-471

73. Kachnic, LA, Pugh, SL, Tai, P, Smith, et al. RTOG 0518: Randomized Phase III Trial to valuate Zoledronic Acid for Prevention of Osteoporosis and Associated Fractures in Prostate Cancer Patients. Prostate Cancer Prostatic Dis. 2013 December ; 16(4):383-6

14.0 DATA FORMS Attached

APPENDIX A
Criteria Defining Resectability Status

NCCN Clinical Practice Guidelines in Oncology™ Version 2.2011

RESECTABLE

HEAD/BODY/TAIL

1) **No distant metastases**
2) **No radiographic evidence of superior mesenteric arteries (SMA) and portal vein abutment, distortion, tumor thrombus, or venous encasement**
3) **Clear fat plane around celiac axis, hepatic artery, and SMA.**

BORDERLINE RESECTABLE

1) **No distant metastases**
2) **Venous involvement of SMV/portal vein demonstrating tumor abutment with impringement and narrowing of lumen, encasement of the SMV/portal vein but without encasement of the nearby arteries, or short segment venous occlusion resulting from either tumor thrombus or encasement but with suitable vessel proximal and distal to the area of vessel involvement, allowing for safe resection and reconstruction.**
3) **Gastroduodenal artery encasement up to the hepatic artery with either short segment encasement or direct abutment of the hepatic artery, without extension to the celiac axis.**
4) **Tumor abutment of the SMA not to exceed greater than 180 degrees of the circumference of the vessel wall.**

UNRESECTABLE

HEAD

1) **Distant metastases**
2) **Greater than 180 degrees SMA encasement, any celiac abutment**
3) **Unreconstructable SMV/portal occlusion**
4) **Aortic invasion or encasement**

BODY

1) **Distant metastases**
2) **SMA or celiac encasement greater than 180 degrees**
3) **Unreconstructable SMV/portal occlusion**
4) **Aortic invasion**

TAIL

a. **Distant metastases**
b. **SMA or celiac encasement greater than 180 degrees**

NODAL STATUS

1) **Metastases to lymph nodes beyond the field of resection should be considered unresectable.**

APPENDIX B
Performance Status Criteria

ECOG Performance Status Scale		Karnofsky Performance Scale	
Grade	Descriptions	Percent	Description
0	Normal activity. Fully active, able to carry on all pre-disease performance without restriction.	100	Normal, no complaints, no evidence of disease.
		90	Able to carry on normal activity; minor signs or symptoms of disease.
1	Symptoms, but ambulatory. Restricted in physically strenuous activity, but ambulatory and able to carry out work of a light or sedentary nature (e.g., light housework, office work).	80	Normal activity with effort; some signs or symptoms of disease.
		70	Cares for self, unable to carry on normal activity or to do active work.
2	In bed <50% of the time. Ambulatory and capable of all self-care, but unable to carry out any work activities. Up and about more than 50% of waking hours.	60	Requires occasional assistance, but is able to care for most of his/her needs.
		50	Requires considerable assistance and frequent medical care.
3	In bed >50% of the time. Capable of only limited self-care, confined to bed or chair more than 50% of waking hours.	40	Disabled, requires special care and assistance.
		30	Severely disabled, hospitalization indicated. Death not imminent.
4	100% bedridden. Completely disabled. Cannot carry on any self-care. Totally confined to bed or chair.	20	Very sick, hospitalization indicated. Death not imminent.
		10	Moribund, fatal processes progressing rapidly.
5	Dead.	0	Dead.

APPENDIX C
Eligibility Checklist

Date Completed:	Institution: UNMC Eppley Cancer Center	Patient ID:	Date of Birth
IRB# xxx-16 Title: A Phase I/II Study of Hypofractionated Stereotactic Radiotherapy and Concurrent Zometa in Patients with Locally Advanced Pancreatic Adenocarcinoma		UNMC MRN:	
Last Name:		First Name:	
Gender: [ ] M [ ] F	Race: [ ] White [ ] Black [ ] Hispanic [ ] Asian [ ] Native American [ ] Other [ ] Unknown		
Zip Code/country (if not USA):	Primary method of payment information:		

Inclusion Criteria: **Response should be YES**	Yes No N/A
1. Is a cancer patient with pathologically confirmed adenocarcinoma of the pancreas? The maximum dimension of the tumor must be ≤ 10 cm.	[ ] [ ] [ ] 1.
2. Is the patient 19 years of age or older? Enter Age:___	[ ] [ ] [ ]
3. Is the Karnofsky Performance Status 60% or better? Enter PS: _______	[ ] [ ] [ ]
4. Is the patient who received chemotherapy > 5 years ago for malignancies other than pancreatic cancer eligible, provided that chemotherapy was completed > 5 years ago and that there is no evidence of the second malignancy at the time of study entry?	[ ] [ ] [ ]
5. Is the patient who received radiation therapy > 5 years ago for malignancies other than pancreatic cancer and whose radiation therapy field is not overlapping with the 20% isodose line of current radiation field eligible, provided that radiation therapy was completed > 5 years ago and that there is no evidence of the second malignancy at the time of study entry?	[ ] [ ] [ ]
6. All malignant disease must be able to be encompassed within a single irradiation field?	[ ] [ ] [ ]
7. Is the patient disease radiographically assessable ?	[ ] [ ] [ ]
8. Is the absolute neutrophil count 1,500 per mcL or higher and the platelet count is 100,000 per mCL or higher?<br>Enter ANC: ______ Enter Platelet count: ________	[ ] [ ] [ ]
9. Is the serum creatinine at or below 2 mg/dL and total bilirubin at or below 2.0 mg per dL in the absence of biliary obstruction?(If the patient has biliary obstruction, biliary decompression will be required. Either endoscopic placement of a biliary stent or percutaneous transhepatic drainage is acceptable. Once biliary drainage has been established, institution of protocol therapy may proceed when the total bilirubin falls to 4.0 mg/dL or lower.) Enter creatinine: ______ Enter bilirubin: ______	[ ] [ ] [ ]
10. The patient is aware of the neoplastic nature of his/her disease and willingly provide written, informed consent after being informed of the procedure to be followed, the experimental nature of the therapy, alternatives, potential benefits, side-effects, risks, and discomforts.	[ ] [ ] [ ]
11. No prior therapy with the exception of chemotherapy based on current diagnosis and clinical condition.	[ ] [ ] [ ]
Exclusion Criteria: **Response should be NO**	Yes No N/A
1. Patient cannot undergo marker implantation (which may consist of a surgical clip, a gold clip or a common bile duct stent next to the tumor)?	[ ] [ ] [ ]
2. Does the patient have known allergy to murine proteins or had a documented anaphylactic reaction or allergy to Zometa, or to antiemetics appropriate for administration in conjunction with protocol-directed therapy?	[ ] [ ] [ ]
3. Does the patient have a history of uncontrolled inter-current illness including, but not limited to ongoing or active infection requiring intravenous antibiotics, symptomatic congestive heart failure, unstable angina pectoris, or serious, uncontrolled cardiac arrhythmia, that might jeopardize the ability of the patient to receive the chemotherapy program outlined in this protocol with reasonable safety?	[ ] [ ] [ ]
4. Is the patient pregnant or breast feeding?	[ ] [ ] [ ]

5. Does the patient have a history of prior malignancy **except for** adequately treated basal cell or squamous cell skin cancer, adequately treated noninvasive carcinomas, or other cancers from which the patient has been disease-free for at least 5 years?	[ ] [ ] [ ]
6. Does the patient have active duodenal ulcer or bleeding or history of a gastrointestinal fistula or perforation or other significant bowel problems (severe nausea, vomiting, inflammatory bowel disease and significant bowel resection)?	[ ] [ ] [ ]
7. Does the patient known to have hepatic insufficiency?	[ ] [ ] [ ]
8. Is the patient allergic to 5FU/capecitabine or Zometa?	[ ] [ ] [ ]
9. Is the patient receiving or have they received any other investigational agents during/or within 1 month prior to treatment with Zometa?	[ ] [ ] [ ]

NOTE: All questions regarding eligibility for potential subjects should be directed to the UNMC Coordinator at 402-552-2790 (Amy) or 402-559-8649 (Terry).

Eligibility: [ ] Patient satisfies all criteria
[ ] Patient not formally eligible, but admitted to this study because (state reason):

____

____

ELIGIBILITY reviewed and confirmed.

Investigator Signature ________________________Date_____________

Printed Name of Investigator: _________________________

Version 4 (6/1/2017)

APPENDIX D
Study Blood Specimens

Six- 10mL Green top (whole blood) tubes ARM B

Intended Time	Date of Sample obtained	Actual Time sample Drawn
0 hour (At the time Zometa is given)		
1 hour		
Day 2 Radiation Therapy		
Day 3 Radiation Therapy		
Day 4 Radiation Therapy		
Day 5 Radiation Therapy		

TWO- 10ml Green top (whole blood) tubes ARM A

Intended Time	Date of Sample obtained	Actual Time sample Drawn
0 hour (prior to radiation treatment)		
Day 5 (after radiation treatment)		

APPENDIX E

<u>NCI Common Toxicity Criteria Version 4.03 (CTCAE)</u>
Active Date: June 14, 2010

Toxicity will be scored using NCI CTC Version 4.03 for toxicity and adverse event reporting. A copy of the NCI CTC Version 4.03 can be downloaded from the CTEP homepage: (http://ctep.info.nih.gov). All appropriate treatment areas have access to a copy of the CTC Version 4.03.

APPENDIX F

FDA MEDWATCH form

Available on-line at http://www.fda.gov/medwatch/SAFETY/3500.pdf
