# Supplementary material for: Disruption of FDPS/Rac1 axis radiosensitizes pancreatic ductal adenocarcinoma by attenuating DNA damage response and immunosuppressive signalling
Source: eBioMedicine. 2021 Dec 28;75:103772. doi: 10.1016/j.ebiom.2021.103772 (PMC8718746; doi:10.1016/j.ebiom.2021.103772)
Supplement: Supplementary file 3 [file mmc3.pdf]

CONSORT 2010 checklist of information to include when reporting a randomised trial*

Section/Topic	Item No	Checklist item	Reported on page No
**Title and abstract**			
	1a	Identification as a randomised trial in the title	Page 13, line 303
	1b	Structured summary of trial design, methods, results, and conclusions (for specific guidance see CONSORT for abstracts)	Page 2
**Introduction**			Page 2 and 5 , Abstract
Background and objectives	2a	Scientific background and explanation of rationale	Page 5 and 13, line 131-135 and lines 316-320
	2b	Specific objectives or hypotheses	
**Methods**			
Trial design	3a	Description of trial design (such as parallel, factorial) including allocation ratio	Page 13, line 303-314
	3b	Important changes to methods after trial commencement (such as eligibility criteria), with reasons	Page 13, line 303-305
Participants	4a	Eligibility criteria for participants	Page 13, line 303-305
	4b	Settings and locations where the data were collected	Page 6, line 140-141
Interventions	5	The interventions for each group with sufficient details to allow replication, including how and when they were actually administered	Fig. S15
Outcomes	6a	Completely defined pre-specified primary and secondary outcome measures, including how and when they were assessed	2.14, Page 13, Lines 316-320
	6b	Any changes to trial outcomes after the trial commenced, with reasons	Northing to report
Sample size	7a	How sample size was determined	
	7b	When applicable, explanation of any interim analyses and stopping guidelines	None
Randomisation:			
Sequence generation	8a	Method used to generate the random allocation sequence	2.15, Page 14, Lines 321-333
	8b	Type of randomisation; details of any restriction (such as blocking and block size)	None
Allocation concealment mechanism	9	Mechanism used to implement the random allocation sequence (such as sequentially numbered containers), describing any steps taken to conceal the sequence until interventions were assigned	2.15, Page 14, Lines 324-329
Implementation	10	Who generated the random allocation sequence, who enrolled participants, and who assigned participants to interventions	Dr.Smith (Statistician) and Dr. Chi Lin (Radiation Oncologist)
Blinding	11a	If done, who was blinded after assignment to interventions (for example, participants, care providers, those	Participants, Statistician and pathologist

Section/Topic	Item No	Checklist item	Reported on page No
		assessing outcomes) and how	Supplementary materials-Ref clinical trial protocol
	11b	If relevant, description of the similarity of interventions	Supplementary materials-Ref clinical trial protocol
Statistical methods	12a	Statistical methods used to compare groups for primary and secondary outcomes	Section 2.15, page 14
	12b	Methods for additional analyses, such as subgroup analyses and adjusted analyses	Supplementary materials-Ref clinical trial protocol
**Results**			
Participant flow (a diagram is strongly recommended)	13a	For each group, the numbers of participants who were randomly assigned, received intended treatment, and were analysed for the primary outcome	Fig. S15
	13b	For each group, losses and exclusions after randomisation, together with reasons	Supplementary materials-Ref clinical trial protocol
Recruitment	14a	Dates defining the periods of recruitment and follow-up	Supplementary materials-Ref clinical trial protocol
	14b	Why the trial ended or was stopped	Trial is ongoing not ended, Ref. 10.6 -Trial protocol
Baseline data	15	A table showing baseline demographic and clinical characteristics for each group	Not Included
Numbers analysed	16	For each group, number of participants (denominator) included in each analysis and whether the analysis was by original assigned groups	Section 10, Clinical trial protocol
Outcomes and estimation	17a	For each primary and secondary outcome, results for each group, and the estimated effect size and its precision (such as 95% confidence interval)	Section 6, Clinical Trial protocol
	17b	For binary outcomes, presentation of both absolute and relative effect sizes is recommended	Not applicable
Ancillary analyses	18	Results of any other analyses performed, including subgroup analyses and adjusted analyses, distinguishing pre-specified from exploratory	Not specified
Harms	19	All important harms or unintended effects in each group (for specific guidance see CONSORT for harms)	Section 6.5 and 6.6 - Clinical trial protocol
**Discussion**			
Limitations	20	Trial limitations, addressing sources of potential bias, imprecision, and, if relevant, multiplicity of analyses	Page 31, line 732-737
Generalisability	21	Generalisability (external validity, applicability) of the trial findings	Page 29, line 672-675
Interpretation	22	Interpretation consistent with results, balancing benefits and harms, and considering other relevant evidence	Page 31, line 711-719
**Other information**			
Registration	23	Registration number and name of trial registry	NCT03073785, Hypofractionated Stereotactic Body Radiation Therapy and Fluorouracil or Capecitabine With or Without Zoledronic Acid in Treating Patients With Locally Advanced Pancreatic Cancer
Protocol	24	Where the full trial protocol can be accessed, if available	Attached with Supplementary materials-Ref clinical trial protocol
Funding	25	Sources of funding and other support (such as supply of drugs), role of funders	Ref. Section 2.21 in methods and SPORE, R01 and U01 grants are the source for funding

*We strongly recommend reading this statement in conjunction with the CONSORT 2010 Explanation and Elaboration for important clarifications on all the items. If relevant, we also recommend reading CONSORT extensions for cluster randomised trials, non-inferiority and equivalence trials, non-pharmacological treatments, herbal interventions, and pragmatic trials. Additional extensions are forthcoming: for those and for up to date references relevant to this checklist, see www.consort-statement.org.
